# The N-terminus of CXCR4 splice variants determines expression and functional properties

**DOI:** 10.1371/journal.pone.0283015

**Published:** 2023-05-04

**Authors:** Hee-Kyung Park, Lan Phuong Nguyen, Thai Uy Nguyen, Minyeong Cho, Huong Thi Nguyen, Sunghoon Hurh, Hong-Rae Kim, Jae Young Seong, Cheol Soon Lee, Byung-Joo Ham, Jong-Ik Hwang

**Affiliations:** 1 Department of Biomedical Sciences, College of Medicine, Korea University, Seoul, Republic of Korea; 2 Department of Psychiatry, College of Medicine, Korea University, Seoul, Republic of Korea; University of Illinois at Chicago College of Medicine, UNITED STATES

## Abstract

C-X-C motif chemokine ligand 12(CXCL12) is an essential chemokine for organ development and homeostasis in multiple tissues. Its receptor, C-X-C chemokine receptor type 4(CXCR4), is expressed on the surface of target cells. The chemokine and receptor are expressed almost ubiquitously in human tissues and cells throughout life, and abnormal expression of CXCL12 and CXCR4 is observed in pathological conditions, such as inflammation and cancer. CXCR4 is reportedly translated into five splicing variants of different lengths, which each have different amino acids in the N-terminus. As the N-terminus is the first recognition site for chemokines, CXCR4 variants may respond differently to CXCL12. Despite these differences, the molecular and functional properties of CXCR4 variants have not been thoroughly described or compared. Here, we explored the expression of CXCR4 variants in cell lines and analyzed their roles in cellular responses using biochemical approaches. RT-PCR revealed that most cell lines express more than one CXCR4 variant. When expressed in HEK293 cells, the CXCR4 variants differed in protein expression efficiency and cell surface localization. Although variant 2 demonstrated the strongest expression and cell surface localization, variants 1, 3, and 5 also mediated chemokine signaling and induced cellular responses. Our results demonstrate that the N-terminal sequences of each CXCR4 variant determine the expression of the receptor and affect ligand recognition. Functional analyses revealed that CXCR4 variants may also affect each other or interact during CXCL12-stimulated cellular responses. Altogether, our results suggest that CXCR4 variants may have distinct functional roles that warrant additional investigation and could contribute to future development of novel drug interventions.

## Introduction

Chemokines are small (8∼12 kDa) secreted cytokines that control directional migration of cells in immune systems and organogenesis [1]. So far, 47 human chemokines have been identified and categorized into four subfamilies based on the location of the first two highly conserved cysteines of the peptide: C, CC, CXC, and CX_3_C [2]. Chemokine receptors belong to the rhodopsin-like G protein-coupled receptor (GPCR) family and activate Gα_i/o_ and Gα_12/13_ pathways to trigger cell migration [3, 4]. Approximately 23 different receptors have been characterized to date [5, 6]. There are redundancies in chemokine–receptor interactions with many chemokines binding to one receptor and vice versa [7, 8]. Cell-specific responses to chemokines are attributed to their cognate receptors expressed on the cell surface. The chemokine–receptor interaction is a two-step process [9]. First, the body of the chemokine binds to the N-terminus of the cognate receptor. Then, a conformational change of the N-terminal region induces invagination of the short sequence in front of the first conserved cysteine residue into the transmembrane binding pocket of the receptor. This binding mechanism reinforces both flexibility and specificity in the chemokine–receptor interaction despite the structural similarity of chemokines. The extremely short N-terminus of CXCL14 may illuminate the importance of the two-step binding process. CXCL14 inhibits CXCL12-CXCR4 interaction without stimulating the receptor. The sequence similarity of CXCL14 and CXCL12 may enable CXCL14 to bind the N-terminus of the CXCR4 but the N-terminal sequence of CXCL14 may be too short to proceed the second step of the binding process [10, 11]. Although the N-termini of receptors recognize chemokines with specificity, some chemokine receptor genes are translated with different N-terminal sequences due to alternative splicing. The ligand binding efficiency of the splicing variants may be affected by these differences in the N-terminal region, and the resulting cellular responses to the chemokine are likely to vary according to the expression patterns of the variants.

CXCL12 is recognized by CXCR4, which transduces signals by activating heterotrimeric G proteins and recruiting β-arrestin; whereas, CXCR7 binds CXCL12 but recruits β-arrestin without G protein activation [12]. Both CXCR4 and CXCL12 are widely expressed in many tissues and cell types, such as brain, thymus, heart, lung, liver, kidney, spleen, bone marrow, etc. [13, 14]. They play critical roles in embryogenesis, homeostasis, and pathological conditions, including inflammation and cancer progression. During embryonic development, CXCR4 and CXCL12 contribute to proliferation and migration of nerve cells to form brain structures, especially in the cerebellum, which has been verified in animal models by gene deletion [15, 16]. In the circulatory system, CXCR4 and CXCL12 drive bone marrow localization of hematopoietic stem cells in embryos and maintain the stem cells in the bone marrow after birth [17]. For this reason, the CXCR4 inhibitor AMD3100 is used clinically to mobilize stem cells from bone marrow for hematopoietic stem cell transfusion [18]. CXCR4 and CXCL12 are also involved in angiogenesis and recruit progenitor cells from bone marrow [13]. CXCL12 is highly expressed in inflamed tissues and recruits immune cells through CXCR4 to increase immune responses or exacerbate inflammation [14]. Increased levels of both CXCL12 and CXCR4 in cancer cells and the related microenvironment are frequently observed and are responsible for cell proliferation and metastasis, indicating their utility as cancer biomarkers [19]. For these reasons, CXCR4 and CXCL12 have been extensively studied to define their precise properties and to develop modulators for CXCR4 [20, 21]. However, most studies have been done with one CXCR4 variant even though there are five splicing variants with different N-terminal sequences, which may impact ligand binding. Only a few studies have contributed to the limited knowledge for some of the CXCR4 variants [22, 23].

We explored expression of CXCR4 variants in several cell lines by RT-PCR with variant-specific primers and analyzed the molecular properties of each variant using both customary methods and advanced technologies, such as NanoLuc Binary Technology (NanoBiT) and CRISPR/Cas9-based gene deletion and reconstitution of each variant. These approaches demonstrated that the N-terminal sequences of CXCR4 are critical for expression level, cell surface localization, and ligand-dependent activation. Furthermore, co-expression of the variants revealed that they may affect each other during CXCL12-stimulated cellular responses including survival and chemotactic migration.

## Results

### Genetic structure and mRNA expression of CXCR4 splicing variants

**Fig 1:**
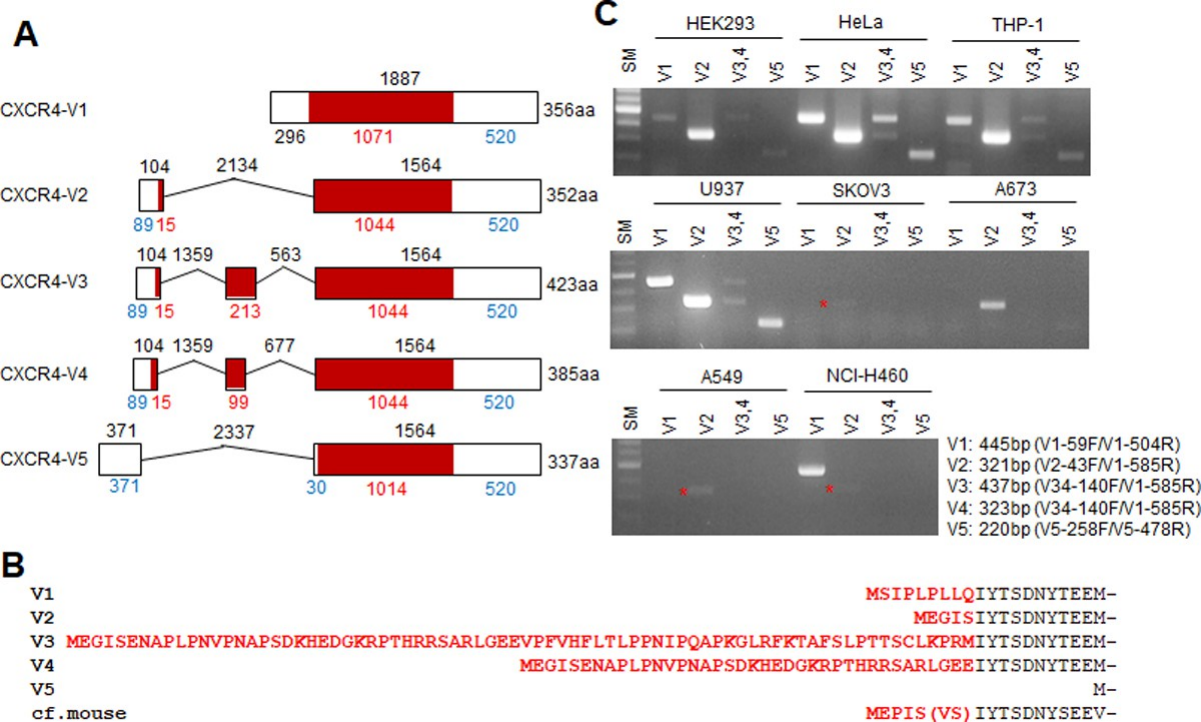
CXCR4 splicing variants. (A) Schematic of CXCR4 variant structure. Boxed regions are exons, and brown indicates translation. Numbers designate the number of nucleotides. Red indicates a translated region, and blue indicates a non-translation region. (B) Amino acid sequences in the foremost region of each variant. Red designates the unique sequences of each variant. The first methionine of variant 5 is shared with the other variants. (C) mRNA expression of the variants. Size marker: 1 kb-plus ladder.

Since human CXCR4 gene was reported for the first time by Bleul et al. in 1996 [24], another variant designated as variant 1 was cloned by Gupta et al. [25]. In 2015, Sand et al. identified variant 3 and 4 of CXCR4 by transcriptome sequencing [22]. According to NCBI nucleotide (https://ncbi.nlm.nih.gov) databases, however, five human CXCR4 splicing variants have been identified and are all likely to be translated into proteins. Analysis of the genetic structure of human CXCR4 revealed that the 5’ region of the gene is a hot spot for alternative splicing, generating variants with different N-terminal amino acid sequences that share the same sequence in other regions (Fig 1A). Two murine CXCR4 transcripts have been reported in NCBI. Both are similar to human CXCR4 variant 2 and are identical to each other with the exception of a two amino acid addition in the splicing region between exon 1 and 2. Therefore, human CXCR4 variant 2 may be the evolutionally conserved variant and have been commonly considered to code for CXCR4. Structural analysis and biochemical studies of many chemokine receptors have indicated that the N-terminus of the receptors plays a pivotal role in the initiation of ligand binding [26, 27]. Thus, we speculate that each CXCR4 variant has distinctive molecular properties. The open reading frames of variants 1 and 5 are located in a single exon but may use different translation initiation sites after splicing. Variant 5 is the shortest and shares its sequence with the other variants. Variants 1 and 2 share all sequences except the N-terminus with different nine and five amino acids, respectively. Variants 3 and 4 have much longer N-termini with an extra exon each (Fig 1B). Expression of all five CXCR4 variants was determined by RT-PCR with variant-specific primer pairs in several cell lines. Variants 3 and 4 were distinguished by product size with the same primer pair because they share most of each exon. PCR products of all variants were detected in HEK293, HeLa, THP-1, and U937 cells, and variants 1 and 2 were dominant in these cells. Only variant 2 was detected in SKOV3, A673, and A549 cells with very low levels detected in SKOV3 and A549 cells. Unlike other cells, NCI-H460 cells predominantly expressed variant 1 (Fig 1C). These mRNA expression patterns suggest that alternative splicing of transcribed RNA may occur with different efficiency in different cell types.

### N-terminal sequence differences affect expression of CXCR4 variants

**Fig 2:**
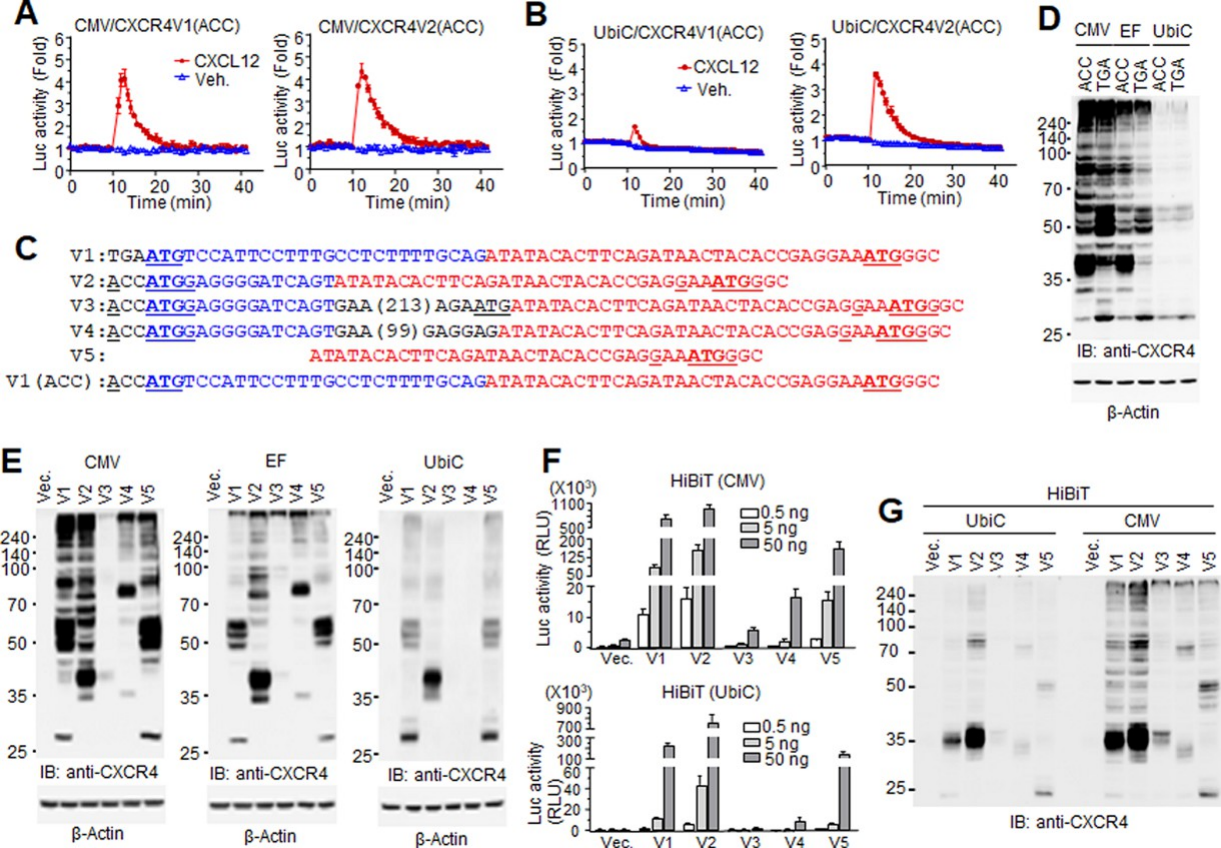
CXCR4 splicing variant-mediated calcium response and expression pattern. (A, B) Calcium responses mediated by CXCR4 variant 1 driven by different promoters. Variant 1 and 2 sequences were inserted into pcDNA3.1 (CMV promoter) or pUbiC3.1 (ubiquitin C promoter). ‘ACC’ nucleotides were added in front of ‘ATG’ to enhance expression efficiency. The variants were expressed with NanoBiT-based calcium probes in HEK293 cells, and luciferase activity was measured by luminometer. CXCL12-stimulated luciferase activity (Ca^2+^ influx) was similar in cells expressing CMV-driven variants 1 and 2 (A), whereas luciferase activity was significantly lower in cells expressing UbiC-driven variant 1. (C) Nucleotides around the translation start codon of CXCR4 variants. (D) Promoter-dependent expression of ‘ACC’ variant 1 or ‘TGA’ variant 1 was analyzed by western blotting. (C) Promoter-dependent expression of CXCR4 variants. Variant 1 is the ‘TGA’ form unless otherwise stated. Each variant was inserted in expression vectors containing the CMV, EF, or UbiC promoter. (F) Cell surface expression of CXCR4 variants. The variants were constructed as N-terminal HiBiT-tagged forms in vectors containing the CMV or UbiC promoters. Cells transfected with different amounts of vector were applied to the HiBiT assay. (G) Cells expressing HiBiT-tagged variants were lysed and applied to western blotting with anti-CXCR4 antibody.

Analysis of intracellular Ca^2+^ increase is a general and reliable readout for GPCR activation [28]. Most experiments for this purpose are performed with exogenous receptors driven by strong promoters, such as cytomegalovirus (CMV). This system is useful to characterize the biochemical properties of certain GPCRs and to identify their agonists and antagonists; however, overexpressed proteins may elicit exaggerated and/or biased signals with high basal activities, which do not reflect the physiological responses mediated by the receptors. To evaluate the CXCL12-stimulated Ca^2+^ response, we compared two different expression vectors: pcDNA3.1 containing the CMV promoter and pUbiC3.1 containing the ubiquitin C (UbiC) promoter. pUbiC3.1 was constructed by replacing the CMV promoter with the relatively weak ubiquitin C promoter in pcDNA3.1 [12]. The cDNA of each CXCR4 variant was inserted via the same restriction enzyme sites in both vectors. Vectors containing the CXCR4 variants were introduced along with plasmids for the NanoBiT-based calcium assay to HEK293 cells harboring a Gα_qi_ chimera, which converts the Gα_i_ signal to the Gα_q_ pathway for Ca^2+^ influx [29]. The mechanism underlying the NanoBiT assay is Ca^2+^-dependent assembly of NanoLuc fragments, which generate luciferase activity, as described in the Materials and Methods. As chemokine receptors may induce weak Ca^2+^ responses through Gα_i_ and G_βγ_, the Gα_qi_ chimera makes it possible to observe activation of the Gα_i_ pathway through Ca^2+^ influx [30]. Upon CXCL12 stimulation (100 ng/ml), significant increases in luciferase activity were observed in cells expressing variants 1 and 2 driven by the CMV promoter (Fig 2A), which is consistent with previous reports [22, 25]. However, when the variants were expressed by the UbiC promoter, only variant 2 mediated a significant increase in luciferase activity (Fig 2B).

Usually, the nucleotides ‘ACC’ are inserted before the translation start codon ‘ATG’ in expression vectors to facilitate the expression of the gene, following Kozak’s rule. However, addition of ‘ACC’ in the UbiC expression vector did not induce expression of CXCR4 variant 1. Thus, we examined the cDNA sequences of all variants to identify those possibly affecting variant expression. Interestingly, the sequence around the potential translation start codon of variant 1 differed from that of the other variants. The sequence ‘TGAATGT’ in the potential translation start site of variant 1, designated by the genome database, does not follow Kozak’s rule (A/G-N-N-ATG-G). In contrast, variants 2, 3, and 4 share an exon containing a translation initiation codon, which matches the consensus sequence (ACC-ATG-GAG) of Kozak’s rule exactly. Variant 5 seems to be translated from the consensus sequence shared by all variants (GAA-ATG-GGC) (Fig 2C). To determine the effect of Kozak’s rule and promoter efficiency on gene expression, CXCR4 variant 1 genes containing either ‘ACC’ or ‘TGA’ in front of ‘ATG’ were inserted into the plasmids harboring different promoters, and their expression was examined by western blotting with CXCR4 antibodies. As shown in Fig 2D, protein expression was dependent on promoter efficiency (CMV>EF (elongation factor promoter)>>UbiC) and was detected as multiple bands together with bands stuck around the wells of the gel, which is a typical pattern of artificially expressed GPCRs in western blotting. Interestingly, strong promoters, such as CMV and EF, resulted in different main bands at 40 kDa and 50–60 kDa, respectively, depending on the upstream sequence (‘ACC’ or ‘TGA’). However, both proteins driven by UbiC were detected at 50–60 kDa as faint bands. When the different promoter-driven variants, including ‘TGA’ variant 1, were expressed in HEK293 cells and subjected to western blotting, their expression varied in terms of efficiency and size (Fig 2E). ‘TGA’ variant 1 had a similar expression pattern to variant 5 regardless of promoter, indicating that ‘TGA’ variant 1 shares the variant 5 ‘ATG’ site instead of the ‘ATG’ following the added ‘TGA’. The main bands of variant 2 were detected around 40 kDa, similar to ‘ACC’ variant 1. Variant 3 was detected as faint multi-bands in all blots despite containing a Kozak sequence, implying that the long N-terminal sequence of variant 3 may not be efficiently translated or may cause protein instability. Variant 4 was detected around 80 kDa under the CMV and EF promoters but not under the UbiC promoter.

To assess membrane expression of the CXCR4 variants, each variant was expressed in an N-terminal HiBiT-tagged form. HiBiT-dependent luciferase activity increased depending on the amount of plasmid introduced, which was comparable to the observed western blotting results. Interestingly, variant 1 showed different activity under the control of the CMV and UbiC promoters. CMV-driven expression led to similar activity between variants 1 and 2. However, variant 1 activity was significantly lower than variant 2 in UbiC-driven expression even though translation was initiated from a start codon in the N-terminal HiBiT-tag (Fig 2F), implying that the unique sequence of variant 1 may affect CXCR4 expression or membrane localization. To further assess expression of the HiBiT forms of the variants, western blotting was performed with anti-CXCR4 antibodies. HiBiT-variant 1 driven by both promoters localized at the same size as variant 2 but not variant 5. As N-terminal HiBiT-variant 1 expression and its protein level were substantially lower than HiBiT-variant 2, it is likely that the N-terminus of variant 1 negatively affects protein expression (Fig 2G).

### CXCR4 variants differentially mediate cellular responses to CXCL12

**Fig 3:**
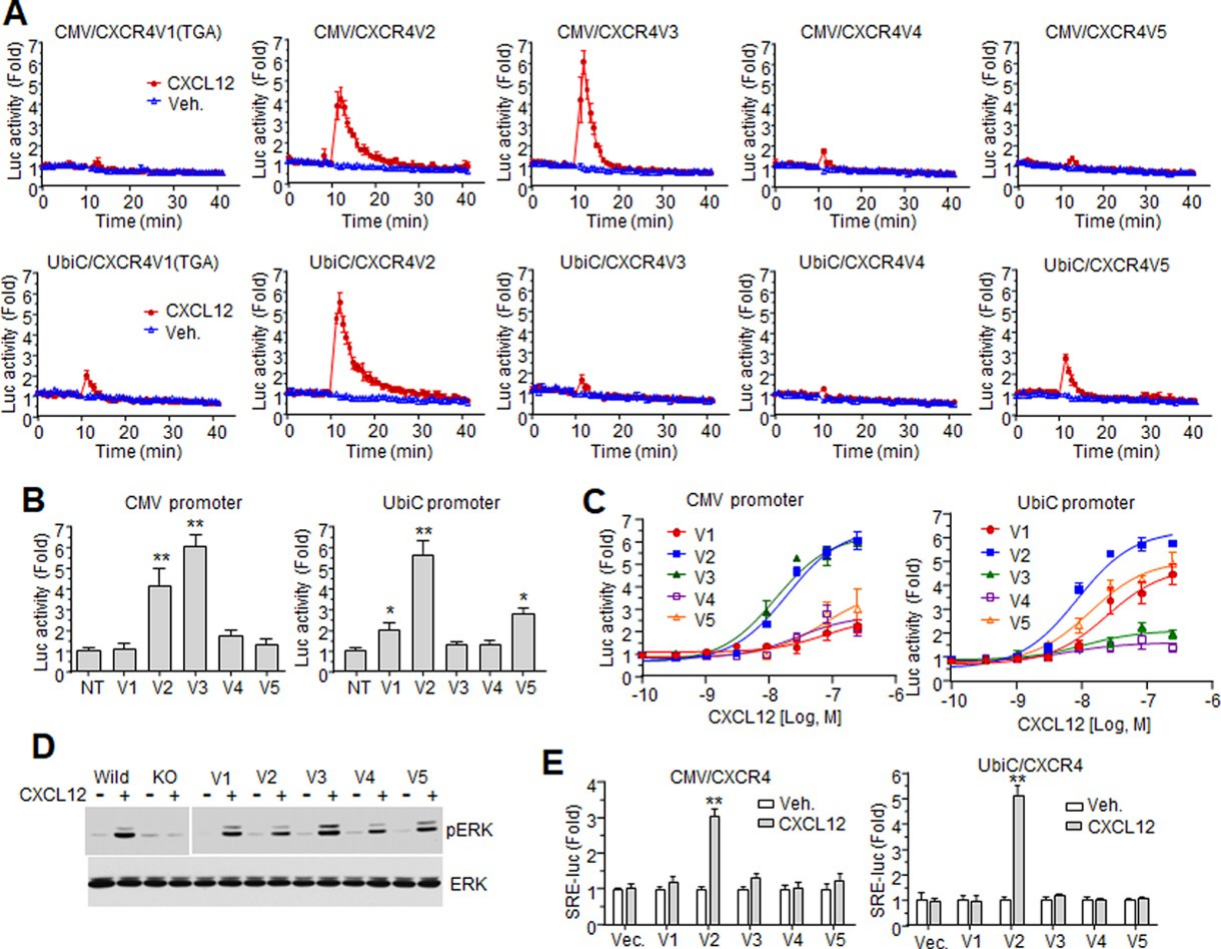
Analysis of variant-mediated cellular response to CXCL12. (A) Cells expressing NanoBiT-based calcium probes and each variant controlled by either the CMV or UbiC promoter were treated with 100 ng/ml CXCL12, and luciferase activity was measured in real-time. (B) Maximum luciferase activity after CXCL12 treatment in cells expressing each variant driven by different promoters. **: p<0.01, *: p<0.05 (C) CXCL12 dose dependency of maximum luciferase activity. (D) CXCL12-stimulated ERK phosphorylation in cells reconstituted with each variant. Cells were treated with 100 ng/ml CXCL12 and applied to western blotting. Samples were loaded on two gels and transferred each to different nitrocellulose membranes. After all procedures, the membranes were exposed at the same time. The cropped images were presented on the same panel. (E) SRE-dependent luciferase gene expression stimulated by CXCL12 in cells expressing each variant driven by the CMV or UbiC promoter. **: p<0.01.

The effects of CXCR4 variants on CXCL12-mediated cellular responses were determined by analyzing signaling events. In most experiments, variant 1 containing ‘TGA’ was used unless otherwise stated. The Ca^2+^ influx triggered by 100 ng/ml CXCL12 was measured by NanoBiT calcium probes in HEK293 cells expressing chimeric G protein Gα_qi_. When CXCR4 variants were expressed by the CMV promoter, significant increases in luciferase activity were observed in cells expressing variants 2 and 3, indicating that both variants mediate Ca^2+^ influx efficiently. The signals generated by variant 3 were strong but short-lived in comparison to those generated by variant 2 (see tails of red lines). Luciferase activity was scarcely increased by CXCL12 in cells expressing the other CXCR4 variants (Fig 3A, upper graphs). The calcium responses in cells expressing each variant driven by the UbiC promoter were different from those driven by the CMV promoter. Variant 2 mediated strong luciferase activity in both expression conditions. However, the activities were negligibly increased in cells expressing UbiC-driven variant 3. In this expression condition, variants 1 and 5 mediated slight increases in luciferase activity. Variant 4 did not mediate any luciferase activity in either condition (Fig 3A). The variant-mediated maximum luciferase activities in both expression conditions are shown in Fig 3B. Dose-response curves of CXCL12 in cells expressing each variant driven by the CMV or UbiC promoter revealed different efficiencies of CXCR4 on calcium responses in an expression condition-dependent manner, which was similar to the responses to single point treatment (CMV: V2 = V3>>V1 = V4 = V5, UbiC: V2>V5≅V1>V3≅V4) (Fig 3C).

Phosphorylation of extracellular signal-regulated kinase (ERK), which is a representative signaling event in GPCR activation, was stimulated by CXCL12 in HEK293 cells, implying that the transcripts of CXCR4 variants were translated to functional receptors. However, chemokine-stimulated Ca^2+^ influx was not detected even though the NanoBiT-based Ca^2+^ assay is very sensitive. Regardless of mRNA expression level, the endogenous CXCR4 protein level seems to be sufficient to induce ERK phosphorylation but not to trigger intracellular Ca^2+^ increase, which is consistent with cellular response to monocyte chemoattractant protein-1 (MCP-1) activation of endogenous CCR2 in HEK293 cells [31]. In other words, ERK phosphorylation is the most sensitive response to GPCR activation. To determine the effect of each CXCR4 variant on ERK phosphorylation, CXCR4 was deleted by CRISPR-Cas9 using a specific target sequence, and the expression of each variant was reconstituted by transfection with plasmids containing each variant. CXCL12-stimulated ERK phosphorylation was not detected in the knock-out (KO) cells. Interestingly, reconstitution of each variant restored ERK phosphorylation after CXCL12 stimulation (Fig 3D). Unlike the Ca^2+^ response, variant 2-mediated phosphorylation was lower than other variants. These results suggest that the ERK phosphorylation mediated by each variant may be induced with different efficiency through unknown mechanisms regardless of CXCR4 expression level and therefore may be an inappropriate measure of variant signaling efficiency (Fig 3D).

Intracellular Ca^2+^ influx and ERK phosphorylation are a prerequisite for target gene expression mediated by the serum response element (SRE) [32, 33]. Therefore, we investigated the ability of each variant to activate the SRE as another measure of signaling efficiency of the variants. HEK293-Gα_qi_ cells were transfected with plasmids for each CXCR4 variant as well as SRE-luc. Cells were then treated with 100 ng/ml CXCL12, and luciferase activity was measured. Under control of either the CMV or UbiC promoter, only variant 2 mediated significant luciferase activity after chemokine stimulation. This contrasts our earlier experiments where other CXCR4 variants mediated Ca^2+^ influx or ERK phosphorylation under certain expression conditions. As gene expression is a downstream event, reporter gene assays may be less sensitive to receptor activation; however, variant 2 was able to induce downstream SRE activity (Fig 3E).

### CXCL12-dependent interaction of CXCR4 variants with heterotrimeric G proteins and β-arrestin2

**Fig 4:**
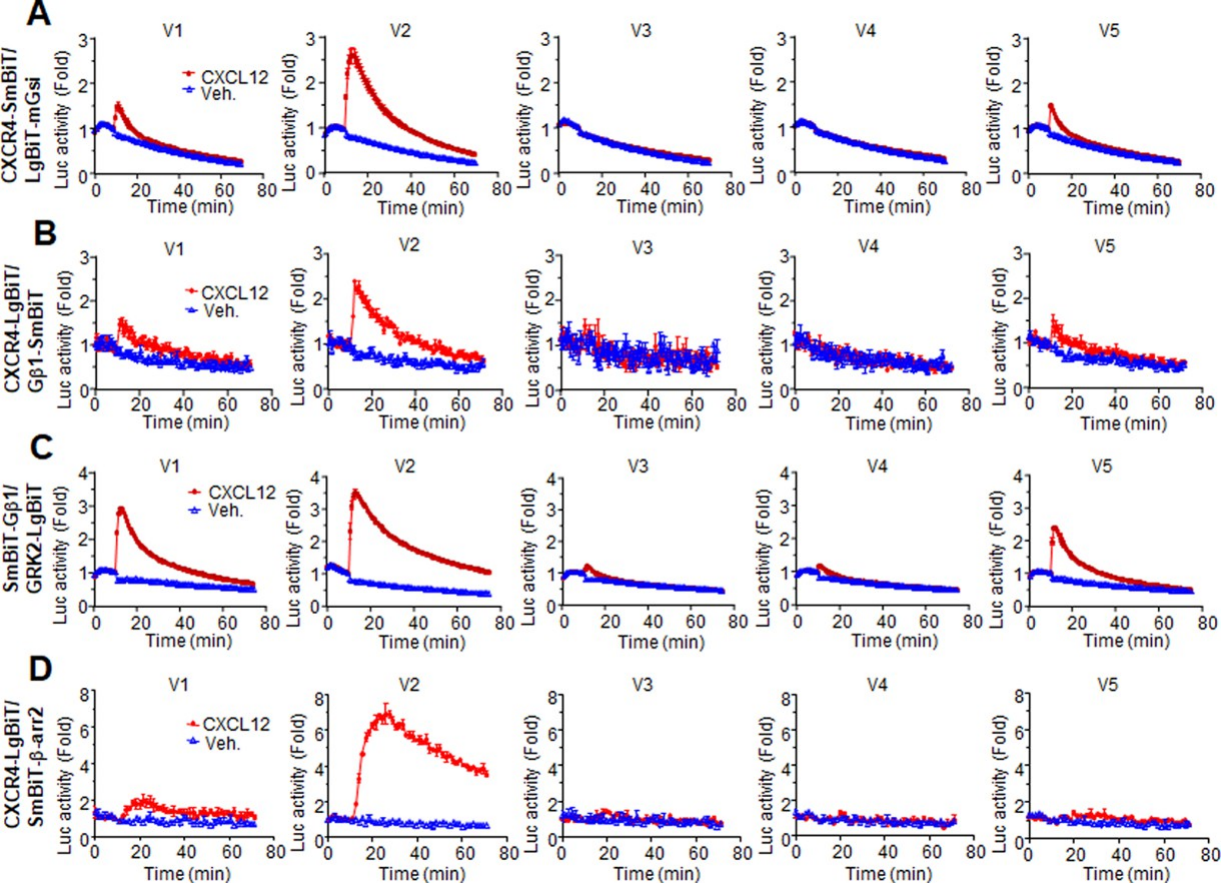
CXCL12-stimulated interaction of each CXCR4 variant and downstream signaling molecules. (A) HEK293 cells expressing LgBiT-Gsi and each C-terminal SmBiT-tagged CXCR4 variant were treated with 100 ng/ml CXCL12, and luciferase activity was measured. (B) CXCL12-dependent interaction of Gβ1-SmBiT and each C-terminal LgBiT-tagged CXCR4 variant. (C) Cells expressing each variant with SmBiT-Gβ1 and GRK2-LgBiT were treated with 100 ng/ml CXCL12, and luciferase activity was measured. (D) CXCL12-dependent interaction of SmBiT-β-arrestin and each C-terminal LgBiT-tagged CXCR4 variant.

In general, chemokine receptors are known to mediate signals through Gα_i/o_ and Gα_12/13_ upon ligand binding. However, physical interaction of GPCRs and G proteins is difficult to determine using classic biochemical techniques, such as immunoprecipitation and immunocytochemistry, due to the transient nature of the interaction and to the complex structure of GPCRs with multiple transmembrane domains. Recently, Nehme, R., *et al*. and Wan, Q., *et al*. engineered chimeric Gα proteins called mini G proteins, which have an active conformation, to bind ligand-occupied GPCRs [34, 35]. To explore the mode of CXCR4-mediated G protein activation, the biophysical interaction of CXCR4 with the mini G proteins, such as mini Gs, Gsi, Gsq, G12, and G16, was assessed by structural complementation assay using NanoBiT technology. The NanoLuc fragments, LgBiT and SmBiT, were fused to mini G proteins at the N-terminus or C-terminus and the C-terminus of CXCR4. All combinations of LgBiT- or SmBiT-fused proteins were applied to the NanoBiT assay under CXCL12 stimulation. Expression of all constructs was driven by the ubiquitin C promoter. Finally, the best signals were observed in combination with CXCR4-SmBiT and LgBiT-mini G proteins, as shown in other chemokine signaling studies [31]. HEK293 cells expressing CXCR4 variant 2-SmBiT and each LgBiT-mini G protein were stimulated with CXCL12, and luciferase activity was measured in real-time. Upon CXCL12 treatment, luciferase activity increased in cells expressing CXCR4 variant 2 (V2)-SmBiT and LgBiT-mini Gsi, whereas no signal change was observed with the combination of the receptor and the other mini G proteins, indicating that CXCR4 V2 activated the Gα_i_ pathway (S1 Fig). Gα_12/13_ and Gα_16_ are activated by chemokine receptors [36–38]. The mini G protein forms of Gα_12/13_ and Gα_16_ did not increase luciferase activity when combined with the receptor, confirming that these mini G proteins may be insufficient to determine GPCR interaction with their natural forms, as described in previous reports [34]. To assess the efficiency of CXCR4 variants on Gα_i_ protein signaling, the C-terminal SmBiT form of each variant and LgBiT-mini Gsi were expressed in HEK293 cells. CXCL12-dependent luciferase activities were strong in the presence of variant 2 and were relatively weak in the presence of variants 1 or 5. There was no change in activity in cells expressing variants 3 and 4 (Fig 4A). Ligand-bound GPCRs induce the GTP form of Gα and separation of Gβγ from the Gα-GTP. Free Gβγ initiates the first step of receptor internalization by recruiting G-protein coupled receptor kinases (GRKs) to activated receptors [39]. Then, GRK phosphorylation of the receptors creates a β-arrestin binding region [39, 40]. To determine the effect of the variants on this process, NanoBiT constructs of the components were established. Luciferase activities from each C-terminal LgBiT form of CXCR4 variant and Gβ1-SmBiT were increased by CXCL12 stimulation with different intensity (V2>>V1 = V5) (Fig 4A and 4B). The luciferase activities of SmBiT-Gβ1 and GRK2-LgBiT were increased with different efficiency (V2>V1≥V5>>V3≅V4) (Fig 4C). β-arrestin recruitment by the variants was demonstrated with C-terminal LgBiT of each receptor and SmBiT-β-arrestin2. CXCL12 stimulation increased luciferase activity with different efficiency, depending on the variant (V2>>V1>V5) (Fig 4D). The binding assays using NanoBiT technology revealed that variant 2 is likely the dominant CXCL12 signal transducer. Variants 1 and 5 produced the same proteins and transduced signals less efficiently with some leakage. As variants 3 and 4 were expressed at very low levels, the corresponding NanoBiT constructs could not induce luciferase activity with their binding partners.

### CXCL12-stimulated internalization of CXCR4 variants

**Fig 5:**
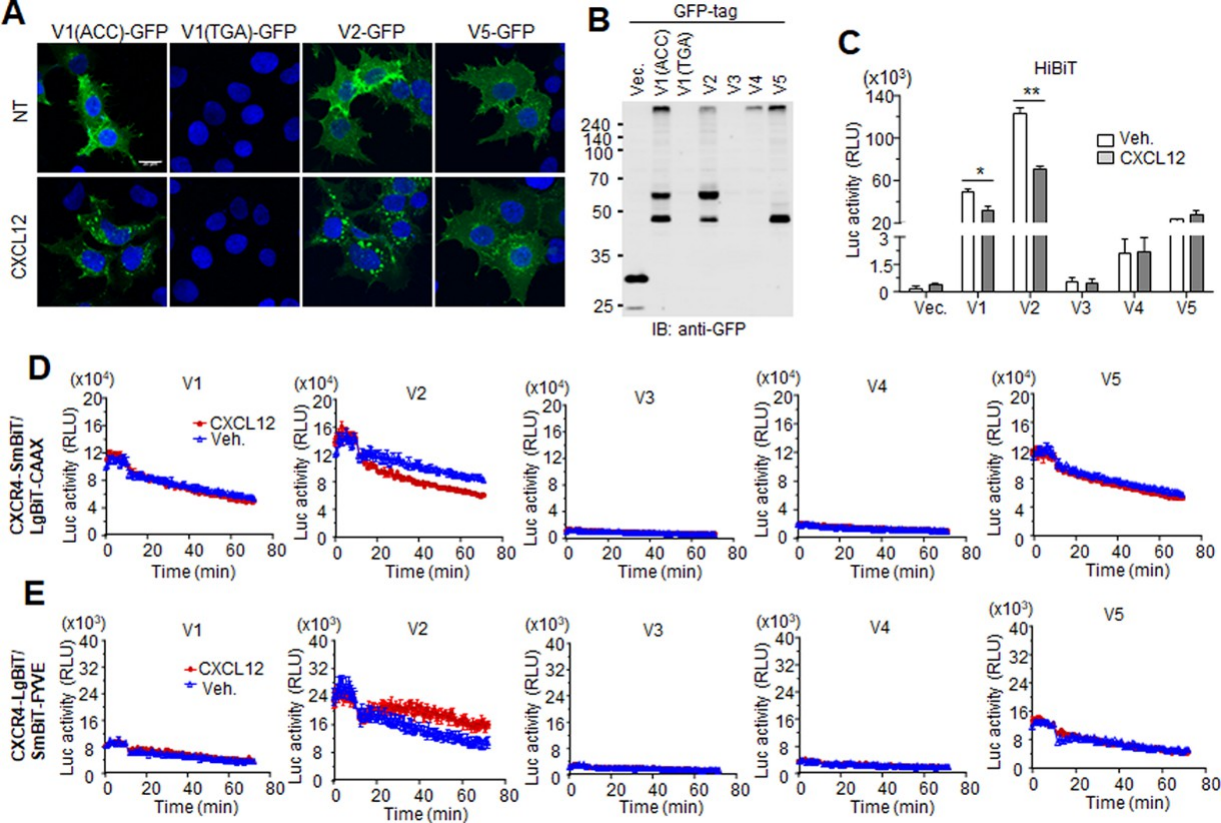
Internalization of CXCR4 variants. (A) Cells expressing C-terminal GFP-tagged variants were treated with 100 ng/ml CXCL12 for 30 min and fixed with 4% paraformaldehyde. GFP signal was observed under confocal microscope. Images of variants 3 and 4 were not included, because they did not show any signal. (B) Western blotting for GFP-tagged variants. Cells expressing each GFP-tagged variant were lysed and used for western blotting with anti-GFP antibody. Comparison of expression of ‘ACC’ and ‘TGA’ variant 1. (C) HiBiT assay. Cells expressing UbiC-driven HiBiT-tagged variants were treated with 100 ng/ml CXCL12 for 30 min, and luciferase activity was measured. **: p<0.01, *: p<0.05 (D) CXCL12-stimulated membrane dissociation of CXCR4 variants. Cells expressing LgBiT-CAAX and each C-terminal SmBiT-tagged variant were treated with CXCL12, and luciferase activity was measured. (E) CXCL12-stimulated endocytosis of CXCR4 variants. Cells expressing SmBiT-FYVE and each C-terminal LgBiT-tagged variant were treated with CXCL12, and luciferase activity was measured.

To assess ligand-dependent internalization, all variants were constructed in pEGFP-N1 (C-terminal EGFP tag) and expressed in HEK293 cells. After fixation with 4% paraformaldehyde and DNA staining with Hoechst 33342, expression of the variants was observed under confocal microscope. The EGFP signals of ‘ACC’ variant 1, variant 2, and variant 5 were detected at the plasma membrane, and the signal intensity of variant 5 was slightly weaker than the others. However, it was hard to detect the fluorescence of ‘TGA’ variant 1, variant 3, and variant 4 due to their low expression level. Interestingly, the EGFP-tagged form of ‘TGA’ variant 1 did not seem to share a start codon with variant 5 for an undefined reason, even though intact forms of both variants showed the same expression pattern in western blotting (Fig 2E). CXCL12 induced cytosolic clustering of EGFP signals in cells expressing ‘ACC’ variant 1 or variant 2, whereas EGFP signals of variant 5 were not clustered, indicating that variant 5 was not internalized by the chemokine (Fig 5A). Expression of the GFP-tagged variant forms was also confirmed by western blotting with anti-GFP antibodies. ‘ACC’ variant 1 and variant 2 showed similar migration patterns, whereas ‘TGA’ variant 1 was not detected, which was consistent with the imaging results (Fig 5B). The internalization was confirmed by HiBiT assay as shown in Fig 5C. Because the HiBiT sequence was tagged to the N-terminal region of the receptors, variant 1 was expressed well at the cell surface like ‘ACC’ variant 1 but with lower efficiency from the UbiC promoter (see Fig 2F). The luciferase activities from the HiBiT constructs of both variants 1 and 2 were significantly reduced by CXCL12 stimulation, but the luciferase activity of variant 5 was not altered (Fig 5C). The CAAX motif in membrane-attached proteins enables plasma membrane localization through isoprenylation at the cysteine residue and proteolysis [41]. Thus, LgBiT-CAAX can localize to the intracellular surface of the plasma membrane and bind to C-terminal SmBiT-tagged GPCRs localized on the membrane. The luciferase activities resulting from the NanoBiT constructs showed that variant 2 was expressed in the plasma membrane and was dissociated from the cell surface after CXCL12 stimulation, indicating internalization of the variant. The luciferase activity of variants 1 and 5 was relatively high but did not change in response to the chemokine when compared to untreated groups. This result revealed that both variants in the SmBiT construct are likely to share the same translation start codon and are not internalized following extracellular stimulation (Fig 5D). This finding was confirmed using another NanoBiT assay with LgBiT forms of the variants and SmBiT-FYVE, which is an early endosomal marker. Using this assay, the luciferase activity of variant 2 increased after CXCL12 stimulation, indicating translocation of the receptor to the endosome (Fig 5E).

### Complex formation of CXCR4 variants

**Fig 6:**
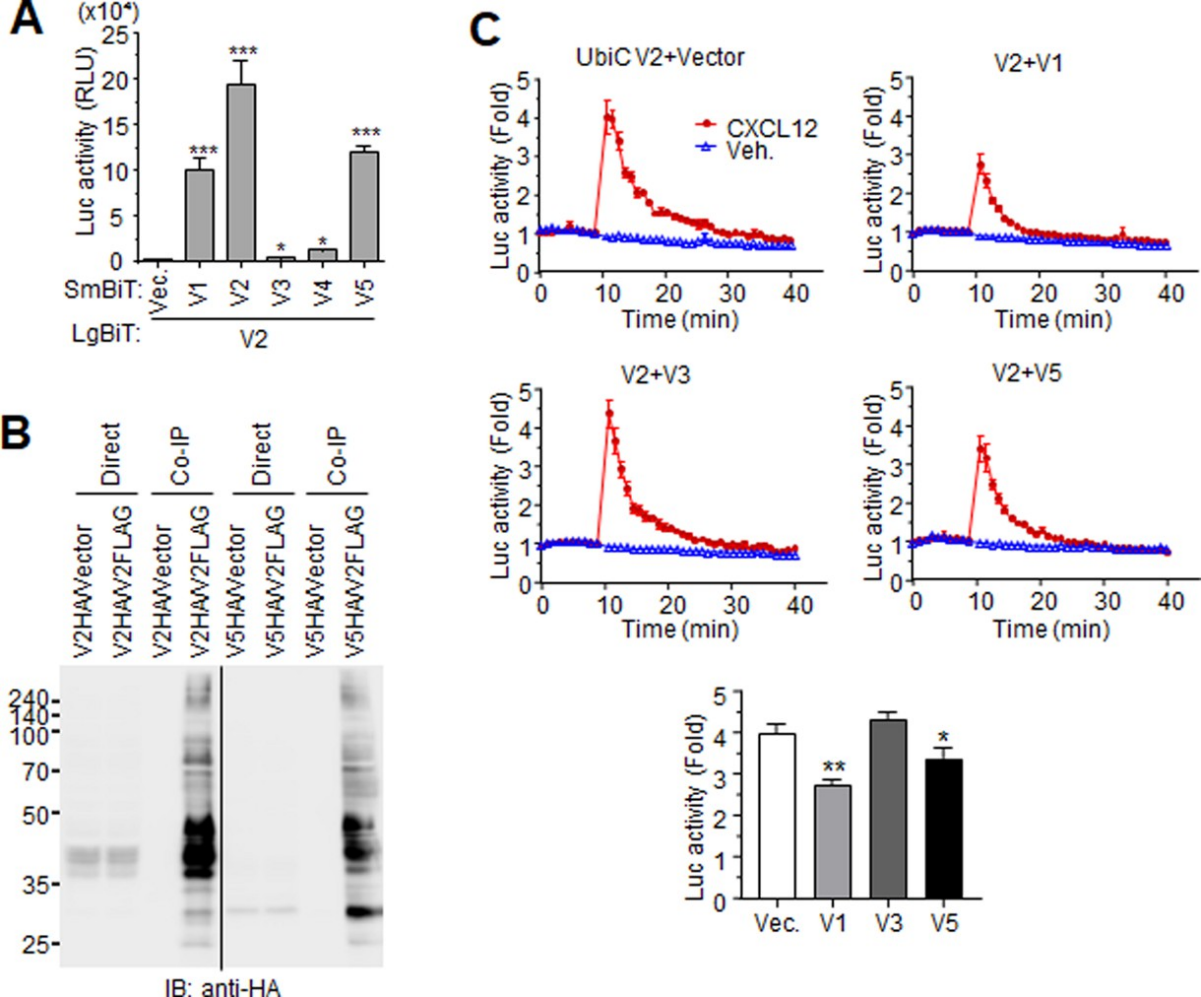
Dimerization of CXCR4 variants. (A) Cells expressing variant 2-LgBiT with each SmBiT-tagged variant were applied to the NanoBiT assay. After 24 hr, luciferase activity was measured. (B) Cells expressing variant 2-FLAG with either HA-tagged variant 2 or variant 5 were lysed and applied to immunoprecipitation with anti-FLAG antibody and subsequent western blotting with anti-HA antibody. Fragments of the same original image were spliced together to remove irrelevant lanes. (C) Effect of the other CXCR4 variants on variant 2-mediated Ca^2+^ influx. Cells expressing UbiC-driven variant 2 with indicated variant and NanoBiT-based calcium probes were treated with 100 ng/ml CXCL12, and then luciferase activity was measured. Lower graph: peaks of the CXCL12-stimulated luciferase activity. **: p<0.01, *: p<0.05.

Like many other GPCRs, CXCR4 may form homodimers or oligomers at the cell surface to enhance its membrane expression or to efficiently transduce signaling [42]. C-terminally LgBiT-tagged variant 2 (V2-LgBiT) was co-expressed with each C-terminally SmBiT-tagged variant in HEK293 cells, and luciferase activity of the NanoBiT constructs was measured. As shown in Fig 6A, the luciferase activity of every combination was higher than that of V2-LgBiT alone. SmBiT forms of variants 3 and 4 slightly increased luciferase activity due to low expression levels. To further assess the complex formation of the variants, HEK293 cells expressing C-terminally FLAG-tagged variant 2 (V2-FLAG) with either HA-tagged variant 2 (V2-HA) or variant 5 (V5-HA) were lysed and immunoprecipitated with anti-FLAG agarose. Western blotting was then performed with anti-HA antibodies. Both V2-HA and V5-HA were detected in the precipitates, indicating that variant 2 dimerized with itself as well as with variant 5. In total cell lysates, the anti-HA signal was indistinct compared with previous blots using cell extracts (see Fig 2E) due to the mild extraction conditions necessary for immunoprecipitation (Fig 6B). Since transcripts of multiple variants were detected in cells (Fig 1C), the expression and activity of each variant may be influenced by the others. To explore the effects of the other variants on variant 2-mediated calcium response to CXCL12, HEK293 cells were transfected with UbiC-driven variant 2, each of the other variants, and NanoBiT-based calcium probes. The resulting chemokine-stimulated luciferase activity was measured. Compared to variant 2 alone, co-expression of variant 2 and either variants 1 or 5 reduced the CXCL12-stimulated induction of enzyme activity, implying that variants 1 and 5 negatively regulate the activity of variant 2. Expression of variant 3 had no effect on variant 2-mediated calcium response to CXCL12, possibly due to its low expression (Fig 6C).

### The effects of CXCR4 variants on proliferation and migration of HeLa cells

**Fig 7:**
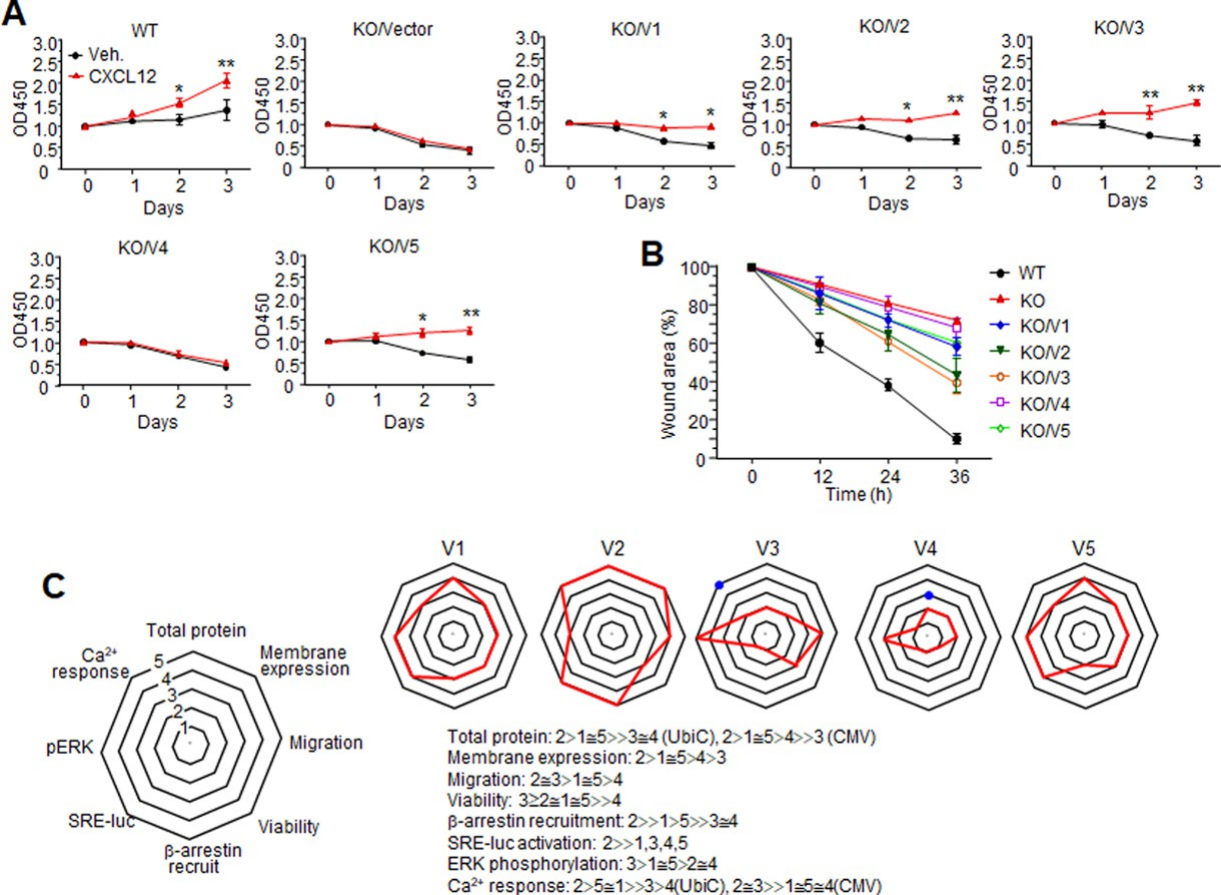
Cellular responses mediated by CXCR4 variants. (A) Growth assay. Parental HeLa cells and CXCR4 KO cells reconstituted with each CXCR4 variant were seeded into 96-well plates with serum-free DMEM with or without 100 ng/ml CXCL12. The CCK-8 assay was performed with different plates on each day. Values are means ± SD. **: p<0.01, *: p<0.05 (B) Wound healing assay. Wounded areas were measured at different time points. The graph shows the remaining area by percentage. Values are means ± SDs. (C) Properties of each variant in terms of protein expression, CXCL12-stimulated signaling, and CXCL12-stimulated cellular responses. All values were graded on a 5-point scale. Red indicates the grade of outcome values from individual analysis of each variant. Blue dots indicate the results of CMV promoter-driven expression.

To assess the effect of each variant on cellular responses, we deleted CXCR4 using CRISPR-Cas9 in HeLa cells, which express all CXCR4 splicing variants. Then, each variant was reconstituted in the KO cells by lentivirus infection. In serum-free conditions, cells were treated with 100 ng/ml CXCL12 every day, and proliferation was measured by CCK-8 assay. Parental HeLa cells did not grow well in the absence of serum, and their growth was significantly enhanced in the presence of CXCL12. In contrast, growth of CXCR4 KO cells was not observed without serum or only with CXCL12, but their viability looked decreased. Variant 4 had no effect on cell viability under any condition, but expression of variants 1, 2, 3, and 5 restored cell viability and slightly enhanced cell growth in response to CXCL12, suggesting that some variants may contribute to cell viability (Fig 7A). Notably, the growth rate of all cell groups used in this experiment was comparable to parental HeLa cells in the presence of serum and was not affected by CXCL12, implying that CXCL12 and CXCR4 are not critical for cell growth but may play a role in cell survival (S2 Fig).

Although there are some reports of chemotactic migration toward CXCL12 [43, 44], very few HeLa cells migrated through transwells in chemotaxis assays, implying that neither endogenous CXCR4 nor reconstituted variants were enough to induce motility of HeLa cells. An alternative explanation could be that cells were not motile enough to penetrate the transwell. To further assess the effect of CXCR4 variants on the motility of HeLa cells, we used a wound-healing assay. Parental HeLa cells almost closed the scratched area in the presence of serum and 100 ng/ml CXCL12 after 36 hr, but the scratched area remained wide in KO cells and variant 4 reconstituted cells. The scratched area was significantly decreased in cells expressing variants 2 and 3 and was slightly reduced in cells expressing variants 1 and 5 (Figs 7B and S3).

We compared and summarized the properties of all CXCR4 variants in terms of protein expression, CXCL12-stimulated signaling, and CXCL12-stimulated cellular response (Fig 7C). The relative values from all results were graded on a 5-point scale. Variant 2 was the most promising receptor for CXCL12. Variants 1 and 5 were very similar and shared the same translation start codon even though the variant 1 transcript was longer. The molecular properties of variant 3 were difficult to characterize due to low expression, but the significant cellular responses mediated by the protein imply that variant 3 may have other functions. Variant 4 was not a functional chemokine receptor, since ERK phosphorylation, which was the most sensitive measure used in this study, was the only positive result achieved with variant 4.

## Discussion

Many gene transcripts are alternatively spliced after transcription. Alternative splicing is a critical step that enables functional diversity of genes, allowing cells and organisms to have unique responses to various stimuli with a finite number of genes. Although most GPCRs are translated from a single exon, some chemokine receptor genes are expressed in various forms, containing different amino acids and lengths in the N-terminal region. As the N-terminal region is not structurally defined but provides the first binding site of cognate chemokines, different amino acid compositions and lengths may affect ligand binding affinity and specificity. Furthermore, different N-terminal sequences may affect cell surface expression of the 7-transmembrane proteins due to the lack of a signal peptide necessary the expression of most membrane proteins and secretory proteins, thereby the N-terminal sequences of each CXCR4 variant may affect their expression. Also, the amount of each transcript may confer the protein expression level. RT-PCR revealed that cells are likely to express several splicing variants with different expression efficiency. Indeed, the mRNA level of variant 2 was generally higher than the others, and the mRNA level of variant 1 was higher in some cell lines. These expression patterns support the use of variant 2 as the primary variant to characterize CXCR4 and to conduct inhibitor screening for the receptor in previous studies [22, 45]. Although the expression of the other CXCR4 variants was relatively low, they may still play a role in cellular responses.

For functional analysis and drug development, the expression vectors usually contain strong promoters, such as CMV and EF, to enhance gene expression. Although this expression system is useful for analysis of cellular responses related to gene expression, there is potential for biased responses to be caused by overexpression, which may make it difficult to determine the physiological roles of the gene products. Overexpression of GPCRs increases the active form of the receptors by altering the equilibrium between the inactive and active states, which elicits cellular activation without ligand stimulation [46, 47]. Our results showed that strong promoter-driven receptor expression generally led to high basal receptor activity and exaggerated cellular responses. However, when receptor expression was driven by a weak promoter, low basal activity was observed along with low levels of the protein and enhanced ligand-dependent activation. Although any exogenous expression is different from endogenous expression, our functional assay results using a weak promoter may better reflect the physiological activity of the receptors. Variant 1 has been reported to mediate CXCL12 stimulation with an efficiency comparable to variant 2 [48]. However, we found that variant 1 hardly elicited cellular responses to CXCL12 when its expression was controlled by UbiC promoter. This discordance may be due to the potential start codon previously designated, which was not relevant to the Kozak sequence [25]. Use of a strong promoter and artificial nucleotides around ‘ATG’ in expression vectors may have affected the expression of variant 1 in previous studies. Nucleotide analysis and western blot data for each variant revealed that variant 1 and variant 5 transcripts likely produce the same proteins through common ‘ATG’ sites identified in all variants. This observation was confirmed in our functional analyses and is evident in the summary of variant properties in Fig 7C. However, neither C-terminal GFP- or HA-tagged form, the proteins were not detected by western blotting or confocal microscopy, whereas both intact proteins and NanoBiT constructs of the variant were expressed at levels sufficient to analyze their function. In contrast, variant 5 was efficiently expressed in all vector systems. The different expression patterns of variants 1 and 5 in different vector systems despite their protein similarity may be explained by the untranslated sequences in variant 1, the role of which is difficult to determine. Regardless of the different expression patterns of epitope-tagged variant 1 in artificial vector systems, endogenous variants 1 and 5 are expected to be translated to the same proteins, since their intact forms were expressed with similar efficiency.

The promoter effect on gene expression was obvious in variant 3. Total protein and cell surface expression of variant 3 were low compared to the other variants in all vectors. Interestingly, CMV-driven variant 3 efficiently mediated CXCL12-dependent Ca^2+^ response with a shorter duration than that mediated by variant 2, whereas this response was negligible in cells expressing variant 3 driven by UbiC. Based on these results, the N-terminal region of variant 3 is expected to form a favorable binding site on the cell surface for the ligand even though it plays a negative role in the membrane expression of the receptor. In the case of variant 4, strong promoters induced detectable protein expression in western blotting and on the cell surface, but the proteins did not transduce a Ca^2+^ response, indicating that the N-terminal region forms an unfavorable site for ligand recognition.

Chemokines are involved in directional migration and mild proliferation of cells expressing their receptors. HeLa cells may expressed all CXCR4 variants, and their growth and migration activity were enhanced by CXCL12. However, reconstitution of each variant in CXCR4 KO cells revealed that cellular responses mediated by individual variants were not as strong as those in parental cells, even though variants 1, 2, 3, and 5 modestly contributed to cell survival and migration, implying that splicing variants may interact each other to influence cellular responses. Indeed, complex formation of CXCR4 variants was confirmed by immunoprecipitation and NanoBiT assays. CXCL12-stimulated Ca^2+^ influx was slightly reduced in cells co-expressing variant 2 and either variants 1 or 5 compared with cells expressing variant 2 alone, indicating that variants 1 and 5, which may generate the same protein, have a negative effect on variant 2 activity. Taken together, the splicing variants of CXCR4 are likely to affect each other in both positive and negative ways to elicit cellular responses in various intra- and extracellular environments.

In this study, we demonstrated the molecular and functional properties of CXCR4 splicing variants using various analytical approaches. NanoBiT-based assays were a powerful method to determine the molecular interactions and behaviors of signaling components, although the application of NanoBiT-based assays was limited by protein expression level. Expression of the receptors and cellular responses to CXCL12 suggested that variant 2 was the predominant form of CXCR4; however, the other variants may also contribute to chemokine-induced cellular responses. Variants 1 and 5 are likely to produce the same protein, which was the second most abundant but less active variant after variant 2. The long N-terminal region of variant 3 led to inefficient expression and membrane localization, but its activity as a receptor was comparable to variant 2. Based on these results, most CXCL12-stimulated responses may be ascribed to variants 2 and 3. Furthermore, cellular responses to CXCL12 may be influenced by crosstalk of multimerized CXCR4 variants expressed in the cells. Altogether, our results suggest that additional studies of CXCR4 splicing variants and their interactions are warranted and may reveal novel mechanisms underlying elaborate regulation of chemokine response.

## Materials and methods

### Materials

CXCL12 was purchased from Peprotech (Rocky Hill, NJ, USA). NanoBiT® PPI starter system and Nano-Glo® live cell assay system; pBiT3.1 plasmid and Nano-Glo® HiBiT extracellular detection system; and pGL4.33 [luc2P SRE Hygro] and ONE-Glo luciferase assay system were purchased from Promega (Madison, WI, USA). Restriction enzymes were obtained from New England Bio Labs (Ipswich, MA, USA). Anti-CXCR4 antibody (Cat. No. ab124824) was purchased from Abcam (Toronto, ON, Canada). Anti-ERK antibody (Cat. No. 4695) and anti-pERK antibody (Thr202/Tyr204) (Cat. No. 4370) were obtained from Cell Signaling Technology (Beverly, MA, USA). Anti-HA antibody (Cat. No. H3663) and anti-FLAG M2 affinity gel (Cat. No. A2220) for co-IP were purchased from Sigma-Aldrich (St. Louis, MO, USA). Anti-Green fluorescence protein (GFP) antibody (Cat. No. sc-8334), anti-β-actin antibody (Cat. No. sc-9996), and all secondary antibodies were purchased from Santa Cruz Biotechnology (Santa Cruz, CA, USA). All chemicals were obtained from Sigma-Aldrich unless otherwise stated.

### Cell culture

HEK293, HEK293T, HeLa, THP-1, U937, SKOV3, A673, A549, and NCI-H460 cells were obtained from the American Type Culture Collection (ATCC, Manassas, VA, USA). HEK293, HEK293T, and HeLa cells were cultured in Dulbecco’s modified Eagle’s medium (DMEM) supplemented with 10% fetal bovine serum (FBS), 100 U/ml penicillin G, and 100 μg/ml streptomycin (Invitrogen, Carlsbad, CA, USA) at 37°C in a humidified incubator with 5% CO_2_. The other cells were maintained with RPMI1640 media containing all components as described above.

### RT-PCR

Total RNA was isolated from cell lines with TRIzol Reagent (Invitrogen, Carlsbad, CA, USA), according to the manufacturer’s instructions. RNA was denatured by heating samples at 70°C for 5 min, then Moloney murine leukemia virus reverse transcriptase from Promega and random hexamer primers were used to reverse transcribe all messenger RNA species to complementary DNA (cDNA) at 37°C for 60 min in a thermocycler. RT-generated cDNA encoding CXCR4 splice variants was amplified by 35 cycles of 95°C/30 sec, 58°C/30 sec, and 72°C/30 sec with a final extension at 72°C for 10 min. The primers used for PCR were as follows: CXCR4 V1, F- CGTCTCAGTGCCCTTTTGTTC, R- GGTAACCCATGACCAGGATGACC; CXCR4 V2, F- GCCTGAGTGCTCCAGTAGCCAC, R- AGGGAAGCGTGATGACAAAGAG; CXCR4 V3 and V4 F- AGTGATAAACACGAGGATGGCAA, R- AGGGAAGCGTGATGACAAAGAG; and CXCR4 V5, F- CATGTGTCTCCCCCTTGAGTC, R- ATCTCCTCCTGGGAACTCAGC. Ten microliters of all PCR products were then separated by electrophoresis on 1.5% agarose gels.

### Establishment of CXCR4 knock-out cells by CRISPR-Cas9 and reconstitution of CXCR4 variants

To establish cells lacking CXCR4, four potential target sequences were selected using a guide design program from the Zhang Lab (https://zlab.bio/guide-design-resources). A set of two single-stranded oligonucleotides was inserted into the pRG2 vector, which served as a guide vector, and oligonucleotides with 49 nucleotides containing the target sequence were inserted into the pMRS surrogate vector. These two vectors along with the p3SCas9 plasmid were introduced into HEK293 cells, and guide efficiency was examined by genomic DNA PCR and T7E1 treatment. The most efficient guide vector (CXCR4: TACACCGAGGAAATGGGCTCAGG and GAAGCATGACGGACAAGTACAGG), surrogate vector, and p3S-Cas9 were transfected into HEK293cells and HeLa cells. KO cells were isolated by MACSelect Kk MicroBeads (Miltenyi Biotec, Bergisch Gladbach, Germany) and cultured in 96-well microplates at a density of 0.5 cells/well. Gene KO was confirmed by genomic DNA PCR and T7E1 analysis. To characterize each CXCR4 variant, CXCR4 KO HeLa cells were infected with FG12 vector-based lentivirus harboring each CXCR4 variant. Viral supernatants from HEK293T cells were introduced into HeLa cells with 5μg/ml polybrene to enhance infection efficiency.

### Construction of expression plasmids

The CMV promoter sequence in the pcDNA3.1 vector was replaced with the promoter sequence of either elongation factor-1 alpha (EF1α) or ubiquitin C (UbiC) to develop a regulated expression system. Each CXCR4 variant was inserted into these vectors. All genes for the NanoBiT assay, including fragments of the Nano-Luciferase gene in the NanoBiT vectors, were constructed as N-terminal or C-terminal tagged forms in the vectors containing CMV or UbiC promoters.

### Western blotting and co-immunoprecipitation

HEK293 or HeLa CXCR4 KO cells expressing with each variant were incubated with serum-free medium overnight and treated with 100 ng/ml CXCL12 for 5 min for endogenous ERK phosphorylation analysis. After washing with PBS, cells were lysed with lysis buffer (150 mM NaCl, 50 mM Tris-HCl pH7.5, 10 mM KCl, 1% Triton X-100, 10 mM NaF, 5 mM Na_3_VO_4_) and protease inhibitor cocktail (Roche, Indianapolis, IN, USA). Protein quantification was performed with a Bradford protein assay kit (Bio-Rad, Hercules, CA, USA). Samples were denatured with SDS sample buffer, boiled at 100°C for 5 min, and separated by 10% sodium dodecyl sulfate polyacrylamide gel electrophoresis (SDS-PAGE). After proteins were transferred to a nitrocellulose membrane, the membrane was blocked in 5% non-fat dried milk in Tris-buffered saline with Tween 20 (TBS-T) for 30 min and then incubated with primary anti-ERK or anti-pERK antibody overnight at 4°C. The membrane was washed three times in TBS-T for 10 min each and incubated with horseradish peroxidase-conjugated secondary antibody for 1 hr at room temperature. After washing with TBS-T, protein bands were visualized using an enhanced chemiluminescence (ECL) reagent (Thermo Scientific, Rockford, IL, USA). For receptor protein expression analysis, HEK293 cells were transfected with plasmids containing each CXCR4 variant and lysed with lysis buffer containing 6 M urea. Anti-CXCR4 antibody was used to detect each variant. For co-immunoprecipitation, HEK293 cells were transfected with FLAG-tagged CXCR4 variant 2 and HA-tagged CXCR4 variants and incubated for 36 hr. The lysates were incubated with anti-FLAG M2 affinity gel and washed several times with lysis buffer followed by western blotting with anti-HA antibody. Samples for CXCR4 detection were not boiled.

### HiBiT assay

Expression of receptors on the cell membrane was detected using the Nano-Glo® HiBiT extracellular detection system. Briefly, HEK293 cells were seeded in a 96-well microplate at a density of 3 × 10_4_ cells per well. The next day, cells were transfected with each SmBiT-CXCR4 variant plasmid. After 24 hr, the medium was replaced with 100 μl of Nano-Glo HiBiT extracellular reagent (1 μl of LgBiT protein + 2 μl substrate + 97μl of Nano-Glo HiBiT buffer), and cells were equilibrated to room temperature for 4 min. Finally, luciferase activity was measured using a SpectraMax L plate reader (Molecular Devices, San Jose, CA, USA).

### NanoLuc Binary Technology (NanoBiT) assay

HEK293 cells were seeded in a 96-well microplate at a density of 3 × 10_4_ cells per well. The next day, cells were transfected with 50 ng of receptor plasmid and 50 ng of another plasmid encoding the appropriate protein for each experiment with lipofectamine 2000 (Invitrogen, Carlsbad, CA, USA), following the manufacturer’s instruction. These plasmids carry LgBiT or SmBiT in the N-terminal or C-terminal of the protein. For the three-gene combination, 30 ng each of three plasmids was used for transfection. For calcium assays, cells were transfected with 30 ng of receptor plasmid, 30ng of calmodulin tagged with SmBiT plasmid, and 30 ng of plasmid carrying LgBiT-MYLK2S (calmodulin target sequence in MYLK2: LLKKYLMKRRWKKNFIAVSAANRFKK). After 24 hr of transfection, cells were stabilized in 100μl Opti-MEM at room temperature for 10 min. After 25 μl Nano-Glo Live Cell Reagent (furimazine) was added to each well, baseline luminescence was measured for 10 min using a SpectraMax L plate reader (Molecular Devices, San Jose, CA, USA). Stimulated luminescence was then measured continuously for 1 hr after cells were stimulated with 100 ng/ml CXCL12.

### Cellular imaging

HEK293 cells were cultured on poly-L-lysine-coated cover glass, transfected with plasmids containing EGFP-tagged CXCR4 variants, and incubated for 24 hr. After stimulation with 100 ng/ml CXCL12, cells were fixed with 4% paraformaldehyde solution for 10 min and then stained with DAPI. DAPI and EGFP signals were observed under confocal LSM800 microscope (Carl Zeiss Microimaging Inc., Zena, Germany).

### Reporter gene assay

HEK293 cells stably expressing SRE-luc2P were seeded in 96-well plates at a density of 3 × 10^4^ cells per well and transfected with 50 ng of Gα_qi_ plasmid and 50 ng of each variant plasmid. After 24 hr, cells were treated with 100 ng/ml CXCL12 and incubated at 37°C for 6 hr. Then, 100 μl of ONE-Glo reagent was added, and cells were incubated in the dark at room temperature for 4 min. Luciferase activity of cell extracts was measured using a SpectraMax L plate reader (Molecular Devices, San Jose, CA, USA).

### CCK-8 assay

Cell growth was measured with the Cell Counting Kit-8 (CCK-8) obtained from Dojindo Molecular Technologies, Inc. (Rockville, MD, USA), following the manufacturer’s instructions. HeLa CXCR4 KO cells expressing each CXCR4 variant were seeded in four different 96-well microplates at a density of 4 × 10³ cells per well. Every 24 hr, cells in one plate were treated with CCK-8 solution and incubated for 2 hr at 37°C. Then, the absorbance of each well was measured at 450 nm wavelength using a microplate reader (Molecular Devices, San Jose, CA, USA). Cells in the remaining plates were incubated with DMEM containing only 1% FBS with or without 100 ng/ml CXCL12 for the next 24 hr.

### Wound healing assay

HeLa CXCR4 KO cells expressing each of the CXCR4 variants were seeded into 6-well plates at a density of 8 × 10^5^ cells per well and allowed to reach confluence. The cell monolayers were scratched with a 200 μl pipette tip to form wound gaps, washed thrice with PBS, and incubated with DMEM containing 10% FBS and 100 ng/ml CXCL12. Images of the scratches were recorded at ×40 magnification on an inverted optical microscope (Olympus CKX53, Warsaw, Poland) at 0, 12, 24, and 36 hr. The areas between leading edges were measured with Image J software, and then the rates of wound healing were calculated.

### Statistical analysis

Statistical analysis was performed with unpaired Student’s *t*-tests or ANOVA with PRISM5 software (GraphPad; La Jolla, CA, USA). Group means were further analyzed with Bonferroni’s multiple comparison tests. Data were presented as means ± SD, and all experiments were performed at least three times independently unless otherwise indicated.

## Supporting information

S1 Dataset (PDF)

S1 Raw images (PDF)

S1 Fig CXCL12-stimulated interaction of CXCR4 variant 2 and mini G proteins. HEK293 cells expressing C-terminal SmBiT (A) or LgBiT (B) form of CXCR4 variant2 with each of mini G protein constructs tagged with LgBiT or SmBiT were treated with 100 ng. ml CXCL12. The luminescence changes were measured with luminometer.
(TIF)

S2 Fig CXCL12 has no effect on proliferation of HeLa cells in normal culture conditions. Different types of HeLa cells, such as parental cells, CXCR4 KO cells, and cells reconstituted with each splicing variant of CXCR4, were seeded in 96-well plates at a density of 4 × 10³ cells and cultured in complete media containing 10% FBS with or without 100 ng/ml CXCL12. Every 24 h, cells were subjected to CCK-8 assay.
(TIF)

S3 Fig The effect of each CXCR4 variant on HeLa cell motility. Confluent monolayers of parental HeLa cells and genetically modified HeLa cells were scratched with pipette tips. After washing with PBS to remove floating cells, media containing 10% FBS with 100 ng/ml CXCL12 was added to cell monolayers. The images were captured at 0 h and 36 h.
(TIF)
