# Supplementary material for: The N-terminus of CXCR4 splice variants determines expression and functional properties
Source: PLoS One. 2023 May 4;18(5):e0283015. doi: 10.1371/journal.pone.0283015 (PMC10159351; doi:10.1371/journal.pone.0283015)
Supplement: S1 Dataset — (PDF) [file pone.0283015.s001.pdf]

Fig 2A. CMV/CXCR4V1(ACC)					
(n=3)	Mean		S.D.		Statistical method used
Time(min)	SDF1α	Veh.	SDF1α	Veh.	unpaired Student's t-test
0	1.036029	1.170168	0.168083	0.097914	
0.75	1.034556	1.042695	0.093843	0.046967	
1.5	0.992521	1.055009	0.041972	0.095754	
2.25	1.053107	0.956639	0.043607	0.046446	
3	1.070631	1.066963	0.078693	0.074123	
3.75	1.117195	1.051473	0.089727	0.079768	
4.5	1.092684	1.06024	0.071852	0.033422	
5.25	1.074718	1.04762	0.005633	0.082173	
6	1.047693	1.038734	0.115101	0.163118	
6.75	1.063544	1.048305	0.033943	0.098497	
7.5	0.993721	0.995117	0.032607	0.084804	
8.25	0.992554	0.9575	0.098624	0.106647	
9	1.083469	0.933935	0.086562	0.088489	
9.75	1	1	0	0	
11.25	2.937351	0.984687	0.593591	0.11657	
12	4.073821	0.970025	0.507147	0.106926	
12.75	4.183363	0.854346	0.679948	0.120663	
13.5	3.439199	0.874943	0.469046	0.105583	
14.25	2.874662	1.022133	0.510182	0.155892	
15	2.498979	0.970492	0.024911	0.086812	
15.75	2.100332	0.927612	0.03777	0.115095	
16.5	2.034862	0.937582	0.089627	0.073363	
17.25	1.845048	0.936385	0.171904	0.053469	
18	1.714741	0.841388	0.231132	0.120652	
18.75	1.540492	0.81516	0.185281	0.0913	
19.5	1.468072	0.993454	0.181747	0.167304	
20.25	1.499882	0.870855	0.354954	0.084067	
21	1.355195	0.968899	0.230449	0.144891	
21.75	1.067305	1.0665	0.286578	0.028933	
22.5	1.164147	0.950584	0.238317	0.124638	
23.25	1.109243	0.942588	0.180266	0.107285	
24	1.202405	1.034347	0.21252	0.132931	
24.75	1.062615	0.926534	0.304069	0.050323	
25.5	1.098649	0.907502	0.232984	0.096452	
26.25	1.075494	0.83796	0.099086	0.147899	
27	1.083211	0.885511	0.108042	0.088374	
27.75	1.004172	0.949238	0.080638	0.11699	
28.5	1.232664	0.931288	0.258794	0.209904	
29.25	1.223637	0.97775	0.064641	0.125625	
30	0.975336	0.911199	0.164124	0.118257	
30.75	1.108199	1.007386	0.121071	0.180285	
31.5	1.039731	1.068534	0.111941	0.215426	
32.25	1.099389	0.971002	0.08724	0.084304	
33	1.016579	0.958156	0.134322	0.116543	
33.75	1.088709	0.884803	0.102769	0.193985	
34.5	1.080186	0.969651	0.152068	0.099258	
35.25	1.093474	0.968351	0.279238	0.080653	
36	1.10277	0.977819	0.170382	0.144901	
36.75	1.085745	0.906812	0.198473	0.028715	
37.5	1.069207	0.904412	0.09685	0.107209	
38.25	1.027881	0.943366	0.171432	0.075993	
39	0.970475	0.95679	0.314292	0.131627	
39.75	0.962062	0.966831	0.110849	0.068969	
40.5	1.104283	1.00865	0.063667	0.049256	
41.25	1.054073	0.920435	0.148645	0.034292	

Fig 2A. CMV/CXCR4V2(ACC)					
(n=3)	Mean		S.D.		Statistical method used
Time(min)	SDF1α	Veh.	SDF1α	Veh.	unpaired Student's t-test
0	1.033169	1.000308	0.081493	0.064405	
0.75	1.074311	0.942043	0.040134	0.117298	
1.5	1.11924	1.002852	0.066488	0.046284	
2.25	1.157631	1.021061	0.09369	0.079485	

3	1.108726	1.069567	0.091222	0.074123	
3.75	1.134252	0.977943	0.034394	0.086376	
4.5	1.231672	1.051318	0.164287	0.037432	
5.25	1.102205	0.994986	0.160748	0.073386	
6	1.045496	1.066121	0.119148	0.028811	
6.75	1.122846	1.073008	0.084822	0.132685	
7.5	1.071806	1.018979	0.126328	0.062399	
8.25	1.09591	1.086105	0.081949	0.153152	
9	1.070411	1.069533	0.042228	0.082676	
9.75	1	1	0	0	
11.25	3.686632	0.911651	0.096891	0.117367	
12	4.350047	0.889775	0.56464	0.215403	
12.75	4.104952	0.848606	0.057837	0.064417	
13.5	3.593468	0.958887	0.253325	0.221731	
14.25	3.093414	0.933913	0.145807	0.193642	
15	2.878485	1.009811	0.384348	0.086751	
15.75	2.50502	0.937825	0.349206	0.226055	
16.5	2.334973	0.791596	0.189225	0.133157	
17.25	1.9963	0.949497	0.265567	0.046412	
18	2.020395	0.812194	0.123838	0.197137	
18.75	1.873977	0.824841	0.030914	0.139986	
19.5	1.666138	0.948574	0.271043	0.222915	
20.25	1.654609	0.878702	0.194956	0.139497	
21	1.478948	0.913016	0.18013	0.118674	
21.75	1.345171	1.047285	0.324593	0.111905	
22.5	1.351254	0.987526	0.243899	0.024676	
23.25	1.298483	0.932878	0.243315	0.109666	
24	1.301072	0.877373	0.296246	0.05277	
24.75	1.175884	0.887852	0.386469	0.037211	
25.5	1.177129	0.842409	0.0981	0.13295	
26.25	1.142931	0.856807	0.19008	0.214576	
27	1.233491	0.951985	0.250526	0.149104	
27.75	1.117191	0.88777	0.16693	0.083608	
28.5	1.083239	0.947313	0.103515	0.062718	
29.25	1.173115	0.919411	0.045009	0.07168	
30	1.00689	0.808042	0.236235	0.051054	
30.75	1.118132	0.92237	0.087182	0.128734	
31.5	1.199152	0.927881	0.069937	0.024302	
32.25	1.210459	0.899608	0.143792	0.023437	
33	1.04757	0.916525	0.256914	0.235061	
33.75	1.113713	0.800867	0.119108	0.406441	
34.5	1.106413	0.935689	0.258099	0.220534	
35.25	1.100966	0.943625	0.311073	0.234287	
36	1.096244	0.991045	0.20652	0.061626	
36.75	1.16374	0.852549	0.202213	0.136932	
37.5	1.078162	0.842226	0.153402	0.116971	
38.25	1.053503	0.884738	0.178598	0.127282	
39	1.102197	0.905269	0.265528	0.145081	
39.75	1.097487	0.887758	0.13808	0.144674	
40.5	1.050922	0.998858	0.016156	0.050953	
41.25	1.004861	0.903057	0.1763	0.141626	

**Fig 2B. UbiC/CXCR4V1(ACC)**					
(n=3)	Mean		S.D.		Statistical method used
Time(min)	SDF1α	Veh.	SDF1α	Veh.	unpaired Student's t-test
0	1.167568	1.112247	0.206492	0.079879	
0.62	1.104289	1.073322	0.111751	0.064146	
1.24	1.093	1.085945	0.069058	0.038193	
1.86	1.080716	1.07862	0.055955	0.044027	
2.48	1.088583	1.07964	0.036324	0.049386	
3.1	1.08426	1.072594	0.040891	0.046431	
3.72	1.088954	1.084613	0.021471	0.031977	
4.34	1.088659	1.088545	0.020517	0.030669	
4.96	1.084712	1.07695	0.026523	0.038198	
5.58	1.08343	1.075342	0.011929	0.028491	

6.2	1.080763	1.070038	0.004233	0.025	
6.82	1.064074	1.055549	0.006726	0.015066	
7.44	1.057114	1.053467	0.007265	0.023354	
8.06	1.042798	1.035504	0.002515	0.018229	
8.68	1.032748	1.035825	0.017643	0.007227	
9.3	1.025732	1.01791	0.008665	0.003061	
9.92	1.006966	1.015037	0.011337	0.013284	
10.54	1	1	0	0	
11.75	1.68225	0.912252	0.099133	0.00355	
12.37	1.45254	0.877491	0.138296	0.019739	
12.99	1.228909	0.857991	0.119849	0.0107	
13.61	1.05373	0.841085	0.070178	0.008612	
14.23	0.963449	0.830243	0.046939	0.020319	
14.85	0.909406	0.819426	0.036031	0.00553	
15.47	0.879976	0.799447	0.034017	0.00781	
16.09	0.847963	0.801154	0.018322	0.0072	
16.71	0.830684	0.79164	0.015859	0.011982	
17.33	0.826022	0.796694	0.017569	0.024273	
17.95	0.819589	0.792971	0.014967	0.01016	
18.57	0.820602	0.7777	0.006399	0.01845	
19.19	0.808746	0.793987	0.016423	0.02167	
19.81	0.812705	0.783826	0.016007	0.024231	
20.43	0.811048	0.784281	0.018518	0.017254	
21.05	0.804705	0.796321	0.016615	0.039423	
21.67	0.807018	0.788695	0.019338	0.02897	
22.29	0.796878	0.777636	0.018176	0.025037	
22.91	0.81381	0.776454	0.01642	0.021756	
23.53	0.804695	0.780561	0.00574	0.022774	
24.15	0.791006	0.775897	0.011051	0.018846	
24.77	0.788592	0.771874	0.00859	0.016935	
25.39	0.779857	0.763677	0.008328	0.017691	
26.01	0.774291	0.759655	0.019996	0.021633	
26.63	0.773719	0.763843	0.017744	0.012483	
27.25	0.759861	0.752511	0.012495	0.02629	
27.87	0.761832	0.746199	0.010395	0.013024	
28.49	0.75084	0.738759	0.021696	0.014518	
29.11	0.759639	0.747089	0.020042	0.018074	
29.73	0.756671	0.736306	0.029784	0.022922	
30.35	0.738975	0.733402	0.021763	0.027169	
30.97	0.75518	0.715709	0.022779	0.019262	
31.59	0.746244	0.710844	0.010835	0.027423	
32.21	0.743684	0.71966	0.016446	0.028722	
32.83	0.729696	0.703834	0.013784	0.018436	
33.45	0.726697	0.707249	0.020778	0.017269	
34.07	0.717436	0.687696	0.026914	0.022651	
34.69	0.719205	0.692414	0.020682	0.024652	
35.31	0.720097	0.683378	0.001966	0.022635	
35.93	0.703882	0.678314	0.017037	0.015796	
36.55	0.690883	0.676576	0.01997	0.022103	
37.17	0.685479	0.671486	0.027671	0.027516	
37.79	0.680004	0.668927	0.026524	0.021058	
38.41	0.680265	0.663671	0.018653	0.023593	
39.03	0.665647	0.657503	0.021181	0.014803	
39.65	0.669751	0.658259	0.020744	0.018319	
40.27	0.659316	0.654727	0.021202	0.020738	
40.89	0.662724	0.647343	0.019147	0.020826	
41.51	0.653621	0.639926	0.025423	0.030768	
**Fig 2B. UbiC/CXCR4V2(ACC)**					
(n=3)	Mean		S.D.		Statistical method used
Time(min)	SDF1α	Veh.	SDF1α	Veh.	unpaired Student's t-test
0	1.149918	1.047504	0.067577	0.132646	
0.62	1.123467	1.051356	0.082723	0.083607	
1.24	1.090372	1.080227	0.057724	0.053852	
1.86	1.091183	1.115404	0.055266	0.075895	
2.48	1.100588	1.103539	0.061374	0.058589	

3.1	1.116599	1.096052	0.078576	0.032978	
3.72	1.13507	1.094132	0.035322	0.022901	
4.34	1.130635	1.078376	0.069298	0.020541	
4.96	1.112385	1.08925	0.039572	0.033893	
5.58	1.083292	1.079076	0.02598	0.039223	
6.2	1.076946	1.057219	0.012034	0.034298	
6.82	1.057067	1.070038	0.012273	0.040382	
7.44	1.047249	1.0499	0.016783	0.013977	
8.06	1.032406	1.041965	0.017341	0.045126	
8.68	1.031116	1.029482	0.004524	0.030772	
9.3	1.040445	1.019883	0.01674	0.008943	
9.92	1.018513	1.017879	0.008598	0.008596	
10.54	1	1	0	0	
11.75	3.596209	0.914405	0.159238	0.019823	
12.37	3.438264	0.892414	0.124853	0.022221	
12.99	3.196317	0.872768	0.209312	0.024253	
13.61	2.844989	0.866887	0.09318	0.024242	
14.23	2.496794	0.859056	0.243967	0.023877	
14.85	2.13582	0.857902	0.080097	0.026469	
15.47	2.139116	0.854376	0.33722	0.015798	
16.09	1.839707	0.844606	0.14734	0.013205	
16.71	1.779048	0.841811	0.276996	0.010634	
17.33	1.582802	0.855225	0.156188	0.01176	
17.95	1.435973	0.840815	0.130106	0.014867	
18.57	1.335816	0.84176	0.104413	0.020767	
19.19	1.247268	0.837272	0.04953	0.015169	
19.81	1.213333	0.825792	0.095193	0.012732	
20.43	1.129002	0.825331	0.05672	0.012323	
21.05	1.121338	0.828151	0.055304	0.030861	
21.67	1.072162	0.823169	0.021872	0.016252	
22.29	1.021686	0.80569	0.022867	0.015117	
22.91	1.012792	0.807938	0.032862	0.003638	
23.53	0.983144	0.792964	0.020078	0.020464	
24.15	0.934012	0.783562	0.033069	0.024422	
24.77	0.961952	0.771266	0.037645	0.024531	
25.39	0.934731	0.776356	0.06282	0.032951	
26.01	0.909575	0.772424	0.03659	0.023154	
26.63	0.889302	0.766196	0.058726	0.027996	
27.25	0.867669	0.761746	0.022696	0.01803	
27.87	0.837911	0.77486	0.024383	0.003306	
28.49	0.840088	0.763011	0.032813	0.00818	
29.11	0.841718	0.754893	0.025238	0.012487	
29.73	0.85776	0.759685	0.060871	0.015808	
30.35	0.844415	0.748353	0.061303	0.016064	
30.97	0.839246	0.739752	0.040791	0.013662	
31.59	0.815486	0.740244	0.05849	0.017458	
32.21	0.79096	0.726722	0.05237	0.019034	
32.83	0.788568	0.723853	0.054178	0.016892	
33.45	0.771651	0.720389	0.03554	0.019818	
34.07	0.774344	0.719299	0.036037	0.021546	
34.69	0.797909	0.731972	0.050298	0.027321	
35.31	0.770744	0.72904	0.034453	0.041816	
35.93	0.770268	0.717674	0.077729	0.017548	
36.55	0.751223	0.7074	0.050109	0.022867	
37.17	0.741574	0.700263	0.04378	0.021661	
37.79	0.736657	0.688819	0.049011	0.031198	
38.41	0.737533	0.682102	0.045459	0.025497	
39.03	0.725632	0.68357	0.051626	0.019056	
39.65	0.737078	0.681021	0.049831	0.021782	
40.27	0.705799	0.677764	0.026382	0.018927	
40.89	0.716156	0.670956	0.067499	0.025033	
41.51	0.69876	0.671727	0.055759	0.020674	

(n=3)	Mean			S.D.			Statistical method used
DNA amount	0.5ng	5ng	50ng	0.5ng	5ng	50ng	unpaired Student's t-test

**Fig 2F. HiBiT(CMV)**							
Vector	220.25	508.25	2309.75	101.6116	281.6397	377.2695	
V1	10904	83052.25	742008	2044.333	11847.6	88612.57	
V2	16115.75	157022	972780.3	3603.625	23437.29	84592.68	
V3	307.5	1155	5733.5	123.6136	597.4094	873.5539	
V4	298	2070.5	16243.25	73.95043	1003.808	3109.436	
V5	2623	15662.75	160760.8	125.3236	2610.953	28699.08	
**Fig 2F. HiBiT(UbiC)**							
Vector	773.6667	424.6667	379	130.416	224.3687	116.9658	
V1	2126.667	10791	221185.3	128.1575	2028.225	27292.96	
V2	5856.667	40014.33	763809.7	1028.696	4991.002	79144.91	
V3	664	535.3333	1834	233.4888	60.92892	497.5008	
V4	377.3333	761.3333	8817.667	56.35897	364.7524	3312.268	
V5	1174.667	5973	144391.7	236.5002	1365.23	23074.49	

**Fig 3A. CMV/CXCR4V1(TGA)**					
(n=3)	Mean		S.D.		Statistical method used
Time(min)	SDF1α	Veh.	SDF1α	Veh.	unpaired Student's t-test
0	1.027633	0.966512	0.06596	0.069768	
0.75	1.046746	0.969232	0.163641	0.076974	
1.5	1.044803	1.032938	0.118663	0.057059	
2.25	0.966793	0.980712	0.123729	0.083971	
3	0.958314	1.049925	0.141534	0.184633	
3.75	0.992369	1.052931	0.150661	0.129438	
4.5	1.018964	1.034425	0.109079	0.059664	
5.25	1.050013	1.071639	0.153969	0.135556	
6	1.0931	1.067157	0.225786	0.204713	
6.75	1.025465	1.049911	0.213709	0.164259	
7.5	0.915414	1.024942	0.114133	0.09253	
8.25	0.881791	0.99212	0.084969	0.115045	
9	1.032829	0.981705	0.124577	0.078684	
9.75	1	1	0	0	
11.25	0.936021	0.892712	0.159735	0.095113	
12	1.079575	0.866019	0.269802	0.022191	
12.75	1.145272	0.80084	0.371584	0.049377	
13.5	0.908353	0.859299	0.201547	0.103288	
14.25	0.765236	0.782134	0.104005	0.113669	
15	0.838277	0.758899	0.106144	0.039705	
15.75	0.841306	0.755513	0.078966	0.070105	
16.5	0.857725	0.784846	0.016716	0.068089	
17.25	0.758923	0.771622	0.03234	0.115023	
18	0.744873	0.770353	0.064516	0.114826	
18.75	0.702282	0.824125	0.027784	0.081542	
19.5	0.743362	0.705958	0.096263	0.090224	
20.25	0.758287	0.790727	0.089048	0.071235	
21	0.760852	0.742665	0.093697	0.06525	
21.75	0.741801	0.758417	0.105139	0.074435	
22.5	0.730114	0.877366	0.060751	0.25164	
23.25	0.70027	0.801087	0.005821	0.096576	
24	0.769544	0.848221	0.156519	0.093959	
24.75	0.728447	0.681331	0.114177	0.093116	
25.5	0.729068	0.689404	0.152584	0.095677	
26.25	0.68265	0.724375	0.123764	0.11686	
27	0.700291	0.6944	0.119434	0.15403	
27.75	0.667653	0.718424	0.130252	0.078209	
28.5	0.724742	0.657761	0.135846	0.126486	
29.25	0.670042	0.703687	0.140388	0.061245	
30	0.731056	0.678029	0.139774	0.032162	
30.75	0.681517	0.743621	0.027897	0.10788	
31.5	0.709736	0.765372	0.106986	0.108808	
32.25	0.648855	0.714127	0.045819	0.143155	
33	0.707904	0.677903	0.108151	0.168173	
33.75	0.704303	0.661981	0.205857	0.052205	

34.5	0.679928	0.761902	0.063554	0.065813	
35.25	0.648128	0.642438	0.097012	0.039401	
36	0.645907	0.642469	0.067616	0.095319	
36.75	0.643231	0.692392	0.125988	0.075836	
37.5	0.663706	0.701475	0.145042	0.113903	
38.25	0.63639	0.64697	0.11388	0.125541	
39	0.649703	0.703729	0.101261	0.094157	
39.75	0.684504	0.675308	0.097757	0.124384	
40.5	0.630894	0.69558	0.07063	0.035223	
41.25	0.661913	0.650742	0.168752	0.126384	
**Fig 3A. CMV/CXCR4V2**					
(n=3)	Mean		S.D.		Statistical method used
Time(min)	SDF1α	Veh.	SDF1α	Veh.	unpaired Student's t-test
0	1.301331	1.086009	0.158197	0.055646	
0.75	1.14868	1.080807	0.172585	0.050137	
1.5	1.035866	1.090642	0.117082	0.080115	
2.25	1.02986	1.127498	0.110855	0.029271	
3	1.071725	1.107149	0.025779	0.094264	
3.75	1.02012	1.01679	0.035942	0.075436	
4.5	1.068816	1.095783	0.029728	0.08026	
5.25	1.012536	1.009624	0.037515	0.071084	
6	0.984859	1.066449	0.074218	0.148189	
6.75	0.969301	1.006552	0.047699	0.199801	
7.5	0.95347	0.98749	0.060203	0.124789	
8.25	1.352392	1.105832	0.512762	0.050642	
9	0.989634	1.053528	0.217337	0.081208	
9.75	1	1	0	0	
11.25	3.799225	0.896964	1.15377	0.069061	
12	4.156869	0.835189	0.808235	0.065405	
12.75	3.692917	0.908334	0.61045	0.022316	
13.5	2.952296	0.888262	0.355914	0.03022	
14.25	2.57563	0.852844	0.195668	0.062511	
15	2.349016	0.821753	0.223298	0.056627	
15.75	1.946877	0.867588	0.338022	0.096474	
16.5	1.619061	0.86144	0.065865	0.096428	
17.25	1.641306	0.829356	0.381832	0.090958	
18	1.567613	0.781312	0.297942	0.091309	
18.75	1.336564	0.815408	0.200535	0.099254	
19.5	1.317082	0.840377	0.183017	0.045177	
20.25	1.307208	0.780159	0.340861	0.09574	
21	1.237935	0.789384	0.271557	0.090279	
21.75	1.148663	0.820263	0.204942	0.107417	
22.5	1.049462	0.715923	0.102335	0.033729	
23.25	1.033243	0.741346	0.241459	0.033892	
24	1.09269	0.723589	0.171983	0.092844	
24.75	1.12978	0.764742	0.234517	0.05066	
25.5	0.923069	0.745621	0.05809	0.029157	
26.25	0.916595	0.734212	0.197682	0.040414	
27	0.859268	0.750176	0.075801	0.100331	
27.75	0.867261	0.747146	0.141289	0.028952	
28.5	0.942122	0.702685	0.332454	0.027204	
29.25	0.867512	0.741286	0.04423	0.027514	
30	0.836765	0.714377	0.216762	0.096953	
30.75	0.880658	0.690997	0.131413	0.109465	
31.5	0.723955	0.715454	0.130973	0.089924	
32.25	0.826355	0.689285	0.091531	0.125551	
33	0.908344	0.71663	0.214044	0.128898	
33.75	0.787506	0.692928	0.082824	0.049033	
34.5	0.75502	0.662488	0.062531	0.032579	
35.25	0.700721	0.707152	0.03868	0.095417	
36	0.765839	0.655444	0.223146	0.014729	
36.75	0.68593	0.655684	0.042053	0.024485	
37.5	0.701118	0.681418	0.050511	0.110825	
38.25	0.695543	0.794504	0.06434	0.207968	
39	0.774681	0.710375	0.186083	0.095938	

39.75	0.779899	0.666868	0.071606	0.090313	
40.5	0.861613	0.65348	0.40996	0.131395	
41.25	0.870722	0.596994	0.267366	0.067756	

**Fig 3A. CMV/CXCR4V3**					
(n=3)	Mean		S.D.		Statistical method used
Time(min)	SDF1α	Veh.	SDF1α	Veh.	unpaired Student's t-test
0	1.157876	1.077897	0.241402	0.052026	
0.75	1.137294	1.159836	0.193935	0.071771	
1.5	1.190314	1.126159	0.169739	0.022747	
2.25	1.182348	1.103063	0.137406	0.097686	
3	1.157014	1.164398	0.043593	0.014943	
3.75	1.153228	1.095478	0.131424	0.134767	
4.5	1.118283	1.017714	0.077981	0.196477	
5.25	1.045565	1.140141	0.067994	0.062336	
6	1.020694	1.013788	0.169583	0.094605	
6.75	1.032333	0.993748	0.071586	0.10107	
7.5	1.126893	1.084693	0.16055	0.099067	
8.25	1.021523	1.04283	0.166598	0.11374	
9	1.008238	1.032435	0.097148	0.048566	
9.75	1	1	0	0	
11.25	4.208058	0.979513	1.871917	0.268407	
12	6.045511	0.874633	1.019647	0.146167	
12.75	4.724595	0.839627	0.833538	0.090834	
13.5	3.566547	0.866783	0.807912	0.064708	
14.25	2.838333	0.868415	0.477004	0.030469	
15	1.993095	0.836171	0.184354	0.067586	
15.75	1.702148	0.843705	0.418382	0.064434	
16.5	1.299918	0.829529	0.198627	0.069046	
17.25	1.177143	0.775137	0.068071	0.052349	
18	1.079206	0.733865	0.153904	0.054094	
18.75	0.981744	0.78395	0.138844	0.054248	
19.5	0.970415	0.801541	0.098824	0.07617	
20.25	1.003033	0.783143	0.098091	0.048605	
21	0.85633	0.727068	0.106141	0.059785	
21.75	0.883031	0.752182	0.135087	0.073482	
22.5	0.864185	0.868996	0.188092	0.129169	
23.25	0.820379	0.738712	0.106398	0.036895	
24	0.808233	0.754948	0.05642	0.040183	
24.75	0.830275	0.788194	0.186662	0.022415	
25.5	0.796354	0.673555	0.051114	0.031085	
26.25	0.778874	0.727289	0.099242	0.051761	
27	0.790204	0.695463	0.041014	0.038806	
27.75	0.843087	0.785694	0.044102	0.124205	
28.5	0.823839	0.701317	0.044376	0.048568	
29.25	0.790981	0.689643	0.088683	0.070532	
30	0.806898	0.748264	0.146967	0.082225	
30.75	0.722406	0.708007	0.086716	0.093057	
31.5	0.801129	0.719896	0.126431	0.03314	
32.25	0.737287	0.708841	0.06286	0.058563	
33	0.748663	0.723801	0.115398	0.073365	
33.75	0.742727	0.667324	0.130101	0.065249	
34.5	0.676253	0.660636	0.048397	0.036263	
35.25	0.737018	0.666697	0.0212	0.079891	
36	0.692462	0.654073	0.082129	0.037355	
36.75	0.727293	0.689478	0.097613	0.073824	
37.5	0.723673	0.71616	0.030525	0.028587	
38.25	0.702245	0.723252	0.072787	0.072226	
39	0.616804	0.687165	0.096079	0.051956	
39.75	0.677531	0.779642	0.144361	0.140905	
40.5	0.686996	0.694828	0.099597	0.038651	
41.25	0.629433	0.725396	0.098145	0.123584	

**Fig 3A. CMV/CXCR4V4**					
(n=3)	Mean		S.D.		Statistical method used
Time(min)	SDF1α	Veh.	SDF1α	Veh.	unpaired Student's t-test
0	1.201442	1.059009	0.242043	0.171142	

0.75	1.052751	1.099416	0.06875	0.182527	
1.5	1.095928	1.128764	0.040676	0.131889	
2.25	1.118936	1.091937	0.054686	0.105494	
3	1.163963	1.09408	0.086553	0.051891	
3.75	1.146213	1.077807	0.129611	0.092416	
4.5	1.072496	1.06173	0.036385	0.097647	
5.25	1.115012	1.06091	0.139434	0.072944	
6	1.097885	1.055575	0.116727	0.036083	
6.75	1.087004	1.047214	0.107132	0.074796	
7.5	1.038371	0.985691	0.026096	0.062936	
8.25	1.033765	1.028487	0.065798	0.125241	
9	1.06845	1.001499	0.138272	0.079531	
9.75	1	1	0	0	
11.25	1.754563	0.819819	0.220998	0.087375	
12	1.152875	0.909099	0.19901	0.091986	
12.75	1.176087	0.883889	0.18888	0.081389	
13.5	0.977217	0.875539	0.125887	0.042139	
14.25	0.8741	0.885814	0.065732	0.07817	
15	0.898589	0.918557	0.071781	0.048501	
15.75	0.830525	0.9024	0.085988	0.153599	
16.5	0.901967	0.847921	0.062334	0.056366	
17.25	0.829041	0.762336	0.079886	0.085138	
18	0.792681	0.849276	0.0241	0.123672	
18.75	0.85062	0.825778	0.024114	0.045854	
19.5	0.830287	0.800216	0.109184	0.008589	
20.25	0.811265	0.79826	0.108071	0.085326	
21	0.848412	0.855203	0.039352	0.084255	
21.75	0.763916	0.777559	0.108152	0.097195	
22.5	0.77794	0.784133	0.063944	0.106237	
23.25	0.78555	0.796979	0.067763	0.034881	
24	0.822202	0.838903	0.039568	0.056317	
24.75	0.818519	0.757923	0.053202	0.04929	
25.5	0.808725	0.769738	0.114116	0.036173	
26.25	0.766594	0.787519	0.03839	0.085021	
27	0.821318	0.7813	0.074472	0.087942	
27.75	0.72775	0.768382	0.086507	0.038175	
28.5	0.728723	0.741492	0.008919	0.075957	
29.25	0.75176	0.757168	0.070795	0.066802	
30	0.752567	0.819564	0.083466	0.089589	
30.75	0.749738	0.65777	0.086821	0.091793	
31.5	0.776969	0.742306	0.090575	0.084591	
32.25	0.701164	0.697909	0.059443	0.046923	
33	0.770679	0.758394	0.055531	0.050043	
33.75	0.684428	0.776303	0.108669	0.016988	
34.5	0.668358	0.693157	0.038148	0.025599	
35.25	0.722953	0.67066	0.129462	0.134376	
36	0.671366	0.69986	0.043483	0.010884	
36.75	0.696626	0.745032	0.032574	0.069216	
37.5	0.653129	0.637232	0.089068	0.027496	
38.25	0.744153	0.697436	0.173475	0.017912	
39	0.629706	0.676556	0.068265	0.044635	
39.75	0.662127	0.710194	0.088689	0.060298	
40.5	0.699673	0.640455	0.09621	0.087703	
41.25	0.654206	0.631652	0.080104	0.023019	

Fig 3A. CMV/CXCR4V5

(n=3)	Mean		S.D.		Statistical method used
Time(min)	SDF1α	Veh.	SDF1α	Veh.	unpaired Student's t-test
0	1.147609	1.165792	0.192361	0.040765	
0.75	1.104724	1.194406	0.037941	0.091531	
1.5	1.195309	1.157947	0.136091	0.041575	
2.25	1.242535	1.092747	0.067358	0.091733	
3	1.18545	1.077291	0.091592	0.049679	
3.75	1.146928	1.03788	0.063274	0.128507	
4.5	1.107328	1.081281	0.04207	0.057721	
5.25	1.084997	1.046433	0.050462	0.078563	

6	1.082608	1.045129	0.104938	0.034756	
6.75	1.025824	0.988085	0.125844	0.093147	
7.5	1.014782	1.051641	0.061791	0.081889	
8.25	0.998822	0.943659	0.082139	0.070564	
9	0.997637	0.979748	0.085065	0.035101	
9.75	1	1	0	0	
11.25	1.008746	0.913881	0.017411	0.054458	
12	1.182185	0.90493	0.220433	0.014829	
12.75	1.341245	0.856555	0.218867	0.080749	
13.5	1.118648	0.891632	0.21337	0.040981	
14.25	0.877264	0.864029	0.068638	0.043703	
15	0.830457	0.845767	0.141358	0.032093	
15.75	0.885016	0.901651	0.089034	0.070809	
16.5	0.774161	0.784607	0.081025	0.076794	
17.25	0.820782	0.846663	0.044623	0.016173	
18	0.780106	0.830158	0.064806	0.046898	
18.75	0.859042	0.839982	0.048986	0.085649	
19.5	0.783173	0.856233	0.038814	0.108595	
20.25	0.841271	0.813623	0.045465	0.094351	
21	0.834699	0.793809	0.067326	0.0364	
21.75	0.857715	0.803522	0.094295	0.072224	
22.5	0.842765	0.738921	0.111491	0.079878	
23.25	0.753791	0.766548	0.070704	0.076058	
24	0.793308	0.850252	0.070991	0.083878	
24.75	0.803412	0.784048	0.026267	0.023639	
25.5	0.801289	0.809709	0.013508	0.043345	
26.25	0.812715	0.737496	0.140275	0.043923	
27	0.764651	0.778115	0.113092	0.027139	
27.75	0.756659	0.760165	0.07332	0.065711	
28.5	0.74178	0.789249	0.064638	0.069344	
29.25	0.739877	0.746895	0.124034	0.126132	
30	0.720999	0.683947	0.087478	0.078422	
30.75	0.698232	0.708283	0.090827	0.097365	
31.5	0.722489	0.757004	0.188455	0.077603	
32.25	0.695182	0.682931	0.061838	0.080831	
33	0.707662	0.660501	0.020404	0.11099	
33.75	0.728155	0.700345	0.067114	0.025911	
34.5	0.706132	0.655391	0.021162	0.015985	
35.25	0.70378	0.609511	0.016107	0.026173	
36	0.655201	0.601862	0.017323	0.064103	
36.75	0.704023	0.628912	0.102289	0.098156	
37.5	0.627654	0.614779	0.03028	0.065436	
38.25	0.634874	0.716043	0.032281	0.077809	
39	0.632112	0.65092	0.035235	0.056382	
39.75	0.640733	0.637227	0.051943	0.048931	
40.5	0.688472	0.614187	0.090071	0.091642	
41.25	0.637842	0.661356	0.032783	0.050464	
**Fig 3A. UbiC/CXCR4V1(TGA)**					
(n=3)	Mean		S.D.		Statistical method used
Time(min)	SDF1α	Veh.	SDF1α	Veh.	unpaired Student's t-test
0	1.118954	1.202013	0.203988	0.145008	
0.75	1.115223	1.155036	0.161993	0.164486	
1.5	1.070243	1.273125	0.244748	0.21547	
2.25	1.071775	1.127693	0.250316	0.083201	
3	1.122577	1.180747	0.186391	0.056174	
3.75	0.989513	1.331359	0.20286	0.171952	
4.5	1.040245	1.216877	0.097047	0.124542	
5.25	1.113835	1.198098	0.12023	0.091825	
6	1.049759	1.12461	0.250577	0.201093	
6.75	1.123622	1.166916	0.187388	0.167502	
7.5	1.047453	1.166524	0.090857	0.165523	
8.25	1.144913	1.118531	0.036674	0.055864	
9	1.031528	1.200815	0.086857	0.190084	
9.75	1	1	0	0	
11.25	2.005998	0.983811	0.356655	0.046022	

12	1.602642	1.009883	0.244691	0.183988	
12.75	1.391429	0.988491	0.306738	0.097078	
13.5	1.153394	1.041037	0.1977	0.11683	
14.25	1.083174	1.035697	0.235362	0.197557	
15	0.917108	0.940571	0.188519	0.123531	
15.75	0.880645	0.978514	0.167704	0.060766	
16.5	0.848253	0.990969	0.230798	0.002989	
17.25	0.843884	0.855871	0.078154	0.105229	
18	0.7818	0.951516	0.115834	0.154129	
18.75	0.869224	0.953466	0.144411	0.186976	
19.5	0.905629	0.945801	0.076285	0.143057	
20.25	0.852289	0.909826	0.048481	0.099752	
21	0.790228	0.927449	0.097923	0.034647	
21.75	0.752047	0.848629	0.150922	0.081639	
22.5	0.800363	0.821291	0.073451	0.11603	
23.25	0.78094	0.899571	0.103647	0.092823	
24	0.76013	0.767776	0.110951	0.108209	
24.75	0.850244	0.797489	0.159664	0.099255	
25.5	0.801872	0.748434	0.113237	0.023466	
26.25	0.755235	0.800434	0.060385	0.068033	
27	0.768371	0.924262	0.119422	0.132836	
27.75	0.793828	0.81187	0.146851	0.130487	
28.5	0.695315	0.842838	0.122758	0.089767	
29.25	0.833599	0.816687	0.124246	0.15563	
30	0.735946	0.794915	0.151923	0.158867	
30.75	0.707118	0.878142	0.030994	0.142252	
31.5	0.736043	0.834926	0.119252	0.079514	
32.25	0.720871	0.809972	0.110646	0.145327	
33	0.756839	0.837926	0.125985	0.142948	
33.75	0.788278	0.731782	0.121566	0.067311	
34.5	0.681783	0.738478	0.17268	0.106377	
35.25	0.690522	0.756935	0.126159	0.162446	
36	0.742057	0.790562	0.128573	0.181883	
36.75	0.69739	0.749799	0.110376	0.062887	
37.5	0.682879	0.790007	0.132515	0.025398	
38.25	0.696009	0.702905	0.124763	0.086442	
39	0.607132	0.77901	0.072958	0.114255	
39.75	0.625961	0.755825	0.054893	0.076772	
40.5	0.667293	0.747416	0.104076	0.055995	
41.25	0.62442	0.710263	0.073141	0.064227	
**Fig 3A. UbiC/CXCR4V2**					
(n=3)	Mean		S.D.		Statistical method used
Time(min)	SDF1α	Veh.	SDF1α	Veh.	unpaired Student's t-test
0	1.073463	1.055425	0.072359	0.175443	
0.75	1.156192	1.10545	0.144722	0.29272	
1.5	0.991664	1.174648	0.132358	0.176136	
2.25	1.114613	1.102305	0.061965	0.064722	
3	1.042174	1.151086	0.136395	0.227589	
3.75	1.077734	1.10544	0.09334	0.079556	
4.5	1.049669	1.063087	0.246906	0.066759	
5.25	1.053387	1.172426	0.150247	0.246579	
6	1.050607	0.999822	0.247627	0.130329	
6.75	0.997142	1.146529	0.184224	0.056393	
7.5	0.986411	1.084929	0.178767	0.056378	
8.25	1.02518	1.102906	0.308089	0.144659	
9	1.089923	1.075325	0.290129	0.134416	
9.75	1	1	0	0	
11.25	4.700732	0.983715	0.47908	0.078785	
12	5.465487	0.859631	0.851515	0.117177	
12.75	4.396888	0.871911	0.662757	0.137129	
13.5	3.738914	0.899917	0.515909	0.121687	
14.25	3.230066	0.869479	0.449517	0.104447	
15	2.529471	0.814828	0.50362	0.111072	
15.75	2.315167	0.806274	0.323502	0.075678	
16.5	2.268354	0.813413	0.29315	0.097254	

17.25	2.097796	0.807921	0.753013	0.10753	
18	1.857283	0.768136	0.383991	0.03745	
18.75	1.808432	0.80966	0.416821	0.069953	
19.5	1.642856	0.848271	0.301055	0.163597	
20.25	1.611347	0.820352	0.31377	0.098296	
21	1.529924	0.861156	0.154163	0.050488	
21.75	1.366942	0.849428	0.188854	0.079504	
22.5	1.386815	0.84202	0.18506	0.203576	
23.25	1.307007	0.805287	0.275236	0.022282	
24	1.193548	0.75761	0.267571	0.030169	
24.75	1.215222	0.761124	0.309966	0.081948	
25.5	1.208199	0.730408	0.287374	0.090899	
26.25	1.150953	0.714251	0.230207	0.014799	
27	1.024877	0.719262	0.33558	0.102708	
27.75	1.145446	0.76731	0.115623	0.1509	
28.5	1.083955	0.724081	0.254876	0.072918	
29.25	1.00359	0.78131	0.146758	0.16238	
30	0.946213	0.64321	0.105297	0.085137	
30.75	1.057984	0.657933	0.239135	0.109116	
31.5	0.984632	0.668194	0.321726	0.022482	
32.25	0.997348	0.725747	0.254277	0.061766	
33	0.895367	0.799029	0.136824	0.07814	
33.75	0.996834	0.693515	0.300521	0.110245	
34.5	0.986611	0.678569	0.32737	0.113407	
35.25	0.901933	0.614628	0.126833	0.008764	
36	0.845039	0.741689	0.234023	0.039413	
36.75	0.777087	0.649057	0.101874	0.0532	
37.5	0.751126	0.6824	0.070877	0.131138	
38.25	0.873174	0.686797	0.257118	0.080505	
39	0.851596	0.717749	0.186183	0.075311	
39.75	0.729788	0.624657	0.058853	0.046545	
40.5	0.77937	0.707514	0.13991	0.091887	
41.25	0.70859	0.544724	0.126382	0.060117	
**Fig 3A. UbiC/CXCR4V3**					
(n=3)	Mean		S.D.		Statistical method used
Time(min)	SDF1α	Veh.	SDF1α	Veh.	unpaired Student's t-test
0	1.166634	1.16027	0.245042	0.222657	
0.75	1.18854	1.225352	0.225535	0.301649	
1.5	1.312279	1.072369	0.240091	0.241277	
2.25	1.202804	1.243808	0.222867	0.335629	
3	1.169105	1.265164	0.080159	0.161404	
3.75	1.158796	1.186429	0.198927	0.035713	
4.5	1.123256	1.185912	0.106907	0.176581	
5.25	1.185788	1.363364	0.244389	0.112456	
6	1.183112	1.227374	0.111569	0.174244	
6.75	1.049441	1.139427	0.131698	0.215353	
7.5	1.104737	1.21169	0.236218	0.198451	
8.25	1.043857	1.171111	0.161046	0.080297	
9	1.029487	1.127932	0.247425	0.288	
9.75	1	1	0	0	
11.25	1.674984	0.98103	0.330932	0.046777	
12	1.295555	1.012464	0.257673	0.156637	
12.75	1.295108	0.984704	0.118626	0.014895	
13.5	0.949507	0.937753	0.178066	0.118855	
14.25	0.914401	0.964334	0.156364	0.097919	
15	0.898246	0.870357	0.080214	0.048011	
15.75	0.84519	0.951367	0.028252	0.116535	
16.5	0.758007	0.837724	0.180989	0.100175	
17.25	0.927655	0.891545	0.199279	0.191649	
18	0.878765	0.834009	0.058683	0.072035	
18.75	0.92302	0.851343	0.158193	0.117709	
19.5	0.844431	0.904919	0.212119	0.134767	
20.25	0.851865	0.820093	0.092053	0.098947	
21	0.819178	0.827992	0.087025	0.189785	
21.75	0.8619	0.749899	0.171473	0.085792	

22.5	0.77487	0.80316	0.155664	0.152593	
23.25	0.873735	0.862666	0.136758	0.121986	
24	0.772034	0.860347	0.125585	0.285734	
24.75	0.822649	0.819498	0.096432	0.11523	
25.5	0.83554	0.80465	0.128091	0.176707	
26.25	0.822912	0.674841	0.119135	0.181709	
27	0.793852	0.73535	0.128149	0.080601	
27.75	0.698449	0.819559	0.029709	0.191364	
28.5	0.702902	0.868226	0.100643	0.227903	
29.25	0.77169	0.729601	0.132476	0.029226	
30	0.750186	0.788857	0.061755	0.140396	
30.75	0.765297	0.735391	0.22803	0.189741	
31.5	0.725495	0.658076	0.089261	0.08477	
32.25	0.69397	0.807882	0.11084	0.153089	
33	0.703468	0.775636	0.088182	0.160925	
33.75	0.771786	0.682381	0.124015	0.06951	
34.5	0.73717	0.682369	0.212088	0.145506	
35.25	0.739393	0.66269	0.180635	0.046066	
36	0.726181	0.676192	0.104026	0.084416	
36.75	0.704649	0.705102	0.134092	0.164015	
37.5	0.660201	0.682101	0.165625	0.091795	
38.25	0.637295	0.664481	0.071703	0.123472	
39	0.63526	0.771552	0.117654	0.187507	
39.75	0.633632	0.616468	0.099977	0.139445	
40.5	0.721506	0.632084	0.119068	0.188683	
41.25	0.681579	0.666776	0.109933	0.123911	
**Fig 3A. UbiC/CXCR4V4**					
(n=3)	Mean		S.D.		Statistical method used
Time(min)	SDF1α	Veh.	SDF1α	Veh.	unpaired Student's t-test
0	1.021749	1.070881	0.191813	0.090616	
0.75	1.067077	1.102975	0.103916	0.201756	
1.5	1.054243	1.000247	0.12916	0.152409	
2.25	1.072148	0.984922	0.05715	0.073269	
3	1.089253	1.086552	0.055416	0.125853	
3.75	1.059031	0.956137	0.09584	0.01976	
4.5	1.046748	0.929318	0.068483	0.180102	
5.25	1.012209	0.951176	0.046639	0.187108	
6	1.118383	1.021437	0.083317	0.060063	
6.75	1.05354	0.970341	0.107393	0.14506	
7.5	0.943043	0.907583	0.168626	0.067932	
8.25	0.987437	0.950738	0.135756	0.18775	
9	1.035191	0.881036	0.082897	0.124586	
9.75	1	1	0	0	
11.25	1.313374	0.71683	0.16513	0.050222	
12	0.94007	0.799563	0.024192	0.017986	
12.75	0.892345	0.846082	0.070925	0.090167	
13.5	0.785477	0.757491	0.062962	0.082209	
14.25	0.811226	0.781696	0.093	0.079917	
15	0.892832	0.747469	0.092633	0.082725	
15.75	0.739177	0.827291	0.087966	0.082914	
16.5	0.79268	0.809124	0.025776	0.155132	
17.25	0.853693	0.759455	0.176412	0.011015	
18	0.792381	0.694047	0.059927	0.125045	
18.75	0.809912	0.689869	0.130022	0.098096	
19.5	0.779248	0.807917	0.066195	0.07222	
20.25	0.770847	0.752233	0.053443	0.061137	
21	0.772637	0.713675	0.111466	0.167856	
21.75	0.692755	0.755324	0.064215	0.100541	
22.5	0.736897	0.711256	0.041588	0.044725	
23.25	0.722531	0.732268	0.0697	0.116414	
24	0.78005	0.702889	0.058031	0.034119	
24.75	0.698998	0.710697	0.098319	0.02658	
25.5	0.708201	0.683737	0.039585	0.085066	
26.25	0.75896	0.697717	0.028512	0.090978	
27	0.680689	0.613643	0.039247	0.126897	

27.75	0.688137	0.654713	0.106716	0.113801	
28.5	0.690653	0.726721	0.097611	0.188781	
29.25	0.746241	0.651673	0.095625	0.040792	
30	0.723744	0.670604	0.118843	0.057221	
30.75	0.675177	0.652968	0.075176	0.072035	
31.5	0.68655	0.642672	0.062979	0.114732	
32.25	0.668651	0.660108	0.030586	0.079542	
33	0.651351	0.667455	0.067349	0.065954	
33.75	0.652924	0.610933	0.078206	0.130492	
34.5	0.577589	0.66157	0.00762	0.010392	
35.25	0.587533	0.544396	0.11442	0.107384	
36	0.63921	0.615875	0.028647	0.139416	
36.75	0.622526	0.6592	0.078027	0.086999	
37.5	0.677232	0.595684	0.079238	0.071657	
38.25	0.602957	0.584538	0.017014	0.063728	
39	0.599328	0.633901	0.088978	0.096173	
39.75	0.607795	0.589924	0.018561	0.043771	
40.5	0.545387	0.545818	0.04398	0.130618	
41.25	0.651268	0.554784	0.123529	0.028451	
**Fig 3A. UbiC/CXCR4V5**					
(n=3)	Mean		S.D.		Statistical method used
Time(min)	SDF1α	Veh.	SDF1α	Veh.	unpaired Student's t-test
0	1.089944	0.985209	0.121533	0.127737	
0.75	1.140109	0.980241	0.13124	0.047858	
1.5	1.098519	1.151145	0.148881	0.149006	
2.25	1.125645	0.975646	0.035235	0.095654	
3	1.218457	0.99262	0.144383	0.029099	
3.75	1.160246	1.034725	0.215569	0.079675	
4.5	1.154165	1.02978	0.100518	0.217236	
5.25	1.184728	1.110693	0.127809	0.222421	
6	1.134675	1.007077	0.153632	0.048144	
6.75	1.114908	1.011671	0.127989	0.163829	
7.5	1.129269	0.9708	0.212929	0.088336	
8.25	1.140096	0.954534	0.10228	0.125698	
9	1.165457	0.962625	0.029215	0.168662	
9.75	1	1	0	0	
11.25	2.787428	0.999669	0.229604	0.100687	
12	2.162872	0.97685	0.261653	0.03151	
12.75	1.708529	0.836971	0.427662	0.130525	
13.5	1.512399	0.840289	0.202469	0.1785	
14.25	1.190164	0.805588	0.19775	0.015274	
15	1.141761	0.885294	0.0826	0.016678	
15.75	1.015418	0.84706	0.091681	0.153722	
16.5	1.013758	0.864	0.135439	0.028942	
17.25	1.015568	0.828838	0.110842	0.091865	
18	0.897967	0.798964	0.137261	0.064446	
18.75	0.851905	0.799435	0.125618	0.094745	
19.5	0.857994	0.819113	0.124051	0.076569	
20.25	0.837239	0.768443	0.119586	0.15698	
21	0.860002	0.80012	0.042326	0.081116	
21.75	0.820767	0.798184	0.158091	0.135852	
22.5	0.87923	0.823247	0.11166	0.102116	
23.25	0.848239	0.754362	0.123171	0.125424	
24	0.823209	0.796694	0.027368	0.081832	
24.75	0.876461	0.826478	0.032631	0.038484	
25.5	0.85703	0.835068	0.082625	0.148475	
26.25	0.781111	0.741487	0.129511	0.085166	
27	0.753616	0.78383	0.11067	0.019035	
27.75	0.790616	0.741104	0.122525	0.022997	
28.5	0.754938	0.849072	0.029285	0.206158	
29.25	0.901197	0.729567	0.289385	0.130697	
30	0.964965	0.809688	0.269519	0.097185	
30.75	0.833431	0.7151	0.247954	0.07908	
31.5	0.861314	0.697441	0.284847	0.136995	
32.25	0.903416	0.66765	0.244193	0.023876	

33	0.86532	0.735908	0.355057	0.104797	
33.75	0.781635	0.714371	0.204687	0.071417	
34.5	0.80001	0.717439	0.135199	0.03233	
35.25	0.862583	0.690397	0.254573	0.088834	
36	0.738439	0.656213	0.218571	0.080409	
36.75	0.724119	0.63947	0.041082	0.045968	
37.5	0.748573	0.726275	0.079015	0.218254	
38.25	0.819501	0.686918	0.135065	0.196202	
39	0.631339	0.693657	0.101984	0.119848	
39.75	0.729317	0.683814	0.15953	0.047641	
40.5	0.714684	0.659577	0.184153	0.088125	
41.25	0.69114	0.645093	0.171936	0.085615	

(n=3)	Mean	S.D.	Statistical method used	P value
**Fig 3B. CMV promoter**			unpaired Student's t-test	**p<0.01 *p<0.05
NT	1.032829	0.124577		
V1	1.079575	0.269802		
V2	4.156869	0.808235		
V3	6.045511	0.520341		
V4	1.754563	0.220998		
V5	1.341245	0.218867		
**Fig 3B. UbiC promoter**				
NT	1.016184	0.095333		
V1	2.005998	0.356655		
V2	5.632153	0.683028		
V3	1.295108	0.118626		
V4	1.313374	0.16513		
V5	2.787428	0.229604		

(n=3)	Mean								S.D.								Statistical method used
Log (SDF1α)M	-6.60	-7.08	-7.56	-8.03	-8.51	-8.99	-9.47	-10.0	-6.60	-7.08	-7.56	-8.03	-8.51	-8.99	-9.47	-10.0	unpaired Student's t-test
**Fig 3C. CMV promoter**																	
V1	2.216	1.907	1.289	1.322	1.320	1.070	0.932	0.955	0.294	0.302	0.270	0.125	0.095	0.086	0.072	0.038	
V2	6.021	5.516	4.656	2.296	1.017	0.950	0.850	0.948	0.381	0.283	0.200	0.091	0.094	0.105	0.045	0.087	
V3	5.878	5.297	5.250	2.849	1.196	0.924	1.022	0.925	0.259	0.404	0.000	0.513	0.140	0.063	0.113	0.055	
V4	2.139	2.745	1.709	0.929	0.866	0.891	0.926	0.918	0.387	0.379	0.207	0.061	0.070	0.018	0.010	0.044	
V5	2.989	2.787	1.417	0.909	1.002	0.959	0.906	0.925	0.863	0.480	0.245	0.015	0.022	0.041	0.039	0.055	
**Fig 3C. UbiC promoter**																	
V1	4.460	3.686	3.370	1.503	0.995	0.950	0.889	0.851	0.430	0.455	0.553	0.175	0.025	0.016	0.015	0.007	
V2	5.754	5.707	5.341	3.858	1.365	1.012	0.894	0.902	0.073	0.276	0.144	0.218	0.218	0.026	0.059	0.088	
V3	1.973	2.273	1.614	1.519	1.163	1.112	0.859	0.885	0.188	0.170	0.227	0.192	0.171	0.160	0.007	0.055	
V4	1.400	1.733	1.420	1.380	0.988	0.907	0.926	0.885	0.175	0.407	0.211	0.160	0.029	0.022	0.074	0.055	
V5	4.931	4.237	3.990	2.336	1.574	0.957	0.887	0.898	0.489	0.149	0.214	0.320	0.072	0.009	0.025	0.019	

(n=3)	Mean		S.D.		Statistical method used
**Fig 3E. CMV/CXCR4**	SDF1α	Veh.	SDF1α	Veh.	unpaired Student's t-test
Vec.	1.01426	1	0.09956	0.003783	
V1	1.20649	1	0.12752	0.053403	
V2	3.041045	1	0.203288	0.055751	
V3	1.314329	1	0.111368	0.049351	
V4	1.03853	1	0.125278	0.104257	
V5	1.244379	1	0.173493	0.127407	
**Fig 3E. UbiC/CXCR4**					
Vec.	0.954187	1	0.097956	0.279126	
V1	0.955091	1	0.225892	0.194826	
V2	5.154798	1	0.355618	0.097119	

V3	1.177829	1	0.061036	0.106196	
V4	1.045337	1	0.005025	0.115751	
V5	1.109883	1	0.003432	0.071322	

**Fig 4A. V1**					
(n=3)	Mean		S.D.		Statistical method used
Time(min)	SDF1α	Veh.	SDF1α	Veh.	unpaired Student's t-test
0	0.920706	0.944134	0.080079	0.037408	
0.75	0.966793	0.991786	0.075065	0.01866	
1.5	1.018167	1.057063	0.073247	0.015112	
2.25	1.046408	1.090239	0.061288	0.017474	
3	1.063587	1.110365	0.036824	0.011503	
3.75	1.084398	1.090747	0.036274	0.002856	
4.5	1.084288	1.083507	0.026948	0.016173	
5.25	1.078241	1.075519	0.043058	0.014157	
6	1.065615	1.074984	0.020925	0.02856	
6.75	1.042945	1.058366	0.006847	0.021271	
7.5	1.025273	1.041395	0.005651	0.029503	
8.25	1.021942	1.013646	0.004208	0.018067	
9	1	1	0	0	
9.75	1.234369	0.908543	0.061364	0.025305	
10.5	1.451894	0.864947	0.120782	0.031428	
11.25	1.501154	0.837423	0.125844	0.030599	
12	1.439096	0.830809	0.125789	0.026128	
12.75	1.387806	0.819508	0.127873	0.016977	
13.5	1.308961	0.812964	0.102999	0.019925	
14.25	1.233796	0.804861	0.102121	0.017398	
15	1.169619	0.789249	0.091786	0.021852	
15.75	1.109249	0.780714	0.076812	0.024418	
16.5	1.051945	0.783168	0.085548	0.01341	
17.25	1.000943	0.753212	0.059159	0.027424	
18	0.970549	0.749252	0.048047	0.023512	
18.75	0.948225	0.738452	0.062674	0.020594	
19.5	0.908716	0.721626	0.043132	0.018067	
20.25	0.89365	0.709488	0.047897	0.030151	
21	0.849865	0.706967	0.042593	0.017125	
21.75	0.852821	0.694474	0.040619	0.01073	
22.5	0.811187	0.678108	0.027348	0.011121	
23.25	0.79275	0.670092	0.037463	0.01785	
24	0.767426	0.662935	0.045239	0.012996	
24.75	0.754487	0.643112	0.025947	0.018806	
25.5	0.744859	0.629851	0.030425	0.003322	
26.25	0.720738	0.62547	0.028608	0.016341	
27	0.719851	0.615635	0.02682	0.02521	
27.75	0.703084	0.601158	0.032222	0.016463	
28.5	0.680476	0.59964	0.025679	0.010269	
29.25	0.676766	0.583351	0.035597	0.017033	
30	0.669932	0.57779	0.027644	0.021744	
30.75	0.64715	0.568034	0.031413	0.011791	
31.5	0.640563	0.552485	0.029993	0.015644	
32.25	0.6287	0.543965	0.021878	0.01936	
33	0.616866	0.536634	0.017419	0.022652	
33.75	0.607832	0.531254	0.027975	0.022545	
34.5	0.595062	0.522928	0.022273	0.021437	
35.25	0.586174	0.514387	0.02403	0.021059	
36	0.57984	0.502821	0.034254	0.019519	
36.75	0.569112	0.498515	0.026562	0.004761	
37.5	0.556969	0.486371	0.021121	0.02734	
38.25	0.54917	0.479872	0.017067	0.023126	
39	0.541904	0.466918	0.023287	0.019376	
39.75	0.531714	0.465292	0.020576	0.018808	
40.5	0.514861	0.459592	0.028725	0.019869	
41.25	0.513322	0.450566	0.026005	0.023391	
42	0.5036	0.438212	0.021959	0.018562	
42.75	0.501318	0.428276	0.021225	0.009495	

43.5	0.490181	0.425435	0.025963	0.014276	
44.25	0.478002	0.415773	0.019159	0.021744	
45	0.471904	0.412114	0.020387	0.01372	
45.75	0.471186	0.401765	0.031516	0.017696	
46.5	0.453577	0.393518	0.024082	0.019889	
47.25	0.449259	0.38646	0.023752	0.016966	
48	0.445928	0.387752	0.034169	0.011853	
48.75	0.437448	0.377194	0.021298	0.01899	
49.5	0.427623	0.37209	0.027467	0.021834	
50.25	0.423923	0.365751	0.015471	0.015059	
51	0.411161	0.360792	0.021114	0.01798	
51.75	0.407077	0.356346	0.023488	0.015142	
52.5	0.407859	0.342056	0.027771	0.015694	
53.25	0.395116	0.334325	0.019189	0.012766	
54	0.38797	0.329546	0.019894	0.014113	
54.75	0.384589	0.326164	0.026827	0.015599	
55.5	0.377248	0.316809	0.031364	0.017428	
56.25	0.368215	0.312027	0.015032	0.019935	
57	0.363779	0.301714	0.024735	0.012325	
57.75	0.36191	0.301396	0.023657	0.014309	
58.5	0.350715	0.299993	0.024028	0.021036	
59.25	0.34717	0.294349	0.028474	0.017343	
60	0.338929	0.289094	0.021816	0.011186	
60.75	0.334737	0.283429	0.022642	0.013501	
61.5	0.323773	0.269025	0.017351	0.013166	
62.25	0.321132	0.269762	0.027611	0.017188	
63	0.316699	0.263471	0.026269	0.020368	
63.75	0.312685	0.261328	0.033446	0.015884	
64.5	0.308961	0.257109	0.028579	0.018116	
65.25	0.306678	0.246452	0.024073	0.017011	
66	0.297818	0.239612	0.02557	0.009647	
66.75	0.291257	0.239857	0.017566	0.010189	
67.5	0.292642	0.234951	0.021368	0.016833	
68.25	0.285646	0.231753	0.027374	0.011005	
69	0.283431	0.223756	0.026909	0.01318	
69.75	0.271963	0.222683	0.02959	0.013287	

Fig 4A. V2

(n=3)	Mean		S.D.		Statistical method used
Time(min)	SDF1α	Veh.	SDF1α	Veh.	unpaired Student's t-test
0	0.911862	0.869353	0.029227	0.048808	
0.75	0.948894	0.916641	0.027697	0.023471	
1.5	0.99482	0.982252	0.034273	0.02799	
2.25	1.031423	1.019649	0.0352	0.030998	
3	1.05147	1.043129	0.045057	0.022824	
3.75	1.051508	1.05853	0.03856	0.01024	
4.5	1.062826	1.060413	0.027114	0.02172	
5.25	1.058545	1.057869	0.027363	0.014856	
6	1.045578	1.053769	0.015351	0.008084	
6.75	1.039292	1.04435	0.017388	0.013897	
7.5	1.026081	1.034038	0.008868	0.007438	
8.25	1.012007	1.022045	0.005973	0.002036	
9	1	1	0	0	
9.75	1.730475	0.885496	0.104302	0.00805	
10.5	2.258851	0.848147	0.205164	0.01264	
11.25	2.533853	0.831813	0.271679	0.012815	
12	2.648312	0.820775	0.305588	0.00342	
12.75	2.657114	0.818578	0.306187	0.005213	
13.5	2.641357	0.804847	0.292579	0.005547	
14.25	2.588769	0.808177	0.275299	0.002423	
15	2.519801	0.792972	0.247592	0.000519	
15.75	2.444507	0.783985	0.230226	0.005524	
16.5	2.365837	0.773361	0.216662	0.006793	
17.25	2.281807	0.770019	0.218714	0.004565	
18	2.194044	0.757759	0.191759	0.004219	
18.75	2.122458	0.740663	0.177628	0.022981	

19.5	2.056152	0.731682	0.182116	0.006153	
20.25	1.975178	0.723903	0.156276	0.010254	
21	1.901938	0.717841	0.15722	0.004745	
21.75	1.835477	0.703177	0.160934	0.00594	
22.5	1.778652	0.696074	0.149915	0.007934	
23.25	1.720145	0.681517	0.149899	0.012826	
24	1.654945	0.667611	0.130132	0.008358	
24.75	1.613447	0.666648	0.125681	0.013986	
25.5	1.563156	0.647569	0.130027	0.01104	
26.25	1.519628	0.634744	0.118638	0.00634	
27	1.47258	0.627688	0.12814	0.001248	
27.75	1.422022	0.614485	0.115	0.01095	
28.5	1.386731	0.615055	0.11196	0.010303	
29.25	1.344955	0.60147	0.128495	0.009707	
30	1.314122	0.59445	0.103606	0.012858	
30.75	1.278889	0.581429	0.114555	0.012875	
31.5	1.240905	0.57106	0.089195	0.016875	
32.25	1.215355	0.559892	0.088565	0.00646	
33	1.190665	0.556999	0.094016	0.004294	
33.75	1.161989	0.546191	0.096956	0.009152	
34.5	1.133544	0.531727	0.084974	0.015061	
35.25	1.107514	0.530735	0.087973	0.021448	
36	1.074108	0.520016	0.083773	0.007994	
36.75	1.057902	0.510478	0.080614	0.005464	
37.5	1.028687	0.501791	0.080323	0.014827	
38.25	1.008565	0.49873	0.066091	0.008348	
39	0.984577	0.489614	0.072481	0.011723	
39.75	0.96615	0.484266	0.063167	0.009946	
40.5	0.956861	0.475372	0.070223	0.010382	
41.25	0.940873	0.466326	0.067456	0.007894	
42	0.916983	0.461654	0.056934	0.006775	
42.75	0.89062	0.454597	0.063877	0.004045	
43.5	0.878093	0.442954	0.058472	0.007234	
44.25	0.856928	0.434133	0.050353	0.009279	
45	0.845554	0.426243	0.057011	0.007923	
45.75	0.8229	0.418342	0.056096	0.007814	
46.5	0.803622	0.409814	0.049436	0.003507	
47.25	0.776621	0.405513	0.038726	0.00844	
48	0.76833	0.403042	0.046827	0.010974	
48.75	0.74816	0.396484	0.040832	0.014122	
49.5	0.735569	0.392933	0.049404	0.00578	
50.25	0.71536	0.385388	0.037007	0.010508	
51	0.703602	0.371696	0.037855	0.014018	
51.75	0.693526	0.36527	0.043278	0.011436	
52.5	0.674121	0.365263	0.040677	0.011892	
53.25	0.660781	0.3534	0.032887	0.007678	
54	0.655149	0.349778	0.034991	0.012847	
54.75	0.635447	0.342283	0.032542	0.007057	
55.5	0.625235	0.342158	0.031776	0.012411	
56.25	0.618932	0.336976	0.035393	0.00898	
57	0.60266	0.325864	0.035699	0.013048	
57.75	0.589394	0.321627	0.031288	0.012871	
58.5	0.580422	0.317996	0.033215	0.014499	
59.25	0.565047	0.31295	0.036851	0.0149	
60	0.554834	0.301129	0.039256	0.009508	
60.75	0.54796	0.299047	0.028609	0.015285	
61.5	0.53366	0.29503	0.034583	0.015013	
62.25	0.525118	0.287137	0.032776	0.014918	
63	0.515639	0.285242	0.030925	0.015087	
63.75	0.503153	0.278429	0.039246	0.010353	
64.5	0.492637	0.274302	0.025885	0.019013	
65.25	0.48524	0.269171	0.027387	0.01353	
66	0.475575	0.263002	0.032994	0.007422	
66.75	0.469792	0.256741	0.033626	0.014012	
67.5	0.461702	0.254718	0.02853	0.008426	

68.25	0.444592	0.250322	0.035052	0.018003	
69	0.442929	0.241693	0.033396	0.01395	
69.75	0.433326	0.23729	0.028112	0.014863	
**Fig 4A. V3**					
(n=3)	Mean		S.D.		Statistical method used
Time(min)	SDF1α	Veh.	SDF1α	Veh.	unpaired Student's t-test
0	1.072793	1.077417	0.059855	0.036691	
0.75	1.079417	1.121031	0.028753	0.013499	
1.5	1.086387	1.158707	0.040817	0.019472	
2.25	1.104421	1.173348	0.031047	0.017154	
3	1.099609	1.153421	0.030306	0.021806	
3.75	1.102034	1.150136	0.027219	0.0164	
4.5	1.074422	1.134657	0.00933	0.015871	
5.25	1.090226	1.093609	0.026332	0.012786	
6	1.068203	1.094954	0.00873	0.014074	
6.75	1.040262	1.062448	0.009164	0.016762	
7.5	1.029972	1.038284	0.006866	0.004748	
8.25	1.009644	1.02703	0.024878	0.013753	
9	1	1	0	0	
9.75	0.902875	0.903384	0.029014	0.03303	
10.5	0.862357	0.880378	0.005242	0.018134	
11.25	0.843156	0.868446	0.026853	0.015993	
12	0.829228	0.849332	0.032855	0.009097	
12.75	0.815951	0.842097	0.022618	0.010088	
13.5	0.822067	0.81589	0.024431	0.01262	
14.25	0.807263	0.820644	0.015417	0.008914	
15	0.801831	0.795495	0.019393	0.016661	
15.75	0.781833	0.797426	0.022485	0.016717	
16.5	0.770572	0.783297	0.03002	0.002506	
17.25	0.762214	0.763484	0.028885	0.010631	
18	0.754156	0.762702	0.024439	0.012903	
18.75	0.744356	0.74563	0.022119	0.020244	
19.5	0.744003	0.734132	0.013536	0.020925	
20.25	0.725728	0.724774	0.033683	0.025293	
21	0.711422	0.69409	0.027927	0.018051	
21.75	0.706468	0.710199	0.025516	0.017625	
22.5	0.700886	0.67955	0.028098	0.028166	
23.25	0.686178	0.670546	0.030402	0.021162	
24	0.671984	0.662713	0.026174	0.030361	
24.75	0.665081	0.64893	0.013924	0.018834	
25.5	0.655042	0.630796	0.039053	0.015708	
26.25	0.644688	0.626488	0.025117	0.022905	
27	0.641995	0.621089	0.039059	0.036017	
27.75	0.62212	0.61329	0.028492	0.034165	
28.5	0.619826	0.602196	0.052085	0.023387	
29.25	0.610087	0.592057	0.038891	0.017857	
30	0.599218	0.580591	0.029222	0.025884	
30.75	0.582604	0.580453	0.02781	0.026487	
31.5	0.578622	0.563283	0.023194	0.028513	
32.25	0.574661	0.560391	0.034862	0.023587	
33	0.569513	0.562226	0.036765	0.026706	
33.75	0.567233	0.536949	0.037982	0.015196	
34.5	0.561318	0.535818	0.023664	0.018728	
35.25	0.554499	0.518412	0.026472	0.034322	
36	0.543762	0.515576	0.028081	0.02697	
36.75	0.53167	0.51003	0.033198	0.024972	
37.5	0.532408	0.504106	0.037447	0.031693	
38.25	0.509978	0.490246	0.028104	0.025093	
39	0.519993	0.483769	0.041455	0.032384	
39.75	0.506524	0.474708	0.03506	0.027575	
40.5	0.501843	0.464444	0.024277	0.028124	
41.25	0.492066	0.468071	0.046872	0.031329	
42	0.482042	0.448937	0.026868	0.025276	
42.75	0.485615	0.442066	0.037412	0.024653	
43.5	0.478744	0.436733	0.054342	0.02788	

44.25	0.472588	0.423511	0.045832	0.026445	
45	0.457178	0.423567	0.039988	0.018507	
45.75	0.447321	0.416398	0.05274	0.030182	
46.5	0.455962	0.399751	0.044364	0.020855	
47.25	0.449812	0.396421	0.040157	0.035267	
48	0.439126	0.390404	0.050641	0.023139	
48.75	0.434726	0.386027	0.034427	0.022913	
49.5	0.424667	0.379642	0.029197	0.04004	
50.25	0.416618	0.371367	0.042039	0.038337	
51	0.427047	0.355895	0.041196	0.025865	
51.75	0.405189	0.359948	0.031321	0.033726	
52.5	0.39028	0.355145	0.03085	0.035156	
53.25	0.384099	0.343217	0.035705	0.026521	
54	0.385505	0.333306	0.040931	0.039	
54.75	0.384308	0.333167	0.047526	0.035315	
55.5	0.373541	0.31431	0.039927	0.030018	
56.25	0.372522	0.325687	0.039508	0.024461	
57	0.368929	0.316312	0.038782	0.029984	
57.75	0.360728	0.315667	0.042856	0.026147	
58.5	0.362935	0.300181	0.039122	0.027422	
59.25	0.347591	0.300221	0.03476	0.033816	
60	0.346034	0.290478	0.045638	0.027362	
60.75	0.338262	0.2846	0.039661	0.033732	
61.5	0.336613	0.282241	0.036158	0.035237	
62.25	0.334701	0.27471	0.050635	0.029098	
63	0.330173	0.26678	0.038417	0.033761	
63.75	0.320351	0.266561	0.044661	0.033029	
64.5	0.317988	0.260171	0.048088	0.031441	
65.25	0.311111	0.260474	0.036531	0.035424	
66	0.308553	0.251744	0.044917	0.028908	
66.75	0.30121	0.240035	0.042499	0.031235	
67.5	0.297458	0.243695	0.038001	0.040049	
68.25	0.299176	0.231522	0.045283	0.033163	
69	0.287447	0.230842	0.052707	0.037229	
69.75	0.283326	0.220035	0.04389	0.032837	
**Fig 4A. V4**					
(n=3)	Mean		S.D.		Statistical method used
Time(min)	SDF1α	Veh.	SDF1α	Veh.	unpaired Student's t-test
0	1.00268	1.032296	0.05837	0.052154	
0.75	1.02026	1.051285	0.039188	0.041401	
1.5	1.056435	1.086054	0.042406	0.042317	
2.25	1.067499	1.109818	0.037467	0.029929	
3	1.076931	1.113449	0.029092	0.010858	
3.75	1.059047	1.112079	0.038177	0.001121	
4.5	1.084492	1.083805	0.036245	0.034863	
5.25	1.04848	1.075103	0.036686	0.022446	
6	1.056063	1.063721	0.030806	0.003671	
6.75	1.056016	1.054032	0.019989	0.021888	
7.5	1.022348	1.040668	0.021537	0.021333	
8.25	1.026718	1.016852	0.03411	0.020721	
9	1	1	0	0	
9.75	0.924595	0.907627	0.019616	0.009117	
10.5	0.895132	0.89134	0.002251	0.020873	
11.25	0.877233	0.879931	0.016114	0.008241	
12	0.866361	0.861722	0.017075	0.014665	
12.75	0.856048	0.854534	0.004675	0.007796	
13.5	0.843374	0.841823	0.01747	0.015623	
14.25	0.841576	0.817777	0.006045	0.007576	
15	0.826898	0.818953	0.009751	0.008739	
15.75	0.818181	0.813582	0.000964	0.010886	
16.5	0.79697	0.796125	0.016368	0.009963	
17.25	0.784566	0.788391	0.00634	0.018284	
18	0.772711	0.776181	0.009927	0.018023	
18.75	0.762113	0.758897	0.008342	0.009535	
19.5	0.7571	0.748197	0.010409	0.004705	

20.25	0.750104	0.737502	0.008783	0.01038	
21	0.744538	0.733778	0.009703	0.010266	
21.75	0.720592	0.728965	0.00855	0.012314	
22.5	0.708259	0.707661	0.013984	0.014004	
23.25	0.713266	0.699196	0.018835	0.007977	
24	0.704553	0.695835	0.004483	0.008004	
24.75	0.687384	0.678288	0.011134	0.00459	
25.5	0.680123	0.658244	0.009772	0.002871	
26.25	0.66429	0.648018	0.00488	0.012534	
27	0.647557	0.64768	0.007023	0.015062	
27.75	0.658032	0.630547	0.007697	0.012258	
28.5	0.643146	0.634118	0.002227	0.016214	
29.25	0.625014	0.611329	0.016299	0.021208	
30	0.616471	0.620712	0.009919	0.013672	
30.75	0.622416	0.608695	0.019772	0.005971	
31.5	0.608361	0.588306	0.028067	0.016306	
32.25	0.606805	0.580503	0.00599	0.011349	
33	0.592713	0.572297	0.007471	0.010137	
33.75	0.575826	0.55757	0.025355	0.01242	
34.5	0.571731	0.553763	0.013863	0.0043	
35.25	0.56661	0.550069	0.005572	0.010362	
36	0.561317	0.538597	0.014333	0.01354	
36.75	0.547627	0.538945	0.007363	0.009779	
37.5	0.553328	0.528185	0.01166	0.011184	
38.25	0.539709	0.516547	0.013355	0.004543	
39	0.531243	0.514132	0.013852	0.004958	
39.75	0.527883	0.501665	0.013671	0.002613	
40.5	0.523211	0.499393	0.011664	0.00702	
41.25	0.526103	0.486549	0.019639	0.003221	
42	0.505837	0.471303	0.009699	0.013255	
42.75	0.499356	0.473069	0.014627	0.004591	
43.5	0.499496	0.472306	0.003119	0.004685	
44.25	0.489994	0.46241	0.01604	0.012456	
45	0.484357	0.444989	0.004462	0.014224	
45.75	0.465806	0.444443	0.006225	0.010225	
46.5	0.457996	0.436238	0.022732	0.021079	
47.25	0.465978	0.430952	0.015916	0.009799	
48	0.455555	0.418247	0.013315	0.010019	
48.75	0.446986	0.410712	0.012469	0.016756	
49.5	0.440411	0.403637	0.017901	0.008752	
50.25	0.436976	0.406861	0.013108	0.006719	
51	0.438281	0.398739	0.013587	0.020777	
51.75	0.429061	0.390164	0.02579	0.004185	
52.5	0.424302	0.385324	0.023332	0.00969	
53.25	0.413239	0.372841	0.016761	0.011252	
54	0.413993	0.369874	0.010959	0.011174	
54.75	0.404001	0.369244	0.012906	0.009282	
55.5	0.394508	0.358259	0.008396	0.009403	
56.25	0.395785	0.353604	0.007952	0.006111	
57	0.3821	0.347617	0.009504	0.013724	
57.75	0.377132	0.344152	0.016517	0.015106	
58.5	0.391348	0.340065	0.019711	0.010855	
59.25	0.371899	0.332622	0.02145	0.010308	
60	0.36284	0.329462	0.017839	0.013539	
60.75	0.352699	0.32695	0.010558	0.012579	
61.5	0.356939	0.310355	0.026712	0.019065	
62.25	0.35522	0.309439	0.014697	0.000585	
63	0.342576	0.303564	0.013937	0.011227	
63.75	0.350373	0.299674	0.01351	0.009829	
64.5	0.346035	0.298941	0.02133	0.007227	
65.25	0.335502	0.293354	0.012777	0.008513	
66	0.326875	0.282553	0.017323	0.014113	
66.75	0.32246	0.271849	0.019944	0.01133	
67.5	0.317166	0.27731	0.021195	0.011047	
68.25	0.310333	0.273055	0.016098	0.004238	

69	0.306708	0.26295	0.021967	0.013771	
69.75	0.297823	0.25448	0.014304	0.006109	
**Fig 4A. V5**					
(n=3)	Mean		S.D.		Statistical method used
Time(min)	SDF1α	Veh.	SDF1α	Veh.	unpaired Student's t-test
0	0.96639	0.968243	0.02692	0.019466	
0.75	1.011147	1.008772	0.015483	0.025594	
1.5	1.043196	1.04306	0.027078	0.01264	
2.25	1.071571	1.050785	0.012601	0.007961	
3	1.066401	1.072922	0.027408	0.005301	
3.75	1.076032	1.073921	0.015318	0.00931	
4.5	1.059648	1.063951	0.016932	0.012231	
5.25	1.053905	1.072725	0.010212	0.002406	
6	1.050007	1.046534	0.027616	0.015979	
6.75	1.027085	1.035169	0.019458	0.004016	
7.5	1.017444	1.02534	0.00842	0.005326	
8.25	1.013251	1.010791	0.007541	0.014924	
9	1	1	0	0	
9.75	1.494333	0.892551	0.051375	0.0176	
10.5	1.501359	0.887468	0.06341	0.015398	
11.25	1.42239	0.866751	0.059052	0.016379	
12	1.327983	0.84569	0.063172	0.015288	
12.75	1.256037	0.843149	0.063154	0.006717	
13.5	1.186864	0.831251	0.058873	0.012843	
14.25	1.108853	0.819842	0.051964	0.020218	
15	1.073665	0.80829	0.0367	0.003699	
15.75	1.030798	0.801009	0.038577	0.004909	
16.5	0.994799	0.786891	0.028827	0.014122	
17.25	0.958804	0.777195	0.03228	0.005393	
18	0.924967	0.756664	0.030541	0.006793	
18.75	0.903891	0.747055	0.027159	0.006096	
19.5	0.879988	0.737625	0.028854	0.008649	
20.25	0.867026	0.727113	0.029385	0.012433	
21	0.838547	0.719893	0.026609	0.005753	
21.75	0.823179	0.70662	0.019237	0.00677	
22.5	0.804577	0.699284	0.031371	0.017767	
23.25	0.790529	0.686569	0.026323	0.000593	
24	0.766823	0.674381	0.034583	0.006048	
24.75	0.754282	0.665856	0.031696	0.005221	
25.5	0.746007	0.659784	0.031801	0.00485	
26.25	0.729679	0.646775	0.016273	0.006243	
27	0.719062	0.633813	0.032641	0.008687	
27.75	0.709813	0.622918	0.041598	0.003168	
28.5	0.691649	0.613622	0.025424	0.009506	
29.25	0.684969	0.614787	0.026672	0.01205	
30	0.663964	0.599779	0.035675	0.014882	
30.75	0.65527	0.587138	0.02792	0.004754	
31.5	0.648891	0.586075	0.029145	0.011428	
32.25	0.6359	0.577897	0.028023	0.013822	
33	0.628708	0.572788	0.028428	0.013492	
33.75	0.612977	0.55705	0.029465	0.009084	
34.5	0.608222	0.544834	0.029963	0.000347	
35.25	0.595116	0.53537	0.024118	0.012617	
36	0.594439	0.531888	0.026736	0.017264	
36.75	0.580597	0.523509	0.026818	0.012971	
37.5	0.566079	0.514099	0.032979	0.013765	
38.25	0.563724	0.508215	0.029058	0.005455	
39	0.550345	0.498717	0.025451	0.006805	
39.75	0.539247	0.499886	0.024523	0.007099	
40.5	0.541214	0.487546	0.031761	0.005126	
41.25	0.524489	0.477029	0.028109	0.010585	
42	0.525537	0.471716	0.021946	0.004923	
42.75	0.513906	0.464049	0.025895	0.009018	
43.5	0.505951	0.452701	0.029684	0.006018	
44.25	0.494218	0.455352	0.026237	0.017205	

45	0.494066	0.440115	0.034511	0.002133	
45.75	0.482784	0.431799	0.034174	0.004257	
46.5	0.47415	0.426719	0.031572	0.014174	
47.25	0.468202	0.424776	0.031119	0.008519	
48	0.461805	0.421605	0.033027	0.008448	
48.75	0.453152	0.414358	0.033421	0.012019	
49.5	0.447396	0.402502	0.031293	0.006932	
50.25	0.43538	0.393864	0.030145	0.010531	
51	0.427711	0.392803	0.033127	0.008959	
51.75	0.422379	0.377219	0.022786	0.013003	
52.5	0.417591	0.379546	0.028658	0.008529	
53.25	0.413015	0.371932	0.031876	0.004906	
54	0.404003	0.361144	0.031728	0.005861	
54.75	0.396324	0.364924	0.025656	0.008176	
55.5	0.395335	0.35378	0.030439	0.003273	
56.25	0.382429	0.349638	0.0285	0.011774	
57	0.376995	0.344465	0.025139	0.006003	
57.75	0.377326	0.338756	0.027441	0.010647	
58.5	0.367363	0.332052	0.035858	0.004267	
59.25	0.36511	0.331431	0.026735	0.008704	
60	0.357909	0.320056	0.026282	0.015687	
60.75	0.352242	0.322703	0.03376	0.010729	
61.5	0.347075	0.315619	0.028035	0.008641	
62.25	0.338557	0.312016	0.028908	0.014348	
63	0.332986	0.303096	0.029739	0.012895	
63.75	0.328078	0.294347	0.035508	0.005097	
64.5	0.323113	0.296138	0.030358	0.012678	
65.25	0.317545	0.283325	0.032953	0.008212	
66	0.311393	0.286372	0.036268	0.011214	
66.75	0.306866	0.282352	0.02198	0.007795	
67.5	0.302267	0.276137	0.027001	0.007343	
68.25	0.294053	0.270882	0.022187	0.010027	
69	0.291838	0.266767	0.033153	0.010053	
69.75	0.285636	0.26116	0.032464	0.014832	

**Fig 4B. V1**					
(n=3)	Mean		S.D.		Statistical method used
Time(min)	SDF1α	Veh.	SDF1α	Veh.	unpaired Student's t-test
0	1.003581	1.007202	0.009854	0.10665	
0.75	1.154699	0.961656	0.181539	0.107289	
1.5	0.908479	1.06027	0.08962	0.172956	
2.25	1.102599	0.971923	0.123126	0.117298	
3	1.042645	1.089542	0.079165	0.169213	
3.75	0.925115	1.094851	0.075655	0.204319	
4.5	0.968393	0.912956	0.058823	0.076699	
5.25	1.15522	0.990467	0.090497	0.034785	
6	1.062295	0.927526	0.038827	0.035036	
6.75	0.952522	1.095692	0.161159	0.183424	
7.5	0.958321	1.087386	0.096769	0.103824	
8.25	0.94128	0.936212	0.163331	0.087146	
9	0.887442	1.010048	0.1378	0.112564	
9.75	0.937408	0.854272	0.071193	0.172246	
11.2	1.451926	0.875718	0.227499	0.158908	
11.95	1.577168	0.857825	0.051396	0.156882	
12.7	1.4307	0.753736	0.318936	0.043559	
13.45	1.265162	0.78585	0.152604	0.053195	
14.2	1.46267	0.829875	0.12845	0.042399	
14.95	1.33018	0.816326	0.110365	0.144281	
15.7	1.171425	0.78204	0.046562	0.112976	
16.45	1.330393	0.780048	0.123305	0.171021	
17.2	1.106408	0.760625	0.146587	0.100009	
17.95	1.184635	0.708055	0.150977	0.060915	
18.7	0.992527	0.728746	0.097861	0.136948	
19.45	1.04933	0.670299	0.118442	0.11467	

20.2	1.036371	0.731869	0.048318	0.016288	
20.95	1.144141	0.870371	0.00398	0.200063	
21.7	1.049346	0.68009	0.103459	0.06374	
22.45	1.176105	0.638692	0.079062	0.012412	
23.2	1.01402	0.660631	0.10673	0.073356	
23.95	0.977328	0.618253	0.148261	0.113942	
24.7	1.098099	0.715439	0.02904	0.089388	
25.45	1.011186	0.64518	0.088168	0.059415	
26.2	0.952565	0.619259	0.16213	0.042616	
26.95	0.982546	0.74006	0.24738	0.091653	
27.7	1.039643	0.678853	0.258504	0.136228	
28.45	1.016158	0.623254	0.212726	0.060125	
29.2	0.966757	0.697792	0.06016	0.222071	
29.95	0.872775	0.548981	0.118582	0.079233	
30.7	0.78523	0.625452	0.288861	0.090408	
31.45	0.935427	0.599168	0.142139	0.019039	
32.2	0.926041	0.6859	0.057051	0.119896	
32.95	0.948276	0.600588	0.155829	0.101044	
33.7	0.924072	0.652329	0.041417	0.09598	
34.45	0.928993	0.617454	0.184766	0.02853	
35.2	0.884846	0.68877	0.081568	0.239361	
35.95	0.830843	0.608432	0.03346	0.052503	
36.7	0.820224	0.626562	0.160925	0.077478	
37.45	0.843783	0.675943	0.093518	0.106334	
38.2	0.768634	0.673089	0.090901	0.293884	
38.95	0.825354	0.618511	0.185145	0.095076	
39.7	0.765153	0.510091	0.066292	0.046774	
40.45	0.814391	0.522223	0.071908	0.07189	
41.2	0.773034	0.519668	0.124366	0.057706	
41.95	0.885336	0.663783	0.11058	0.095571	
42.7	0.900929	0.64032	0.020064	0.098387	
43.45	0.759999	0.533729	0.134818	0.09131	
44.2	0.775955	0.589622	0.08175	0.131622	
44.95	0.63372	0.584147	0.134786	0.041933	
45.7	0.70929	0.543631	0.19805	0.038139	
46.45	0.691169	0.632083	0.089189	0.065501	
47.2	0.741295	0.472427	0.031805	0.082088	
47.95	0.780469	0.519669	0.082937	0.131754	
48.7	0.758494	0.529056	0.029195	0.05144	
49.45	0.656332	0.522568	0.059099	0.016494	
50.2	0.647272	0.607291	0.026092	0.034076	
50.95	0.622997	0.534566	0.183578	0.080967	
51.7	0.729903	0.522729	0.11497	0.035786	
52.45	0.754343	0.557965	0.089814	0.138867	
53.2	0.664377	0.525833	0.073439	0.105637	
53.95	0.725436	0.633013	0.050714	0.095313	
54.7	0.726915	0.453706	0.187401	0.096437	
55.45	0.641708	0.578754	0.024643	0.118107	
56.2	0.734524	0.508763	0.05973	0.17491	
56.95	0.664978	0.521514	0.034619	0.08873	
57.7	0.602853	0.525423	0.163165	0.074187	
58.45	0.649362	0.49469	0.073573	0.126082	
59.2	0.597695	0.541779	0.138555	0.159066	
59.95	0.619825	0.52691	0.025802	0.043974	
60.7	0.727105	0.483742	0.065438	0.11163	
61.45	0.666426	0.496025	0.116958	0.108511	
62.2	0.645442	0.558919	0.155905	0.183089	
62.95	0.744715	0.557669	0.110512	0.110472	
63.7	0.566231	0.452601	0.11366	0.060072	
64.45	0.547501	0.460679	0.091518	0.14747	
65.2	0.605425	0.583498	0.037675	0.079766	
65.95	0.634153	0.485854	0.095635	0.026225	
66.7	0.491778	0.534668	0.145153	0.125782	
67.45	0.557417	0.433505	0.0448	0.053366	
68.2	0.588548	0.474087	0.073526	0.076935	

68.95	0.533404	0.586133	0.082295	0.062763	
69.7	0.560746	0.52125	0.078434	0.131439	
70.45	0.543354	0.685468	0.151455	0.138738	
71.2	0.550889	0.435258	0.050979	0.060271	
71.95	0.579769	0.489006	0.129358	0.071649	
**Fig 4B. V2**					
(n=3)	Mean		S.D.		Statistical method used
Time(min)	SDF1α	Veh.	SDF1α	Veh.	unpaired Student's t-test
0	1.152383	0.998086	0.097688	0.161782	
0.75	1.132667	1.131582	0.12943	0.233201	
1.5	1.005459	0.862757	0.083861	0.15727	
2.25	1.066481	1.015493	0.023527	0.09789	
3	0.978131	1.048229	0.090465	0.099419	
3.75	0.918875	1.053378	0.046164	0.090897	
4.5	0.980864	1.059568	0.022209	0.130473	
5.25	0.930537	1.044928	0.0438	0.131698	
6	1.091607	1.071931	0.050423	0.059576	
6.75	0.884577	0.929516	0.053429	0.033698	
7.5	1.017565	0.983456	0.107003	0.133843	
8.25	0.922743	0.887927	0.066224	0.078599	
9	1.032964	0.893132	0.105366	0.039963	
9.75	0.885148	1.020018	0.07369	0.133845	
11.2	1.587335	0.826071	0.054061	0.114083	
11.95	2.353784	0.879566	0.059865	0.151636	
12.7	2.185579	0.682579	0.051017	0.021472	
13.45	2.138873	0.732924	0.1131	0.153162	
14.2	2.246807	0.704676	0.156565	0.04781	
14.95	2.067543	0.794137	0.096235	0.075481	
15.7	1.987632	0.759113	0.057636	0.034389	
16.45	1.917452	0.740162	0.087042	0.094475	
17.2	1.899562	0.722249	0.216606	0.056838	
17.95	1.825722	0.74018	0.124159	0.018767	
18.7	1.813557	0.731176	0.191474	0.104339	
19.45	1.620835	0.820393	0.124274	0.118587	
20.2	1.741328	0.843882	0.043968	0.115539	
20.95	1.638735	0.722537	0.126959	0.102169	
21.7	1.616325	0.714765	0.063287	0.039485	
22.45	1.605799	0.649792	0.238847	0.04544	
23.2	1.556483	0.769795	0.083848	0.028328	
23.95	1.504425	0.759012	0.080242	0.087456	
24.7	1.48497	0.683921	0.141008	0.0321	
25.45	1.297584	0.586142	0.134987	0.091408	
26.2	1.409203	0.679121	0.070258	0.086027	
26.95	1.530625	0.617826	0.052515	0.025888	
27.7	1.367016	0.702793	0.03197	0.070933	
28.45	1.441017	0.619248	0.108728	0.071716	
29.2	1.395627	0.68984	0.106748	0.103105	
29.95	1.380103	0.572117	0.056398	0.118663	
30.7	1.241103	0.529081	0.072095	0.038026	
31.45	1.236419	0.566349	0.170558	0.141184	
32.2	1.154372	0.687485	0.06511	0.073162	
32.95	1.007676	0.583098	0.062114	0.128561	
33.7	1.170873	0.566383	0.132385	0.075722	
34.45	1.195663	0.663024	0.072857	0.05405	
35.2	1.071325	0.709554	0.055494	0.120643	
35.95	1.076347	0.700356	0.072428	0.015657	
36.7	1.165625	0.586363	0.123898	0.076468	
37.45	1.105486	0.603814	0.035107	0.064541	
38.2	1.125649	0.606844	0.08331	0.1011	
38.95	1.063669	0.586272	0.13011	0.011388	
39.7	1.066779	0.574496	0.034456	0.146494	
40.45	1.025017	0.58457	0.057995	0.025254	
41.2	1.055054	0.607502	0.013443	0.059772	
41.95	1.074799	0.49755	0.152336	0.036336	
42.7	1.001904	0.595361	0.147935	0.056497	

43.45	0.965999	0.492659	0.076418	0.068623	
44.2	0.933896	0.611171	0.111754	0.072965	
44.95	0.999779	0.624752	0.099675	0.090136	
45.7	0.942004	0.543581	0.067855	0.100543	
46.45	0.913891	0.52694	0.048036	0.013484	
47.2	0.913288	0.541937	0.009688	0.076232	
47.95	0.853382	0.639862	0.167794	0.050037	
48.7	0.817221	0.578679	0.038031	0.03361	
49.45	0.955397	0.555087	0.186341	0.036558	
50.2	0.867101	0.615649	0.069544	0.106534	
50.95	0.900487	0.587675	0.049692	0.105956	
51.7	1.028806	0.604473	0.047056	0.099999	
52.45	0.868692	0.580193	0.122888	0.089196	
53.2	0.814835	0.555315	0.093035	0.074316	
53.95	0.861237	0.535783	0.117878	0.077938	
54.7	0.783827	0.493672	0.035067	0.074498	
55.45	0.775267	0.517535	0.079594	0.022473	
56.2	0.879971	0.585173	0.03848	0.068487	
56.95	0.760625	0.496144	0.058906	0.059009	
57.7	0.734546	0.577667	0.019947	0.0312	
58.45	0.666389	0.541753	0.089024	0.027894	
59.2	0.704933	0.408673	0.071358	0.079368	
59.95	0.745121	0.50068	0.034454	0.101846	
60.7	0.822968	0.485728	0.049731	0.052511	
61.45	0.767435	0.484681	0.11444	0.076812	
62.2	0.771757	0.479549	0.156259	0.055566	
62.95	0.684028	0.473647	0.066853	0.135342	
63.7	0.737802	0.419695	0.05521	0.046366	
64.45	0.754797	0.442536	0.03159	0.025215	
65.2	0.658216	0.46916	0.051753	0.13332	
65.95	0.698949	0.562699	0.071793	0.064436	
66.7	0.728397	0.43253	0.085568	0.071944	
67.45	0.745567	0.487134	0.025838	0.069943	
68.2	0.76843	0.518433	0.061703	0.071971	
68.95	0.671766	0.434518	0.078499	0.059084	
69.7	0.685482	0.508127	0.042044	0.105878	
70.45	0.658882	0.530675	0.056046	0.088297	
71.2	0.665239	0.547053	0.117106	0.054028	
71.95	0.616969	0.568281	0.022781	0.172544	
**Fig 4B. V3**					
(n=3)	Mean		S.D.		Statistical method used
Time(min)	SDF1α	Veh.	SDF1α	Veh.	unpaired Student's t-test
0	1.097287	1.09188	0.18741	0.345267	
0.75	1.177463	0.990465	0.29168	0.226949	
1.5	1.176682	0.992587	0.124025	0.249589	
2.25	1.100601	1.139965	0.174881	0.34427	
3	0.899177	1.090542	0.088443	0.426299	
3.75	1.077053	1.230509	0.321017	0.378264	
4.5	0.879431	0.919524	0.161694	0.398325	
5.25	0.942632	0.990192	0.141374	0.272605	
6	1.043256	0.795038	0.236861	0.276493	
6.75	0.938955	1.012271	0.371012	0.293676	
7.5	0.908668	0.955653	0.177514	0.270937	
8.25	0.809801	0.59051	0.094668	0.050955	
9	0.921509	1.082332	0.037684	0.290867	
9.75	1.027486	1.118532	0.296685	0.094696	
11.2	1.359339	0.948739	0.272077	0.014238	
11.95	0.873724	1.053176	0.055788	0.217432	
12.7	1.176965	0.809767	0.18455	0.073149	
13.45	1.154392	0.989725	0.380668	0.388908	
14.2	1.062097	1.14097	0.064108	0.22594	
14.95	0.802233	1.020187	0.119815	0.105355	
15.7	1.006137	0.923651	0.639637	0.354274	
16.45	0.875686	0.658597	0.226422	0.353042	
17.2	1.193457	0.691845	0.55683	0.344002	

17.95	0.870498	0.50159	0.456045	0.147471	
18.7	0.722436	0.825412	0.223948	0.158165	
19.45	0.950829	0.84025	0.380143	0.272586	
20.2	0.766626	0.900254	0.180262	0.310542	
20.95	0.68782	0.788024	0.024357	0.362831	
21.7	0.671683	0.795802	0.326867	0.342417	
22.45	0.895174	0.774999	0.230873	0.403155	
23.2	0.905875	0.715957	0.249682	0.160018	
23.95	0.943572	0.792113	0.187721	0.273159	
24.7	0.768995	0.77083	0.151344	0.277911	
25.45	0.663175	0.769067	0.117782	0.437341	
26.2	0.667068	0.861818	0.076877	0.321263	
26.95	0.622836	0.652236	0.159647	0.306073	
27.7	0.588917	0.790522	0.078157	0.044581	
28.45	0.562201	0.747538	0.153923	0.195493	
29.2	0.772632	0.947645	0.126001	0.142038	
29.95	0.489207	0.935827	0.178489	0.437764	
30.7	0.785188	0.962945	0.067068	0.121985	
31.45	0.629911	0.579614	0.29875	0.248684	
32.2	0.544314	0.823407	0.296286	0.13775	
32.95	0.757324	0.558126	0.194459	0.210878	
33.7	0.66092	0.598679	0.076256	0.399734	
34.45	0.506433	0.758557	0.153994	0.152967	
35.2	0.803622	0.799458	0.065255	0.278031	
35.95	0.852631	0.754222	0.121701	0.516476	
36.7	0.593898	0.738132	0.109933	0.239725	
37.45	0.830942	0.885647	0.083486	0.189648	
38.2	0.743545	0.543303	0.237651	0.087013	
38.95	0.768165	0.849428	0.196744	0.276209	
39.7	0.683061	0.570086	0.198619	0.153003	
40.45	0.71299	0.42372	0.258379	0.143317	
41.2	0.615317	0.563118	0.159893	0.181706	
41.95	0.639838	0.706885	0.183931	0.231028	
42.7	0.693342	0.55258	0.078486	0.041964	
43.45	0.930878	0.672008	0.465807	0.165077	
44.2	0.583699	0.6657	0.190117	0.227934	
44.95	0.770126	0.474833	0.133719	0.176591	
45.7	0.761483	1.032653	0.326237	0.356205	
46.45	0.740723	0.952559	0.352805	0.387955	
47.2	0.650263	0.77575	0.280105	0.170289	
47.95	0.692397	0.95674	0.103666	0.285934	
48.7	0.626401	0.640606	0.301687	0.420563	
49.45	0.645653	0.495563	0.341258	0.299423	
50.2	0.773314	0.850805	0.314767	0.247801	
50.95	0.607303	0.694689	0.154601	0.169002	
51.7	0.753186	0.607484	0.16372	0.353287	
52.45	0.449816	0.577154	0.107042	0.075386	
53.2	0.550581	0.606647	0.289944	0.390135	
53.95	0.838045	0.756301	0.490747	0.12792	
54.7	0.491957	0.725466	0.181394	0.159682	
55.45	0.569979	0.592173	0.051855	0.059087	
56.2	0.871745	0.564264	0.192739	0.083107	
56.95	0.695796	0.695513	0.131825	0.13886	
57.7	0.832255	0.65306	0.44472	0.344258	
58.45	0.756456	0.701674	0.151284	0.120504	
59.2	0.711027	0.709062	0.239039	0.216506	
59.95	0.721306	0.571852	0.137159	0.252057	
60.7	0.436786	0.426082	0.135004	0.166363	
61.45	0.511062	0.450106	0.254452	0.150372	
62.2	0.746329	0.716778	0.126332	0.084453	
62.95	0.630428	0.563567	0.091491	0.17295	
63.7	0.676607	0.480312	0.16243	0.139541	
64.45	0.541799	0.636285	0.045963	0.347966	
65.2	0.65971	0.811155	0.032753	0.151207	
65.95	0.432769	0.466917	0.161113	0.194428	

66.7	0.717845	0.605354	0.225562	0.125827	
67.45	0.773689	0.453069	0.258121	0.30913	
68.2	0.551194	0.535223	0.148183	0.159214	
68.95	0.483938	0.659822	0.212666	0.074778	
69.7	0.663712	0.652558	0.231342	0.041563	
70.45	0.586321	0.962561	0.065191	0.091144	
71.2	0.636867	0.605202	0.13409	0.063109	
71.95	0.492324	0.839908	0.090325	0.410989	
**Fig 4B. V4**					
(n=3)	Mean		S.D.		Statistical method used
Time(min)	SDF1α	Veh.	SDF1α	Veh.	unpaired Student's t-test
0	1.221732	1.054831	0.100075	0.187309	
0.75	1.011827	1.055819	0.171869	0.295674	
1.5	0.946216	1.127799	0.176254	0.155031	
2.25	1.020308	1.157208	0.171648	0.396128	
3	1.072148	1.134324	0.187075	0.103127	
3.75	1.055059	1.121735	0.036314	0.139105	
4.5	0.969861	0.93571	0.233574	0.097189	
5.25	1.025622	1.103782	0.165513	0.227498	
6	1.155684	0.992876	0.096559	0.026223	
6.75	1.066939	0.91334	0.118255	0.062637	
7.5	0.853632	0.907889	0.222874	0.092043	
8.25	0.850939	0.717577	0.163171	0.166252	
9	0.931371	0.96447	0.194738	0.225582	
9.75	0.818661	0.812642	0.110824	0.258788	
11.2	1.005526	0.891233	0.312589	0.266349	
11.95	0.965754	0.716599	0.047208	0.163447	
12.7	0.814589	0.733118	0.055151	0.151922	
13.45	0.922082	0.789329	0.152899	0.203874	
14.2	0.940322	0.81568	0.059	0.296367	
14.95	0.848169	0.647803	0.148785	0.084854	
15.7	0.76326	0.823591	0.029824	0.244151	
16.45	0.679112	0.889404	0.091335	0.201733	
17.2	0.871068	0.692357	0.032588	0.012551	
17.95	0.724293	0.713753	0.186273	0.071389	
18.7	0.556259	0.811061	0.099444	0.259555	
19.45	0.780315	0.57821	0.168765	0.224204	
20.2	0.836376	0.654449	0.184737	0.083831	
20.95	0.611315	0.754451	0.038799	0.195797	
21.7	0.804286	0.515676	0.221348	0.087669	
22.45	0.745979	0.853637	0.201766	0.09909	
23.2	0.755998	0.599027	0.047342	0.092765	
23.95	0.794483	0.88662	0.305043	0.092722	
24.7	0.706556	0.84888	0.112715	0.122873	
25.45	0.71436	0.674537	0.133161	0.109709	
26.2	0.635002	0.658023	0.14384	0.216988	
26.95	0.619281	0.809496	0.175549	0.075269	
27.7	0.848161	0.532886	0.062796	0.153713	
28.45	0.655879	0.598357	0.233219	0.231081	
29.2	0.672811	0.540397	0.203734	0.092685	
29.95	0.533037	0.68051	0.039921	0.245702	
30.7	0.726127	0.658537	0.120084	0.118738	
31.45	0.615153	0.697995	0.072366	0.184456	
32.2	0.609126	0.535357	0.155353	0.319655	
32.95	0.648493	0.595701	0.182871	0.049106	
33.7	0.577144	0.657752	0.117146	0.185416	
34.45	0.5864	0.717076	0.105266	0.255765	
35.2	0.586568	0.435936	0.120219	0.096372	
35.95	0.569458	0.671946	0.008006	0.146121	
36.7	0.561808	0.527255	0.125925	0.047315	
37.45	0.619967	0.66016	0.181924	0.102397	
38.2	0.59006	0.76854	0.05005	0.09056	
38.95	0.747666	0.630876	0.143292	0.202109	
39.7	0.556331	0.560347	0.136866	0.007886	
40.45	0.612614	0.682889	0.098563	0.092293	

41.2	0.630282	0.64511	0.086412	0.240567	
41.95	0.743174	0.578836	0.203898	0.05819	
42.7	0.426904	0.687002	0.031414	0.185594	
43.45	0.630006	0.758375	0.120953	0.0837	
44.2	0.644736	0.730711	0.089102	0.095517	
44.95	0.495459	0.632197	0.045552	0.18949	
45.7	0.581395	0.622633	0.13253	0.233724	
46.45	0.535494	0.549872	0.041756	0.164684	
47.2	0.624991	0.60395	0.167058	0.113595	
47.95	0.724328	0.540281	0.13785	0.106836	
48.7	0.51087	0.580362	0.147086	0.117249	
49.45	0.638059	0.521478	0.11755	0.080534	
50.2	0.545644	0.613725	0.01052	0.10733	
50.95	0.606011	0.434798	0.027058	0.100303	
51.7	0.581575	0.662591	0.070909	0.186802	
52.45	0.673877	0.59876	0.083253	0.240358	
53.2	0.531407	0.645614	0.040681	0.083234	
53.95	0.649625	0.548794	0.182871	0.274341	
54.7	0.542798	0.439052	0.175143	0.117159	
55.45	0.492696	0.533967	0.049346	0.102442	
56.2	0.48467	0.473577	0.034738	0.054327	
56.95	0.531926	0.529258	0.119222	0.151947	
57.7	0.63769	0.532775	0.13276	0.204755	
58.45	0.560418	0.479161	0.061916	0.107236	
59.2	0.539419	0.522909	0.181851	0.121981	
59.95	0.592032	0.491997	0.051219	0.114193	
60.7	0.487981	0.449927	0.096536	0.139339	
61.45	0.454927	0.399591	0.03802	0.148135	
62.2	0.509716	0.612746	0.079833	0.04598	
62.95	0.476203	0.605596	0.110253	0.06875	
63.7	0.471216	0.547283	0.142515	0.142501	
64.45	0.423309	0.576681	0.157587	0.101296	
65.2	0.46219	0.531903	0.088916	0.04936	
65.95	0.517648	0.579754	0.069379	0.119819	
66.7	0.520455	0.501146	0.099787	0.146923	
67.45	0.517563	0.624015	0.081715	0.104875	
68.2	0.544825	0.7548	0.02092	0.145221	
68.95	0.541101	0.418658	0.097448	0.122997	
69.7	0.543146	0.462862	0.082597	0.107419	
70.45	0.458657	0.502079	0.023886	0.085404	
71.2	0.421578	0.424436	0.016567	0.05578	
71.95	0.44651	0.525954	0.075539	0.101694	
**Fig 4B. V5**					
(n=3)	Mean		S.D.		Statistical method used
Time(min)	SDF1α	Veh.	SDF1α	Veh.	unpaired Student's t-test
0	1.059558	1.111286	0.138289	0.112323	
0.75	0.972767	1.13126	0.11611	0.17683	
1.5	1.123947	1.063108	0.022745	0.042491	
2.25	1.050939	0.963784	0.161605	0.07532	
3	1.205084	0.992066	0.040945	0.111556	
3.75	1.009202	1.071357	0.094337	0.055922	
4.5	0.989201	0.994485	0.180428	0.096407	
5.25	0.947498	0.902173	0.076847	0.177996	
6	1.010642	0.944292	0.092615	0.112062	
6.75	0.99046	0.920876	0.13197	0.052101	
7.5	0.929601	1.058427	0.088875	0.028531	
8.25	0.856371	0.906823	0.046659	0.090211	
9	0.909311	0.997352	0.090996	0.087882	
9.75	0.94542	0.94271	0.121457	0.040602	
11.2	1.448131	0.840468	0.209771	0.066395	
11.95	1.207562	0.942041	0.151774	0.09953	
12.7	1.334981	0.823276	0.185555	0.114102	
13.45	1.284879	0.757495	0.128834	0.055714	
14.2	1.084531	0.769792	0.15333	0.063606	
14.95	1.118762	0.757426	0.414623	0.086124	

15.7	1.031281	0.743095	0.152816	0.074778	
16.45	1.019364	0.633112	0.024195	0.039778	
17.2	1.068442	0.62054	0.115611	0.026065	
17.95	0.984745	0.678816	0.094627	0.088128	
18.7	1.0092	0.814628	0.147696	0.083682	
19.45	0.955126	0.626955	0.061658	0.051669	
20.2	0.933247	0.658178	0.085841	0.049368	
20.95	0.99516	0.65145	0.120992	0.041334	
21.7	0.999039	0.706021	0.123479	0.0925	
22.45	0.941262	0.637592	0.119246	0.081049	
23.2	0.922762	0.677546	0.107077	0.071575	
23.95	0.985858	0.699842	0.244246	0.071111	
24.7	0.850066	0.640275	0.032213	0.083152	
25.45	0.97572	0.663939	0.075292	0.097433	
26.2	0.879134	0.553036	0.193711	0.024641	
26.95	0.761662	0.640642	0.046259	0.120558	
27.7	0.737664	0.674959	0.110698	0.041669	
28.45	0.843942	0.637209	0.156295	0.000795	
29.2	0.755576	0.601372	0.089817	0.114188	
29.95	0.835986	0.613296	0.210013	0.079729	
30.7	0.845456	0.610218	0.162302	0.120359	
31.45	0.795737	0.666786	0.081904	0.032987	
32.2	0.773102	0.663491	0.135425	0.068556	
32.95	0.839023	0.557353	0.086693	0.075637	
33.7	0.71798	0.680314	0.140072	0.120337	
34.45	0.74397	0.616079	0.066226	0.045413	
35.2	0.785184	0.563849	0.081122	0.056347	
35.95	0.75207	0.61842	0.050109	0.027138	
36.7	0.797951	0.642312	0.149527	0.060686	
37.45	0.764604	0.596726	0.0821	0.003708	
38.2	0.690011	0.611708	0.143283	0.092018	
38.95	0.769346	0.728873	0.130177	0.091066	
39.7	0.754858	0.690067	0.048222	0.082828	
40.45	0.801481	0.559392	0.240801	0.068674	
41.2	0.784013	0.583663	0.122545	0.067552	
41.95	0.702821	0.649093	0.151674	0.084353	
42.7	0.75706	0.524925	0.055592	0.071023	
43.45	0.74712	0.581036	0.086939	0.026273	
44.2	0.708015	0.576078	0.066399	0.146479	
44.95	0.725583	0.573425	0.037848	0.081783	
45.7	0.656084	0.605519	0.055299	0.047639	
46.45	0.689889	0.488194	0.059752	0.087385	
47.2	0.571733	0.442276	0.065363	0.088334	
47.95	0.626477	0.544823	0.174738	0.063015	
48.7	0.633557	0.595708	0.046144	0.025101	
49.45	0.673309	0.535907	0.024436	0.129449	
50.2	0.618152	0.524907	0.074613	0.052441	
50.95	0.593584	0.56118	0.130325	0.061109	
51.7	0.586096	0.5079	0.119018	0.170239	
52.45	0.559101	0.616012	0.053005	0.126669	
53.2	0.630439	0.576512	0.161797	0.056616	
53.95	0.556224	0.51885	0.110895	0.098421	
54.7	0.538264	0.558788	0.138582	0.079466	
55.45	0.622454	0.543226	0.073727	0.058676	
56.2	0.596016	0.481852	0.028464	0.072221	
56.95	0.681951	0.538675	0.081823	0.063989	
57.7	0.532225	0.531913	0.12268	0.136676	
58.45	0.527916	0.47724	0.075075	0.050792	
59.2	0.517192	0.401936	0.026704	0.037839	
59.95	0.558919	0.504291	0.152004	0.051814	
60.7	0.588165	0.515377	0.142803	0.075137	
61.45	0.622264	0.430635	0.049468	0.067805	
62.2	0.596815	0.450917	0.113746	0.080164	
62.95	0.504956	0.446456	0.024329	0.099912	
63.7	0.46319	0.409212	0.112685	0.103816	

64.45	0.483482	0.51313	0.066813	0.117134	
65.2	0.494459	0.500677	0.102155	0.03671	
65.95	0.593615	0.492868	0.031751	0.101835	
66.7	0.498397	0.548202	0.032613	0.014649	
67.45	0.550127	0.439385	0.145145	0.064564	
68.2	0.499478	0.409845	0.124565	0.044757	
68.95	0.469384	0.436706	0.02567	0.033685	
69.7	0.492669	0.460995	0.028889	0.124484	
70.45	0.539626	0.422561	0.039154	0.055023	
71.2	0.552135	0.489265	0.081347	0.047809	
71.95	0.536054	0.492212	0.056424	0.101302	

**Fig 4C. V1**					
(n=3)	Mean		S.D.		Statistical method used
Time(min)	SDF1α	Veh.	SDF1α	Veh.	unpaired Student's t-test
0	0.858756	0.928746	0.028777	0.01442	
0.75	0.959163	1.010005	0.021833	0.020742	
1.5	1.005142	1.042397	0.009268	0.007425	
2.25	1.034663	1.06887	0.032348	0.017162	
3	1.043743	1.070771	0.009483	0.006719	
3.75	1.064	1.079686	0.022511	0.008996	
4.5	1.056187	1.062134	0.017757	0.016769	
5.25	1.048364	1.057117	0.025009	0.003424	
6	1.053635	1.047574	0.029795	0.015061	
6.75	1.034663	1.047314	0.0127	0.016505	
7.5	1.027891	1.038002	0.032658	0.020482	
8.25	1.017249	1.024909	0.024729	0.005785	
9	1	1	0	0	
9.75	2.191328	0.769564	0.063321	0.038094	
10.5	2.76617	0.755093	0.042438	0.047384	
11.25	2.922827	0.753559	0.019462	0.045451	
12	2.895464	0.763728	0.021595	0.040728	
12.75	2.794125	0.768494	0.010442	0.031285	
13.5	2.676919	0.778199	0.01824	0.022012	
14.25	2.536998	0.792545	0.021482	0.040025	
15	2.424787	0.788813	0.010866	0.034809	
15.75	2.297433	0.771862	0.018144	0.031788	
16.5	2.190699	0.776196	0.01379	0.013016	
17.25	2.119902	0.782967	0.022363	0.003965	
18	2.010123	0.791824	0.032764	0.010618	
18.75	1.936183	0.766994	0.007008	0.014997	
19.5	1.8888	0.775085	0.018733	0.014087	
20.25	1.811409	0.774433	0.01056	0.012125	
21	1.765894	0.752063	0.004828	0.01306	
21.75	1.703788	0.746691	0.023739	0.019615	
22.5	1.671528	0.746533	0.020956	0.010248	
23.25	1.606874	0.74591	0.01428	0.010965	
24	1.573029	0.737358	0.030012	0.01224	
24.75	1.536727	0.7284	0.033492	0.017831	
25.5	1.503397	0.728293	0.01451	0.012107	
26.25	1.474324	0.722954	0.019686	0.015224	
27	1.436046	0.720369	0.007284	0.0122	
27.75	1.412801	0.725219	0.012171	0.009526	
28.5	1.369917	0.705952	0.013616	0.009965	
29.25	1.360283	0.700987	0.009673	0.01494	
30	1.323316	0.694115	0.029406	0.012882	
30.75	1.307067	0.699725	0.023163	0.011154	
31.5	1.29166	0.704606	0.03149	0.021779	
32.25	1.250796	0.683066	0.015013	0.022744	
33	1.248227	0.680062	0.022948	0.018744	
33.75	1.203946	0.675192	0.022966	0.018203	
34.5	1.211328	0.664825	0.027773	0.006025	
35.25	1.17309	0.661331	0.034183	0.00531	
36	1.15375	0.663091	0.035214	0.002661	
36.75	1.142053	0.645933	0.027767	0.003689	

37.5	1.12629	0.656237	0.023554	0.015576	
38.25	1.110582	0.647906	0.021298	0.009072	
39	1.086936	0.64253	0.034045	0.021785	
39.75	1.086906	0.641956	0.018979	0.01922	
40.5	1.06173	0.63171	0.021132	0.005853	
41.25	1.048499	0.631308	0.012931	0.017023	
42	1.039538	0.619949	0.015763	0.006187	
42.75	1.016149	0.622102	0.025005	0.011713	
43.5	0.995855	0.61457	0.019361	0.008778	
44.25	1.000266	0.608531	0.034766	0.010542	
45	0.976154	0.598938	0.03472	0.00789	
45.75	0.956942	0.597791	0.027077	0.012899	
46.5	0.953502	0.601504	0.031072	0.011386	
47.25	0.946588	0.591872	0.019219	0.005427	
48	0.921204	0.588991	0.024013	0.011813	
48.75	0.916562	0.591811	0.023251	0.01359	
49.5	0.89814	0.588038	0.022143	0.010039	
50.25	0.888852	0.580514	0.010912	0.014555	
51	0.89171	0.563349	0.030443	0.006083	
51.75	0.863766	0.580647	0.0324	0.000847	
52.5	0.862823	0.568393	0.011329	0.016187	
53.25	0.840258	0.560027	0.023728	0.007655	
54	0.828279	0.571087	0.013985	0.012698	
54.75	0.830313	0.564438	0.018747	0.015336	
55.5	0.815531	0.557563	0.020816	0.013871	
56.25	0.794783	0.552375	0.011822	0.006897	
57	0.799428	0.556824	0.025355	0.007618	
57.75	0.793772	0.547946	0.02594	0.014003	
58.5	0.772503	0.547492	0.015927	0.00991	
59.25	0.76394	0.544581	0.011502	0.018651	
60	0.76782	0.533585	0.017283	0.011034	
60.75	0.75603	0.529146	0.005561	0.006443	
61.5	0.744723	0.521958	0.003803	0.020532	
62.25	0.737824	0.523537	0.006432	0.00713	
63	0.717048	0.521425	0.015455	0.011173	
63.75	0.715394	0.51918	0.012252	0.017649	
64.5	0.70726	0.50344	0.017561	0.008218	
65.25	0.696794	0.517935	0.016552	0.012554	
66	0.686923	0.508984	0.022954	0.010518	
66.75	0.67779	0.507988	0.012751	0.021547	
67.5	0.68041	0.510008	0.008009	0.015646	
68.25	0.673017	0.498012	0.027805	0.00957	
69	0.662904	0.492732	0.027343	0.009725	
69.75	0.642006	0.497764	0.012583	0.007397	

Fig 4C. V2

(n=3)	Mean		S.D.		Statistical method used
Time(min)	SDF1α	Veh.	SDF1α	Veh.	unpaired Student's t-test
0	1.17843	1.204561	0.074371	0.07096	
0.75	1.258576	1.248174	0.053494	0.048965	
1.5	1.274243	1.253687	0.052102	0.066448	
2.25	1.256332	1.245771	0.025751	0.036909	
3	1.251206	1.219622	0.028226	0.059978	
3.75	1.205835	1.193644	0.032686	0.028051	
4.5	1.178672	1.151939	0.047	0.015568	
5.25	1.136845	1.13306	0.00187	0.02168	
6	1.110616	1.107445	0.016526	0.034247	
6.75	1.085202	1.071808	0.001844	0.040947	
7.5	1.05556	1.048878	0.020393	0.036374	
8.25	1.043661	1.041566	0.014549	0.026231	
9	1	1	0	0	
9.75	2.316944	0.815782	0.232684	0.029737	
10.5	3.104209	0.777268	0.142298	0.054775	
11.25	3.38765	0.775475	0.080698	0.059287	
12	3.50712	0.77516	0.120619	0.0629	
12.75	3.444606	0.762261	0.072965	0.053028	

13.5	3.394083	0.759141	0.06006	0.040563	
14.25	3.320684	0.758859	0.042717	0.050671	
15	3.245014	0.746917	0.057463	0.043272	
15.75	3.146141	0.741797	0.045195	0.048189	
16.5	3.078512	0.739441	0.051274	0.038496	
17.25	2.955741	0.733887	0.049407	0.028751	
18	2.889371	0.72379	0.042563	0.035944	
18.75	2.813441	0.71418	0.021688	0.010394	
19.5	2.730295	0.70483	0.015145	0.03518	
20.25	2.700359	0.683782	0.0279	0.020565	
21	2.593268	0.683918	0.040479	0.028621	
21.75	2.576385	0.67644	0.026669	0.025597	
22.5	2.491184	0.677803	0.034319	0.02873	
23.25	2.455116	0.661934	0.028181	0.036401	
24	2.379018	0.655126	0.040996	0.026976	
24.75	2.324427	0.658332	0.028649	0.03064	
25.5	2.294391	0.651848	0.014943	0.033083	
26.25	2.231456	0.635765	0.04974	0.024267	
27	2.178369	0.630936	0.027069	0.026661	
27.75	2.169597	0.635034	0.01015	0.024222	
28.5	2.121264	0.619746	0.024818	0.0287	
29.25	2.090443	0.617358	0.021949	0.019567	
30	2.057371	0.613356	0.015557	0.029221	
30.75	2.025017	0.605404	0.016626	0.018472	
31.5	1.962087	0.598634	0.030334	0.02071	
32.25	1.951833	0.595383	0.033243	0.02035	
33	1.92765	0.594758	0.028913	0.02162	
33.75	1.902693	0.594341	0.023618	0.034422	
34.5	1.871931	0.572916	0.028028	0.036075	
35.25	1.832284	0.575285	0.012356	0.014107	
36	1.808902	0.568619	0.017012	0.017428	
36.75	1.771256	0.558378	0.005765	0.020263	
37.5	1.758767	0.558693	0.027514	0.018335	
38.25	1.726101	0.552364	0.013783	0.028191	
39	1.691264	0.545963	0.007919	0.011476	
39.75	1.6875	0.540085	0.036884	0.013079	
40.5	1.655864	0.546306	0.010442	0.008367	
41.25	1.629192	0.543794	0.023471	0.032054	
42	1.613155	0.543818	0.019396	0.011163	
42.75	1.588421	0.524827	0.028655	0.012684	
43.5	1.583015	0.513741	0.000517	0.021202	
44.25	1.565521	0.514851	0.007557	0.008763	
45	1.537268	0.514124	0.010524	0.03106	
45.75	1.508156	0.499654	0.011297	0.015179	
46.5	1.499278	0.503482	0.011754	0.014831	
47.25	1.471017	0.500884	0.020872	0.00739	
48	1.4604	0.490897	0.01633	0.019452	
48.75	1.441091	0.503181	0.005284	0.003289	
49.5	1.42013	0.493015	0.008952	0.012541	
50.25	1.422913	0.492944	0.018598	0.007576	
51	1.394315	0.476071	0.01359	0.010479	
51.75	1.373244	0.475934	0.013554	0.013055	
52.5	1.356366	0.467603	0.021062	0.012311	
53.25	1.340343	0.4657	0.013338	0.025946	
54	1.327803	0.467392	0.025186	0.012682	
54.75	1.319428	0.471781	0.020452	0.006169	
55.5	1.306135	0.453075	0.021019	0.010975	
56.25	1.285341	0.456934	0.011292	0.009103	
57	1.269568	0.443012	0.014984	0.016699	
57.75	1.242839	0.442812	0.015062	0.017279	
58.5	1.241891	0.450039	0.009818	0.018817	
59.25	1.230129	0.44218	0.017291	0.008615	
60	1.200321	0.434785	0.012666	0.009792	
60.75	1.208416	0.422855	0.005655	0.017189	
61.5	1.192719	0.420729	0.018568	0.016985	

62.25	1.178521	0.422248	0.009795	0.010847	
63	1.153638	0.417438	0.011846	0.006626	
63.75	1.135331	0.410373	0.002452	0.012193	
64.5	1.13406	0.40755	0.015515	0.012075	
65.25	1.130654	0.414144	0.030129	0.016168	
66	1.10505	0.400122	0.010621	0.019618	
66.75	1.101194	0.39958	0.018639	0.009795	
67.5	1.096411	0.394359	0.010774	0.007773	
68.25	1.05937	0.394565	0.015313	0.002982	
69	1.069768	0.392917	0.023462	0.011001	
69.75	1.053293	0.381119	0.01469	0.000668	
**Fig 4C. V3**					
(n=3)	Mean		S.D.		Statistical method used
Time(min)	SDF1α	Veh.	SDF1α	Veh.	unpaired Student's t-test
0	0.849972	0.889028	0.044861	0.023271	
0.75	0.926175	0.958489	0.03361	0.01022	
1.5	0.976238	1.009152	0.030396	0.004597	
2.25	1.010784	1.028139	0.021845	0.025975	
3	1.031391	1.039293	0.029344	0.02435	
3.75	1.03843	1.039483	0.029808	0.013914	
4.5	1.025042	1.042991	0.012293	0.007655	
5.25	1.035219	1.037484	0.024926	0.002031	
6	1.02987	1.037304	0.021948	0.006502	
6.75	1.006186	1.039194	0.016522	0.014677	
7.5	1.008401	1.03973	0.012427	0.000799	
8.25	1.001849	1.020506	0.023213	0.011103	
9	1	1	0	0	
9.75	1.134785	0.799914	0.023365	0.041707	
10.5	1.211245	0.791981	0.018074	0.049396	
11.25	1.195115	0.79595	0.024504	0.044433	
12	1.145603	0.797159	0.011981	0.042369	
12.75	1.091736	0.806268	0.010685	0.023144	
13.5	1.059348	0.804825	0.020164	0.02309	
14.25	1.002817	0.81344	0.004541	0.008728	
15	0.981927	0.809487	0.002572	0.021701	
15.75	0.94106	0.802998	0.003037	0.021476	
16.5	0.936851	0.80217	0.013168	0.01015	
17.25	0.909143	0.794402	0.008688	0.015401	
18	0.886079	0.780206	0.001374	0.002178	
18.75	0.874447	0.778317	0.003549	0.014257	
19.5	0.85017	0.779061	0.008121	0.009076	
20.25	0.840813	0.769481	0.008623	0.00918	
21	0.822871	0.757997	0.008045	0.011148	
21.75	0.806843	0.75576	0.00625	0.005471	
22.5	0.801882	0.743691	0.007895	0.010703	
23.25	0.796338	0.737341	0.008404	0.012658	
24	0.780325	0.727083	0.011608	0.001827	
24.75	0.768439	0.722184	0.006184	0.007382	
25.5	0.754858	0.727993	0.002869	0.002315	
26.25	0.751858	0.711696	0.012799	0.003206	
27	0.737278	0.70692	0.008997	0.013673	
27.75	0.742702	0.707172	0.006598	0.013766	
28.5	0.735362	0.693963	0.005256	0.002446	
29.25	0.718137	0.686301	0.008045	0.003266	
30	0.714864	0.68533	0.015652	0.017402	
30.75	0.704587	0.677317	0.006605	0.002906	
31.5	0.690851	0.672989	0.004824	0.008896	
32.25	0.69818	0.667726	0.010442	0.00527	
33	0.684953	0.657397	0.011574	0.004813	
33.75	0.67623	0.652978	0.005934	0.004576	
34.5	0.660708	0.650765	0.008605	0.004378	
35.25	0.655205	0.645844	0.005676	0.010295	
36	0.641587	0.640098	0.004842	0.012872	
36.75	0.647789	0.63145	0.012663	0.000248	
37.5	0.64724	0.632899	0.005733	0.006724	

38.25	0.624791	0.626412	0.011012	0.007861	
39	0.624677	0.612311	0.003909	0.002836	
39.75	0.613175	0.607656	0.009831	0.003086	
40.5	0.620657	0.60982	0.005351	0.009857	
41.25	0.6169	0.606716	0.017336	0.002096	
42	0.604298	0.598981	0.012307	0.008798	
42.75	0.600503	0.597046	0.008337	0.003866	
43.5	0.599102	0.591223	0.012558	0.00183	
44.25	0.586377	0.58627	0.00944	0.006806	
45	0.582436	0.587015	0.019407	0.013219	
45.75	0.584747	0.577735	0.007912	0.004391	
46.5	0.57623	0.582417	0.005926	0.013358	
47.25	0.572249	0.570474	0.014542	0.009726	
48	0.574831	0.562488	0.006342	0.0111	
48.75	0.568441	0.561015	0.006081	0.003478	
49.5	0.554861	0.556595	0.011803	0.003336	
50.25	0.555223	0.554405	0.006079	0.009576	
51	0.553239	0.55007	0.009737	0.006843	
51.75	0.551657	0.544814	0.006302	0.009603	
52.5	0.543836	0.542011	0.016007	0.008512	
53.25	0.542325	0.546469	0.004337	0.003898	
54	0.538627	0.536097	0.007703	0.008641	
54.75	0.528814	0.535835	0.004338	0.003349	
55.5	0.529381	0.528599	0.010555	0.011445	
56.25	0.523763	0.529547	0.020974	0.00971	
57	0.513772	0.523276	0.011891	0.012581	
57.75	0.515124	0.514855	0.004429	0.004215	
58.5	0.501891	0.516683	0.011744	0.00999	
59.25	0.507773	0.508588	0.00949	0.010591	
60	0.495586	0.511193	0.001835	0.00797	
60.75	0.49638	0.507029	0.018144	0.000563	
61.5	0.491275	0.50916	0.011732	0.008974	
62.25	0.490645	0.496868	0.011863	0.004257	
63	0.483989	0.503238	0.008974	0.007672	
63.75	0.480304	0.487968	0.011655	0.014239	
64.5	0.479403	0.481515	0.005972	0.006554	
65.25	0.46964	0.481605	0.015724	0.006955	
66	0.465456	0.485451	0.011314	0.004785	
66.75	0.468353	0.481717	0.024505	0.010175	
67.5	0.462753	0.474341	0.0057	0.01055	
68.25	0.459026	0.469934	0.005531	0.008867	
69	0.451866	0.464421	0.008636	0.006291	
69.75	0.449136	0.459506	0.013435	0.005795	
**Fig 4C. V4**					
(n=3)	Mean		S.D.		Statistical method used
Time(min)	SDF1α	Veh.	SDF1α	Veh.	unpaired Student's t-test
0	0.881275	0.92041	0.04013	0.026117	
0.75	0.954998	0.972476	0.018195	0.01255	
1.5	0.987584	1.015738	0.021147	0.008988	
2.25	1.019816	1.032577	0.020708	0.010436	
3	1.021771	1.043287	0.006426	0.019893	
3.75	1.028083	1.037393	0.011139	0.014475	
4.5	1.032394	1.04738	0.011792	0.001252	
5.25	1.028086	1.03859	0.014779	0.013598	
6	1.029272	1.044711	0.008927	0.01429	
6.75	1.022215	1.028613	0.00855	0.019013	
7.5	1.012613	1.030828	0.024076	0.014612	
8.25	1.00209	1.012808	0.010396	0.010531	
9	1	1	0	0	
9.75	1.168446	0.835778	0.046296	0.036711	
10.5	1.177748	0.822455	0.014064	0.029115	
11.25	1.141834	0.823137	0.004222	0.032658	
12	1.097415	0.819651	0.003013	0.022906	
12.75	1.032978	0.819453	0.005527	0.015858	
13.5	1.00832	0.81909	0.001522	0.014928	

14.25	0.983445	0.818266	0.011491	0.013671	
15	0.941491	0.813509	0.003846	0.010915	
15.75	0.930159	0.798513	0.010871	0.006211	
16.5	0.902156	0.797509	0.008768	0.018368	
17.25	0.896409	0.793082	0.005134	0.010098	
18	0.8702	0.779026	0.023515	0.016849	
18.75	0.853639	0.774788	0.013791	0.007698	
19.5	0.84093	0.765609	0.013772	0.016082	
20.25	0.827871	0.757792	0.013443	0.012676	
21	0.807113	0.749855	0.018704	0.013106	
21.75	0.795457	0.738617	0.010404	0.020117	
22.5	0.793015	0.727215	0.00564	0.017199	
23.25	0.780087	0.729623	0.013029	0.016467	
24	0.765853	0.720349	0.005032	0.018381	
24.75	0.749801	0.71902	0.00731	0.009638	
25.5	0.749476	0.709042	0.017118	0.013966	
26.25	0.74207	0.705409	0.008143	0.002871	
27	0.732964	0.694966	0.015346	0.008767	
27.75	0.728893	0.693688	0.004945	0.003697	
28.5	0.710982	0.681805	0.00854	0.010905	
29.25	0.717529	0.680189	0.003383	0.003666	
30	0.705719	0.679419	0.013674	0.015071	
30.75	0.699412	0.663609	0.007922	0.003695	
31.5	0.683286	0.658658	0.007212	0.007291	
32.25	0.677235	0.65805	0.004965	0.016642	
33	0.672892	0.651922	0.004741	0.012553	
33.75	0.662732	0.646604	0.0044	0.006849	
34.5	0.67175	0.639386	0.008226	0.01637	
35.25	0.660604	0.627131	0.009205	0.013265	
36	0.659633	0.627333	0.008486	0.006512	
36.75	0.639152	0.616305	0.00497	0.014869	
37.5	0.632996	0.623041	0.009977	0.007853	
38.25	0.630789	0.616997	0.004518	0.005769	
39	0.627217	0.609643	0.00634	0.003071	
39.75	0.619409	0.60926	0.006299	0.015092	
40.5	0.612425	0.601612	0.010662	0.007732	
41.25	0.60782	0.594491	0.010044	0.009327	
42	0.592842	0.590229	0.009281	0.016357	
42.75	0.595553	0.586329	0.006071	0.012792	
43.5	0.596676	0.574619	0.00824	0.014729	
44.25	0.582093	0.574003	0.006674	0.013591	
45	0.577921	0.577855	0.008225	0.021022	
45.75	0.57828	0.570804	0.007651	0.01349	
46.5	0.5732	0.56933	0.007306	0.005922	
47.25	0.56876	0.550243	0.010424	0.020552	
48	0.553336	0.557548	0.00771	0.020764	
48.75	0.554799	0.551139	0.011198	0.011504	
49.5	0.554698	0.543812	0.008077	0.016209	
50.25	0.551247	0.545978	0.007008	0.008901	
51	0.5459	0.539004	0.002414	0.00532	
51.75	0.542347	0.536843	0.018616	0.016542	
52.5	0.545509	0.534872	0.014814	0.010913	
53.25	0.541641	0.532204	0.007601	0.013437	
54	0.530656	0.532011	0.007866	0.007062	
54.75	0.522557	0.526884	0.00147	0.012436	
55.5	0.523302	0.521284	0.004855	0.009868	
56.25	0.517001	0.511931	0.012593	0.008128	
57	0.521507	0.511808	0.003025	0.009531	
57.75	0.514868	0.515337	0.007257	0.009087	
58.5	0.494806	0.502745	0.002522	0.015488	
59.25	0.499719	0.496884	0.007726	0.005977	
60	0.495579	0.495604	0.011755	0.003413	
60.75	0.500107	0.490794	0.004334	0.013965	
61.5	0.499262	0.483791	0.004606	0.014481	
62.25	0.490667	0.490913	0.008604	0.012463	

63	0.491395	0.491796	0.023448	0.011059	
63.75	0.476609	0.481345	0.016465	0.011683	
64.5	0.477631	0.475466	0.009827	0.007983	
65.25	0.469783	0.469301	0.008775	0.012083	
66	0.468351	0.466641	0.008092	0.011673	
66.75	0.466084	0.470854	0.017612	0.01144	
67.5	0.459989	0.461647	0.00987	0.011913	
68.25	0.456465	0.458142	0.009438	0.013598	
69	0.449806	0.45585	0.010622	0.011761	
69.75	0.451179	0.455584	0.006849	0.009402	
**Fig 4C. V5**					
(n=3)	Mean		S.D.		Statistical method used
Time(min)	SDF1α	Veh.	SDF1α	Veh.	unpaired Student's t-test
0	0.902741	0.90434	0.041375	0.027232	
0.75	0.972017	0.985372	0.042307	0.019799	
1.5	1.033314	1.031875	0.018294	0.011445	
2.25	1.035831	1.041386	0.010655	0.020204	
3	1.043445	1.056704	0.014611	0.012332	
3.75	1.050679	1.05062	0.015546	0.0056	
4.5	1.058155	1.044674	0.004659	0.006735	
5.25	1.037416	1.046222	0.017348	0.008678	
6	1.035434	1.042504	0.004165	0.009173	
6.75	1.031617	1.026956	0.023002	0.002806	
7.5	1.006662	1.025164	0.008576	0.000576	
8.25	1.007885	1.019183	0.011991	0.010866	
9	1	1	0	0	
9.75	1.96488	0.846951	0.10935	0.013025	
10.5	2.382751	0.828127	0.008927	0.025158	
11.25	2.3967	0.820946	0.01461	0.02359	
12	2.296678	0.822797	0.04063	0.012287	
12.75	2.181036	0.814965	0.066122	0.012594	
13.5	2.037472	0.82309	0.065388	0.015028	
14.25	1.93466	0.81503	0.064598	0.005248	
15	1.831791	0.816257	0.050858	0.00672	
15.75	1.763204	0.791017	0.056387	0.01368	
16.5	1.680756	0.788482	0.034149	0.009236	
17.25	1.611325	0.777911	0.020089	0.007216	
18	1.539812	0.775522	0.041892	0.00539	
18.75	1.498491	0.760781	0.029236	0.008656	
19.5	1.450058	0.763978	0.013145	0.009097	
20.25	1.393832	0.743935	0.020176	0.011463	
21	1.354337	0.747361	0.011688	0.00505	
21.75	1.314128	0.739819	0.012507	0.003015	
22.5	1.282086	0.7285	0.012979	0.002985	
23.25	1.241616	0.721051	0.032402	0.006012	
24	1.214249	0.699647	0.012548	0.012141	
24.75	1.189875	0.713218	0.017706	0.009988	
25.5	1.164073	0.699296	0.029559	0.003316	
26.25	1.147755	0.691988	0.012552	0.014617	
27	1.109084	0.695351	0.01304	0.004961	
27.75	1.09498	0.680245	0.018246	0.008185	
28.5	1.061633	0.672141	0.01601	0.003565	
29.25	1.042759	0.666209	0.020422	0.007017	
30	1.014632	0.664695	0.025138	0.009912	
30.75	1.00416	0.651679	0.015179	0.00743	
31.5	0.974902	0.65499	0.020575	0.017494	
32.25	0.969962	0.654094	0.017227	0.010195	
33	0.940163	0.640032	0.021521	0.010947	
33.75	0.932954	0.64027	0.01545	0.006591	
34.5	0.919054	0.633495	0.024729	0.007253	
35.25	0.90527	0.624912	0.026803	0.005705	
36	0.879162	0.619015	0.017466	0.010338	
36.75	0.873057	0.605069	0.004252	0.00955	
37.5	0.853541	0.604635	0.010517	0.001806	
38.25	0.832925	0.602055	0.020375	0.002061	

39	0.832183	0.60294	0.013235	0.0033	
39.75	0.810468	0.595706	0.021069	0.001105	
40.5	0.803078	0.594917	0.024259	0.002936	
41.25	0.783762	0.589138	0.017103	0.004217	
42	0.78303	0.581676	0.024954	0.011322	
42.75	0.762448	0.577698	0.016496	0.002739	
43.5	0.754268	0.574269	0.019323	0.007765	
44.25	0.750063	0.564888	0.019703	0.004956	
45	0.741419	0.570027	0.009637	0.011682	
45.75	0.720512	0.563713	0.021798	0.007941	
46.5	0.713865	0.562432	0.009317	0.019977	
47.25	0.706758	0.555166	0.02287	0.00875	
48	0.701003	0.553742	0.020366	0.005645	
48.75	0.689317	0.545238	0.021321	0.003777	
49.5	0.67902	0.54236	0.022328	0.005456	
50.25	0.668781	0.541911	0.021195	0.009437	
51	0.654793	0.530844	0.015612	0.001869	
51.75	0.64834	0.529804	0.019014	0.005752	
52.5	0.645166	0.537181	0.007303	0.010344	
53.25	0.629917	0.522373	0.017794	0.007841	
54	0.627438	0.521662	0.013426	0.008801	
54.75	0.614114	0.519191	0.009004	0.006335	
55.5	0.615859	0.516015	0.010365	0.006325	
56.25	0.605928	0.510092	0.013324	0.001312	
57	0.597548	0.508802	0.018742	0.013889	
57.75	0.592117	0.50375	0.01181	0.005031	
58.5	0.577653	0.489586	0.019698	0.004232	
59.25	0.579023	0.489425	0.012549	0.005348	
60	0.566514	0.492938	0.012934	0.002374	
60.75	0.561047	0.487952	0.009309	0.00377	
61.5	0.549588	0.479054	0.011629	0.006462	
62.25	0.54573	0.473626	0.017067	0.00297	
63	0.537636	0.479975	0.016636	0.006192	
63.75	0.538084	0.476185	0.017446	0.004014	
64.5	0.521494	0.473615	0.010052	0.00434	
65.25	0.524434	0.467202	0.006111	0.007274	
66	0.511744	0.459847	0.007061	0.007038	
66.75	0.508845	0.46113	0.015655	0.003055	
67.5	0.502923	0.46148	0.014336	0.005812	
68.25	0.490712	0.451991	0.01805	0.001789	
69	0.491178	0.451875	0.005297	0.005564	
69.75	0.486678	0.440504	0.01022	0.009475	

**Fig 4D. V1**					
(n=3)	Mean		S.D.		Statistical method used
Time(min)	SDF1α	Veh.	SDF1α	Veh.	unpaired Student's t-test
0	1.338218	1.30845	0.55348	0.208798	
1.5	1.086244	1.24799	0.146603	0.50451	
3	1.04232	1.105514	0.158822	0.235163	
4.5	0.947005	1.154262	0.194015	0.285652	
6	1.098139	1.079393	0.110789	0.113674	
7.5	1.088434	1.170832	0.029486	0.155262	
9	1	1	0	0	
11	0.821855	0.876632	0.196822	0.110323	
12.5	1.172292	0.89597	0.078028	0.106406	
14	1.425804	0.81245	0.416996	0.102159	
15.5	1.547759	0.84043	0.108923	0.105655	
17	1.823062	0.772329	0.339599	0.143311	
18.5	1.783968	1.018747	0.505217	0.115863	
20	1.755217	0.962411	0.403477	0.067227	
21.5	1.962619	0.764927	0.592586	0.124802	
23	1.828842	1.036188	0.476922	0.14606	
24.5	1.863567	1.056967	0.557229	0.485916	
26	1.687744	0.901659	0.632251	0.078272	
27.5	1.642388	0.847374	0.329401	0.172599	

29	1.525926	0.793984	0.220004	0.189362	
30.5	1.55652	0.887986	0.349009	0.292543	
32	1.402302	0.711155	0.289553	0.137892	
33.5	1.360396	0.787136	0.113122	0.097784	
35	1.396614	0.793173	0.489842	0.15779	
36.5	1.334203	0.908202	0.152086	0.171577	
38	1.396632	0.771193	0.235331	0.306179	
39.5	1.327357	1.051166	0.264454	0.271698	
41	1.287287	0.977781	0.412238	0.21633	
42.5	1.307609	0.839093	0.434976	0.227971	
44	1.27343	0.723828	0.53352	0.155421	
45.5	1.066328	0.820781	0.252579	0.127904	
47	1.289966	0.709875	0.236142	0.197699	
48.5	1.347902	0.887165	0.335348	0.346389	
50	1.21899	0.781856	0.13206	0.126384	
51.5	1.365624	0.592864	0.225315	0.07903	
53	1.183356	0.785537	0.077621	0.294675	
54.5	1.325438	0.884138	0.393752	0.212656	
56	1.333906	0.833455	0.344392	0.309942	
57.5	1.223672	0.817356	0.311034	0.284476	
59	1.285379	0.812085	0.245252	0.129558	
60.5	1.240062	0.94608	0.230031	0.353862	
62	1.352364	0.583966	0.43662	0.047818	
63.5	1.027281	0.699713	0.275488	0.107251	
65	1.14414	0.841563	0.290987	0.165998	
66.5	1.064017	0.697842	0.402233	0.073432	
68	1.258946	0.80524	0.351204	0.112375	
69.5	1.23062	0.706756	0.237128	0.091618	
71	1.072267	0.677221	0.207443	0.10142	
**Fig 4D. V2**					
(n=3)	Mean		S.D.		Statistical method used
Time(min)	SDF1α	Veh.	SDF1α	Veh.	unpaired Student's t-test
0	0.988756	0.978389	0.108921	0.14644	
1.5	1.15064	1.060752	0.110882	0.089178	
3	1.20581	1.114528	0.144545	0.385506	
4.5	1.06505	1.041282	0.03147	0.283162	
6	1.065437	1.009617	0.098961	0.221567	
7.5	1.030705	1.059353	0.125597	0.166181	
9	1	1	0	0	
11	1.075251	0.93375	0.079276	0.19597	
12.5	1.870414	1.02175	0.167223	0.215426	
14	3.30471	0.866958	0.376447	0.072837	
15.5	4.698783	1.077896	0.160449	0.227603	
17	5.602711	0.771241	0.375615	0.280003	
18.5	5.891111	1.049028	0.283642	0.30022	
20	6.30816	0.878741	0.289027	0.112183	
21.5	6.460025	0.823153	0.694487	0.216545	
23	6.774382	0.80058	0.299215	0.030189	
24.5	6.610788	0.934389	0.377232	0.29421	
26	6.916362	0.795499	1.026614	0.257455	
27.5	6.89731	0.805673	0.316504	0.063977	
29	6.558636	0.892677	0.40447	0.113512	
30.5	6.197142	0.870927	0.313702	0.1112	
32	6.268444	0.894866	0.238776	0.233267	
33.5	6.325137	0.780245	0.488146	0.117647	
35	5.763139	0.757207	0.172779	0.092073	
36.5	5.746418	0.875266	0.374446	0.101257	
38	5.63287	0.778847	0.509307	0.164724	
39.5	5.4408	0.701524	0.06022	0.197556	
41	5.447988	0.874489	0.463227	0.122147	
42.5	5.321499	0.750332	0.184386	0.095059	
44	5.481748	0.708522	0.479306	0.17038	
45.5	5.004846	0.782378	0.450883	0.215508	
47	4.926716	0.651951	0.108738	0.2993	
48.5	4.871176	0.829943	0.610414	0.133794	

50	4.967815	0.759228	0.340327	0.191571	
51.5	4.634462	0.886604	0.262827	0.082012	
53	4.529377	0.779484	0.50771	0.18443	
54.5	4.559907	0.677459	0.171347	0.229558	
56	4.358372	0.838624	0.326383	0.16823	
57.5	4.172597	0.761829	0.248713	0.097934	
59	4.372251	0.767353	0.133112	0.200629	
60.5	4.048551	0.623748	0.209745	0.187762	
62	4.001984	0.700549	0.137479	0.238973	
63.5	3.832783	0.683207	0.228796	0.144766	
65	3.911826	0.763249	0.099738	0.029616	
66.5	3.658909	0.702763	0.350558	0.121849	
68	3.766121	0.674991	0.053594	0.190623	
69.5	3.859384	0.654176	0.526717	0.132783	
71	3.527831	0.66746	0.277354	0.112709	
**Fig 4D. V3**					
(n=3)	Mean		S.D.		Statistical method used
Time(min)	SDF1α	Veh.	SDF1α	Veh.	unpaired Student's t-test
0	0.977633	1.176376	0.146411	0.50833	
1.5	1.012199	1.191333	0.28594	0.233913	
3	0.939575	1.29651	0.115879	0.393939	
4.5	1.088198	1.328088	0.24189	0.570302	
6	0.998082	1.213689	0.365085	0.499112	
7.5	1.138947	1.024283	0.170435	0.313695	
9	1	1	0	0	
11	0.85064	0.992266	0.182636	0.267529	
12.5	0.997957	1.002788	0.147144	0.492441	
14	1.04891	0.935071	0.205245	0.312148	
15.5	1.207042	1.031954	0.474299	0.321234	
17	1.13584	0.988433	0.234086	0.468473	
18.5	1.321598	1.065775	0.395773	0.382511	
20	1.445121	0.891231	0.116029	0.195924	
21.5	1.366262	0.904138	0.087247	0.221731	
23	1.116265	1.024185	0.213243	0.344337	
24.5	1.252285	0.84086	0.113804	0.164237	
26	1.131566	1.08213	0.257798	0.544427	
27.5	1.126484	1.088197	0.327246	0.321331	
29	1.195187	0.864512	0.428309	0.312135	
30.5	0.990715	0.887302	0.307271	0.428821	
32	0.809903	0.78586	0.231283	0.290464	
33.5	1.00975	1.128766	0.08938	0.584412	
35	0.914035	0.820004	0.116403	0.226535	
36.5	0.92577	0.746622	0.192188	0.080245	
38	1.043876	0.89598	0.248437	0.292276	
39.5	0.992409	0.928498	0.069404	0.128984	
41	0.873993	1.100401	0.258635	0.21966	
42.5	0.759545	1.031998	0.15691	0.378748	
44	0.955079	0.761459	0.274159	0.37233	
45.5	0.81629	1.004172	0.149075	0.447104	
47	0.809306	0.801881	0.099746	0.273603	
48.5	1.151654	0.854494	0.172554	0.34495	
50	0.844473	0.870929	0.103392	0.212542	
51.5	0.82474	0.817409	0.269817	0.203109	
53	0.97225	0.760812	0.112471	0.270694	
54.5	0.931298	0.777188	0.078112	0.361881	
56	0.911493	0.851885	0.196481	0.160706	
57.5	0.930665	0.810017	0.17999	0.342617	
59	0.973568	0.855082	0.157915	0.049823	
60.5	0.799631	0.895873	0.072246	0.165404	
62	0.831312	0.766855	0.069709	0.041204	
63.5	0.766198	0.972932	0.07117	0.066263	
65	0.660005	0.955326	0.08755	0.368667	
66.5	0.896582	0.688389	0.110679	0.313774	
68	0.990276	0.780803	0.207492	0.031055	
69.5	0.74984	0.582697	0.166725	0.180557	

71	0.761641	0.698695	0.046015	0.162461	
**Fig 4D. V4**					
(n=3)	Mean		S.D.		Statistical method used
Time(min)	SDF1α	Veh.	SDF1α	Veh.	unpaired Student's t-test
0	1.097674	1.386785	0.150541	0.296307	
1.5	1.145139	1.123947	0.019862	0.220994	
3	1.158705	1.128826	0.154306	0.135525	
4.5	1.01134	1.323131	0.033581	0.221913	
6	1.18682	1.350727	0.094103	0.182185	
7.5	1.048125	1.180746	0.189054	0.211265	
9	1	1	0	0	
11	0.787469	0.874747	0.194436	0.080983	
12.5	0.876722	0.983377	0.080709	0.302895	
14	1.007319	0.982525	0.085377	0.266344	
15.5	1.086342	0.882467	0.070555	0.238215	
17	0.777203	0.925801	0.063525	0.152915	
18.5	1.028473	0.921222	0.139606	0.25607	
20	1.32465	1.028049	0.252403	0.200714	
21.5	1.217463	1.059065	0.24527	0.181792	
23	0.989783	0.939145	0.110898	0.276106	
24.5	0.819692	0.770793	0.021551	0.201364	
26	0.858215	1.016975	0.129536	0.295199	
27.5	0.879144	1.021228	0.082694	0.074386	
29	0.952623	0.781087	0.308421	0.185671	
30.5	0.974171	0.948265	0.172278	0.24858	
32	0.792671	0.713631	0.062036	0.116218	
33.5	0.79518	0.920724	0.0363	0.225388	
35	0.908499	0.78568	0.157014	0.181096	
36.5	0.98474	0.989631	0.397339	0.068851	
38	0.977139	0.875175	0.21433	0.15672	
39.5	0.837068	0.727442	0.09544	0.143273	
41	0.786643	0.975384	0.398683	0.318001	
42.5	0.709399	0.826925	0.045309	0.183759	
44	0.943032	0.754144	0.058493	0.432537	
45.5	0.685354	0.794782	0.150045	0.085455	
47	0.826918	0.752249	0.130386	0.098136	
48.5	0.86833	0.713985	0.227886	0.135501	
50	0.867995	0.851374	0.104226	0.176319	
51.5	0.919536	0.813213	0.1918	0.152416	
53	0.949102	0.684165	0.228334	0.229863	
54.5	0.913322	0.821701	0.145218	0.209186	
56	0.694985	0.658596	0.132381	0.337792	
57.5	0.968118	0.770788	0.298677	0.115711	
59	0.7101	0.769723	0.119786	0.087616	
60.5	0.882322	0.819449	0.220877	0.125192	
62	0.810422	0.763747	0.067583	0.16931	
63.5	0.75163	0.719156	0.099258	0.252369	
65	0.76056	0.895074	0.018522	0.221239	
66.5	0.524372	0.685888	0.031345	0.113734	
68	0.773512	0.665739	0.372802	0.128038	
69.5	0.73281	0.741843	0.089895	0.132637	
71	0.938096	0.900187	0.448305	0.215431	
**Fig 4D. V5**					
(n=3)	Mean		S.D.		Statistical method used
Time(min)	SDF1α	Veh.	SDF1α	Veh.	unpaired Student's t-test
0	1.208913	1.278053	0.115304	0.033093	
1.5	1.168183	1.337998	0.188507	0.267113	
3	1.111794	1.27441	0.235766	0.129915	
4.5	1.193278	1.391754	0.256602	0.11277	
6	1.132422	1.334304	0.242672	0.186535	
7.5	1.098184	1.142177	0.171001	0.141027	
9	1	1	0	0	
11	0.992815	1.043956	0.167317	0.136199	
12.5	0.862037	0.965611	0.169309	0.217995	
14	1.224964	1.102216	0.180749	0.111859	

15.5	1.003632	1.109504	0.070972	0.325095	
17	1.183361	1.043112	0.180352	0.128889	
18.5	1.009672	0.925409	0.167564	0.056417	
20	1.395978	0.909295	0.209722	0.093818	
21.5	1.278929	0.972426	0.17617	0.076415	
23	1.246013	0.991039	0.057114	0.054159	
24.5	1.152687	0.974385	0.222451	0.372512	
26	1.267379	0.864236	0.255234	0.057439	
27.5	1.378939	0.898608	0.449184	0.10589	
29	1.160541	0.809187	0.201726	0.14955	
30.5	1.324036	0.789823	0.05215	0.029901	
32	1.252745	0.851465	0.063686	0.302099	
33.5	1.318823	0.886356	0.465084	0.055241	
35	1.214966	0.856952	0.065611	0.145709	
36.5	1.147459	0.882001	0.112948	0.082139	
38	1.07948	0.90283	0.171311	0.288622	
39.5	1.015734	0.842677	0.224793	0.139699	
41	0.953088	1.001228	0.271296	0.186623	
42.5	1.147348	0.920735	0.16296	0.330843	
44	1.100617	0.852447	0.154898	0.140897	
45.5	1.169821	0.874638	0.138155	0.237307	
47	0.926538	0.82344	0.150221	0.123447	
48.5	0.866755	0.744611	0.133709	0.230798	
50	0.917806	0.77087	0.197665	0.152787	
51.5	0.929914	0.68195	0.060514	0.055905	
53	0.950547	0.837172	0.08725	0.031087	
54.5	1.089737	0.917966	0.039781	0.11522	
56	1.042579	0.895241	0.221639	0.147614	
57.5	0.996622	0.763693	0.136312	0.135854	
59	0.978924	0.682819	0.141741	0.115938	
60.5	0.934361	0.834923	0.156847	0.455991	
62	1.005024	0.643313	0.097629	0.048498	
63.5	1.040176	0.847925	0.132278	0.139298	
65	0.883473	0.755843	0.086546	0.173571	
66.5	0.832017	0.730964	0.064674	0.12708	
68	0.938388	0.783273	0.033667	0.125999	
69.5	0.993878	0.793622	0.154991	0.305634	
71	0.808063	0.858004	0.072772	0.189453	

(n=3)	Mean		S.D.		Statistical method used	P value
**Fig 5C. HiBiT**	SDF1α	Veh.	SDF1α	Veh.	unpaired Student's t-test	**p<0.01 *p<0.05
Vector	379.6667	162	75.00222	114.2935		
V1	32048	49230.33	2981.061	2355.726		
V2	71414	123101.7	1298.08	5697.396		
V3	490.3333	599.3333	230.6715	179.5838		
V4	2203	2095.333	746.1159	770.1697		
V5	27202.33	22792	4398.237	201.6358		

**Fig 5D. V1**					
(n=3)	Mean		S.D.		Statistical method used
Time(min)	SDF1α	Veh.	SDF1α	Veh.	unpaired Student's t-test
0	110821.3	97899	3614.794	6649.192	
0.75	109956.3	100794.7	6154.817	3204.05	
1.5	121216.3	109374	5849.769	12532.13	
2.25	119826.7	110083.3	8098.683	9660.399	
3	116966.7	114596	2128.072	4275.29	
3.75	118416.3	106438	4177.225	6224.262	
4.5	118592.3	109323	5003.671	7803.895	
5.25	113876.7	109180	3909.694	8365.965	
6	109498.7	111029.7	4500.384	7113.098	
6.75	116552.7	103040	8591.746	10989.65	
7.5	114201	109789.7	7428.883	9022.776	

8.25	112548.7	106939	4095.968	9507.626	
9	110799.3	109087.7	10641.46	13152.29	
9.75	102033	106913.3	1436.098	11223.27	
11.13	97325.67	89222	11166.66	11197.1	
11.88	92804.67	87862.67	7966.205	8238.377	
12.63	92009.67	87923.67	6921.01	3213.577	
13.38	90784.67	84288	9619.089	5112.856	
14.13	89614.33	89363.67	5451.853	5138.707	
14.88	88769.33	87513.67	5377.532	7037.282	
15.63	86170.33	85815.33	2712.957	7844.684	
16.38	86723	83851	4433.422	6787.383	
17.13	86466	87857.67	5414.135	9096.462	
17.88	86607.33	86441.33	5545.21	5920.745	
18.63	83986.67	84296.33	3386.548	5930.216	
19.38	81315.33	83145.67	4014.039	5166.077	
20.13	85332.67	85526.67	3516.274	7083.237	
20.88	78375.67	83782	3541.928	5354.43	
21.63	82285.33	84993.67	2986.567	5661.241	
22.38	81903.67	83666	2417.493	7063.444	
23.13	79594.67	82917.33	3048.923	4217.543	
23.88	80423.33	80614.67	6108.683	4069.933	
24.63	80303.67	83001	2533.067	5916.402	
25.38	77477.33	81697.33	4943.816	5022.776	
26.13	77695	79435	7853.698	7704.947	
26.88	76779.67	80290	4376.879	5576.485	
27.63	76855	80217	2984.422	7238.337	
28.38	75555.67	78521	6088.389	7713.877	
29.13	70509	79459.33	3603.524	5966.108	
29.88	75997	79207.67	4514.796	4589.972	
30.63	74564.67	73689.33	415.8573	7505.521	
31.38	72351.33	78007.33	2882.921	9727.404	
32.13	72261.33	76115.33	4766.491	4240.921	
32.88	70146.33	73170.67	6047.697	4432.548	
33.63	71512	74449	4515.295	6136.03	
34.38	70289	74539.67	1476.543	5141.079	
35.13	70913	72768	2635.251	5511.364	
35.88	69367	71096	3416.036	6945.885	
36.63	72440.33	74492.33	5316.504	5082.189	
37.38	70128	73704	3320.419	7565.202	
38.13	68846.33	71325.67	1799.838	5578.169	
38.88	67920.67	70744	3452.877	3732.684	
39.63	66817.33	70735.33	5523.127	5785.883	
40.38	67223.67	69826.67	2262.522	6980.742	
41.13	64986.67	69490	3704.739	6374.561	
41.88	63937.67	70360.33	3272.455	7135.911	
42.63	63328	68458.67	1747.418	8454.348	
43.38	65837	69556.33	3486.493	7307.437	
44.13	64398.33	67046.33	2144.206	7820.666	
44.88	61054	66661.67	5046.736	3504.412	
45.63	61516.67	67035	3954.559	4897.847	
46.38	61684.67	68754.33	2132.897	5084.366	
47.13	61676	65109.67	2744.386	4131.755	
47.88	59874	66778.67	1396.262	6106.332	
48.63	62182	65250.67	2473.076	7434.666	
49.38	60658.33	63799	2435.891	4117.065	
50.13	57557	64131.67	2217.807	7038.522	
50.88	57871	63593.67	1819.44	5043.611	
51.63	58510.67	63907.33	3899.205	4183.059	
52.38	57829.67	64160	2761.948	5447.489	
53.13	57826	63966	2536.225	4799.493	
53.88	57352.33	65081	4136.91	6314.285	
54.63	57604.33	61385	3289.113	7413.204	
55.38	56371.67	61606.33	2725.627	5985.549	
56.13	55151.67	62942	1299.294	3939.156	
56.88	55736.33	62000	3102.015	3865.41	

57.63	55438.67	60366.33	3508.573	5751.135	
58.38	55635	61112	2457.477	5993.302	
59.13	52818.33	59974.67	3604.23	4596.847	
59.88	52837.33	60467	4324.559	4137	
60.63	53160.67	61434.33	3260.444	4841.556	
61.38	51796.33	60023.33	4677.904	5954.371	
62.13	51843.33	56968.67	3554.09	5508.561	
62.88	50198.67	58773	2230.536	3567.087	
63.63	52078	58208.67	3390.301	5599.211	
64.38	51563.67	56979.33	1880.974	5315.436	
65.13	50013	56613	1164.864	7721.992	
65.88	49465	56747.33	2467.405	6754.387	
66.63	50642.33	56085.33	3087.616	3624.563	
67.38	48647	56097	2396.113	5457.098	
68.13	49977.33	55240	4107.463	2771.307	
68.88	49073	55325.67	2409.703	5205.168	
69.63	49066.33	56293	2541.048	7007.981	
70.38	47504.33	55870	3545.13	4064.515	
71.13	49739.33	53869	617.1907	3495.045	
**Fig 5D. V2**					
(n=3)	Mean		S.D.		Statistical method used
Time(min)	SDF1α	Veh.	SDF1α	Veh.	unpaired Student's t-test
0	137397.3	120385.7	8362.552	3720.534	
0.75	146127.3	124078.3	8025.445	9840.891	
1.5	151724.3	140306.3	8349.794	16500.06	
2.25	149193.7	140959.7	2929.359	9900.762	
3	160415.7	136759.3	13153.93	6549.468	
3.75	143773.3	146026.3	16015.87	9626.755	
4.5	144279	142339.3	3596.364	316.8428	
5.25	156945	149408.7	5324.069	13737.52	
6	139117	139695.3	6649.351	10338.02	
6.75	148665	143061.3	7195.776	6123.312	
7.5	147426.7	134744.7	10437.81	14962.2	
8.25	148465	131550.3	7638.92	7553.539	
9	144355.3	144162	4154.803	12212.68	
9.75	143125	142874.3	13765.65	5903.81	
11.13	119003.7	118478.7	2651.082	1143.724	
11.88	118297.7	113880.3	4219.046	9936.057	
12.63	116204.7	120911.3	10885.64	11898.61	
13.38	112144.3	118856.7	7412.071	11329.37	
14.13	106625.7	120540.7	3043.242	6767.189	
14.88	111204	118670.3	10345.22	7464.317	
15.63	99731.67	119096.7	5044.238	11820.88	
16.38	103138.3	124958.3	7584.699	13984.27	
17.13	105632.3	123740.3	2622.453	12713.27	
17.88	102828	117579.7	6908.855	8325.357	
18.63	102683	122244.7	5077.706	6622.506	
19.38	101743.3	117716	4182.003	14168.43	
20.13	96771.33	119792.3	2620.058	9732.068	
20.88	93814	119167.7	2694.109	11124.86	
21.63	94414	110932.3	2728.511	9630.347	
22.38	95004	117486	508.425	7303.862	
23.13	93113.67	112120.7	3186.933	6563.977	
23.88	94389.33	112279.3	12876.19	13460.86	
24.63	92622.33	119825.7	606.585	10292.52	
25.38	91821	115397.3	7238.072	5320.061	
26.13	95593.67	107519.3	6883.511	9655.576	
26.88	95192.67	111485.3	2876.165	2115.642	
27.63	90083.67	116781	3314.073	10020.09	
28.38	86278.33	109339	3016.199	6692.425	
29.13	87957	110773	6521.868	1999.376	
29.88	93073	116433.3	2728.947	1797.523	
30.63	86759.33	114309.3	5175.21	12916.25	
31.38	84915	106587.7	2086.351	5420.651	
32.13	83597.33	108674.7	7374.278	5313.189	

32.88	85863.33	109797.7	3599.687	6351.905	
33.63	84642	105154.7	1517.571	5453.496	
34.38	84161.33	103730	892.4037	6192.72	
35.13	86213.67	108744.3	5179.81	12972.8	
35.88	82455	100080.7	969.2569	7029.528	
36.63	82700.33	104176	1729.231	5962.776	
37.38	79401.33	102384.7	3759.846	5280.476	
38.13	81025.33	103498.3	474.3589	7864.044	
38.88	77775	107246.3	3476.624	7440.688	
39.63	80133.33	103579.7	3319.326	9085.482	
40.38	76049.67	104761	2958.233	11294.22	
41.13	77112.67	100129	942.4868	8821.054	
41.88	78801	99482.33	2588.179	5109.508	
42.63	74089.67	100064.7	918.5882	4174.34	
43.38	76641.67	98822.67	3377.031	9069.322	
44.13	77061	100865	3638.95	5749.074	
44.88	76866.67	96654.67	2110.771	2861.065	
45.63	75758.67	99658	951.7402	8334.795	
46.38	74810.33	99216.33	1938.855	6912.912	
47.13	74379.33	95980.33	1064.021	1851.441	
47.88	74588.33	99823.33	3916.863	6490.109	
48.63	72074	98658	1976.187	5693.669	
49.38	71897	94111.33	2166.706	8122.427	
50.13	71718.67	96782	1396.56	4883.261	
50.88	71660.67	95875.67	1964.704	3873.831	
51.63	70058.33	96046.67	618.5065	5232.293	
52.38	71750.33	93970.67	882.0387	6394.161	
53.13	71452	94424.33	899.6505	6307.565	
53.88	68679.67	90626.33	3028.179	6242.705	
54.63	71725	94970	846.0573	7235.73	
55.38	69214.67	92535.67	1449.338	7562.594	
56.13	69161	93403	2954.427	6825.019	
56.88	68859.33	89464.67	3404.438	2870.256	
57.63	67673	90808.67	2160.688	7763.001	
58.38	68491.33	91669.67	1848.072	3316.783	
59.13	67209.67	88581.67	1113.571	5233.814	
59.88	66858.67	91531.67	285.2023	3263.341	
60.63	66703.67	88446	2269.896	7777.781	
61.38	64216.33	87356.67	3028.527	5847.561	
62.13	66391	88146.67	2494.164	1716.721	
62.88	65290.67	87128	459.6274	7798.052	
63.63	64246	84239.33	1555.39	4279.103	
64.38	64219	87211.33	543.5412	5323.339	
65.13	63005.67	87060	2987.286	6543.63	
65.88	63475	88873.33	1776.604	6503.686	
66.63	62155	87120.67	1515.544	7651.113	
67.38	63397	86228.67	3271.955	5948.478	
68.13	60009	86359.33	2012.462	7996.985	
68.88	60213	85806.67	2246.659	7502.261	
69.63	59257.33	84598.67	979.3448	5484.885	
70.38	60659.33	83932.67	3135.019	4436.447	
71.13	60129	82027.33	1274.588	6591.533	

Fig 5D. V3

(n=3)	Mean		S.D.		Statistical method used
Time(min)	SDF1α	Veh.	SDF1α	Veh.	unpaired Student's t-test
0	11432.67	10354.33	1040.889	757.0002	
0.75	12376.67	10830	1189.024	294.9593	
1.5	12545.67	11208	1321.245	1585.417	
2.25	12261.67	11029	1072.309	1247.829	
3	11996.67	11026.67	2269.846	1265.003	
3.75	11958.67	10840.67	1679.941	288.4741	
4.5	12178	11501	331.1842	1387.502	
5.25	12048.33	11728	1243.904	249.866	
6	12513.67	10649	1267.206	260.8812	
6.75	12638.33	11706.67	1069.225	1543.297	

7.5	11850	10863.33	1383.01	1060.753	
8.25	12121.67	10554.67	1266.203	1234.747	
9	11833.67	10366.67	1262.308	1412.496	
9.75	10636.33	11064	1386.569	1092.945	
11.13	10131	9505.333	1394.436	182.4673	
11.88	10068	10073.33	908.2637	1252.104	
12.63	10649.33	8639.333	619.0188	1665.829	
13.38	9489.333	9369.667	704.0386	391.6789	
14.13	10331	9665	1742.409	438.6377	
14.88	9777	8541.667	1069.104	1094.192	
15.63	10038.67	9347	2249.101	901.8054	
16.38	9645.333	9850	1631.571	655.2168	
17.13	10230.33	9206	1135.337	64.62971	
17.88	9676.333	9172	603.7295	598.9499	
18.63	9420	8870.667	641.5606	537.6359	
19.38	9607.333	9053.667	1725.803	874.7093	
20.13	9296.667	9243.667	1130.438	731.7652	
20.88	8656	9468.333	936.5741	551.7629	
21.63	9213	8698.333	1525.235	450.2003	
22.38	9667.333	8390.667	1227.044	1121.232	
23.13	9788.667	8459.333	674.1975	841.4876	
23.88	9245.667	8743	1390.498	678.891	
24.63	9320	9349.333	761.5911	533.4345	
25.38	8860	9260	972.7841	478.0031	
26.13	9330.333	8884.667	1081.104	720.4209	
26.88	9518.667	8585	1264.825	504.6315	
27.63	9093	8464.333	464.2973	568.8711	
28.38	8760	8012	1414.666	1190.684	
29.13	8592.667	8480.667	633.5253	1155.858	
29.88	8641.333	7575.333	1356.951	1037.928	
30.63	8820	8448.667	512.9591	459.2519	
31.38	8712.667	8435.333	1649.039	855.9126	
32.13	7872.333	7230.333	572.9314	922.0956	
32.88	8518	8423.333	945.0561	473.9677	
33.63	7953	6944.667	674.3063	1021.773	
34.38	8938.667	7565.333	903.3639	999.5701	
35.13	7787	8685.667	845.0793	1510.026	
35.88	7809.333	7302.333	326.5415	846.6524	
36.63	8440	7822.333	851.467	762.2836	
37.38	8028.333	7459.333	1682.844	348.1556	
38.13	7636.667	8767	1133.773	1073.106	
38.88	7782.333	7216.667	1452.635	788.7727	
39.63	7882	7605.333	527.4552	634.7947	
40.38	7479.667	7896.667	164.8343	1485.766	
41.13	7832.333	7747.667	412.2249	1038.879	
41.88	7852.333	7000	1293.11	912.4686	
42.63	6942	7055.667	598.8389	369.2659	
43.38	7190.333	6826	1532.783	369.1761	
44.13	7667	7539.333	130.679	307.2626	
44.88	7249	7754.667	847.1606	575.0499	
45.63	7661.333	6647	691.3771	898.4342	
46.38	6816	6906.333	585.7414	495.6706	
47.13	6828	6705.333	176.5701	808.3751	
47.88	7418.333	5877	767.3684	702.4294	
48.63	7316	7303.667	1419.751	695.0197	
49.38	7334	7069.333	988.8417	502.894	
50.13	7054.667	6149.667	445.9981	547.3631	
50.88	6940.333	6422.333	637.4044	984.1932	
51.63	6964	6523.667	686.9563	610.0511	
52.38	6639.333	6792	1422.674	911.3227	
53.13	7320.667	6953.667	935.9751	212.0786	
53.88	6465.667	6696.333	828.0008	650.8996	
54.63	6843.333	6110	458.8315	316.5865	
55.38	6626.333	6334.667	774.2153	421.5084	
56.13	6674.667	5916.333	264.2051	362.8227	

56.88	6736.333	6595	1100.536	1209.013	
57.63	6920	6351.667	874.6108	278.4624	
58.38	6440	6180.667	760.7845	750.0896	
59.13	6483	6215.667	796.1526	328.7344	
59.88	6795	5739.667	822.6008	212.9656	
60.63	6377.667	6231.667	906.3164	782.114	
61.38	6618	5850.333	450.4897	1459.412	
62.13	5553.667	5793.667	399.5201	516.9181	
62.88	6576.333	6490.333	443.7435	593.6003	
63.63	6280	5635.667	541.3677	223.5315	
64.38	6115	5676.667	418.7708	459.0385	
65.13	6271.667	5857.333	77.79674	215.3099	
65.88	6793.667	5834.667	770.5954	246.346	
66.63	5898	5595.333	671.9405	474.9151	
67.38	6156.333	5847.667	551.3187	657.3525	
68.13	5655.667	5376	136.5955	486.621	
68.88	5818.667	6191.667	679.001	76.69637	
69.63	6276.333	6063.667	1374.525	539.5946	
70.38	6194.667	5401.667	1310.65	699.1032	
71.13	5866.333	5247.667	677.8306	589.785	
**Fig 5D. V4**					
(n=3)	Mean		S.D.		Statistical method used
Time(min)	SDF1α	Veh.	SDF1α	Veh.	unpaired Student's t-test
0	18808	18223.33	2397.501	1141.176	
0.75	19774.33	19596.67	1708.785	2024.953	
1.5	19852.33	17985.67	1568.759	1667.685	
2.25	19436	19327.33	3086.75	2171.848	
3	18882.33	18969.33	3278.908	870.5023	
3.75	19705.33	18412	3334.283	1181.843	
4.5	19471.67	18208.67	3125.855	1633.951	
5.25	18992.33	18030.67	2844.679	1396.135	
6	18177	17896.33	3150.208	1529.384	
6.75	18815	18361.67	2585.959	2556.239	
7.5	18774.67	18090.67	2699.599	1560.25	
8.25	18252	17450	2282.178	1564.724	
9	16970.33	16410	606.9871	1218.685	
9.75	18403	17452.33	1188.255	2344.968	
11.13	16428.67	15068.67	3093.286	218.1292	
11.88	17334.67	14892.67	1327.457	1127.501	
12.63	17000.67	15783.67	2326.993	848.8912	
13.38	15062.33	14966	1927.94	2162.963	
14.13	16311.33	15537.67	1707.95	260.0968	
14.88	15929.67	14108	2358.886	1272.986	
15.63	15203.67	15060.33	2397.869	1545.209	
16.38	15860.33	14526.33	1900.9	1267.117	
17.13	15114	14433.67	1845.408	1794.532	
17.88	15576.33	13756.33	1785.292	450.0648	
18.63	15058.67	14732.33	1900.995	1814.944	
19.38	14700.33	15121	2919.178	190.1263	
20.13	14809.33	15230.33	2666.865	1967.738	
20.88	15472.33	14116.67	2240.536	1582.002	
21.63	15501.67	14168.67	2068.09	1746.305	
22.38	15223.67	13359.67	1925.782	713.3837	
23.13	14283.33	14427.67	827.2662	1307.625	
23.88	15378.67	13981	2058.452	1391.383	
24.63	15642.67	13389.67	1140.402	1190.074	
25.38	15154.33	13644.33	1895.441	885.3329	
26.13	14243	14016	2404.55	1399.472	
26.88	14474.67	13095	1834.717	2081.001	
27.63	14248.67	13760	1611.44	1005.271	
28.38	14036	13415.33	1266.451	856.444	
29.13	13134	13333.67	913.8583	1074.401	
29.88	13482.33	12423.33	1817.834	1263.288	
30.63	13560	13111.67	1062.503	1111.433	
31.38	12896	12176.67	986.3483	533.6378	

32.13	13927	12533.33	2388.604	985.2687	
32.88	13778.67	12617	2137.308	756.1131	
33.63	13356.33	13041.33	2147.197	2110.093	
34.38	13261	12594.33	2266.288	886.7583	
35.13	13153.67	12457.33	1777.055	1393.31	
35.88	12925.67	11820	1475.643	512.5973	
36.63	12107	12243	1136.279	913.3083	
37.38	13465.33	12618.67	2809.782	409.3426	
38.13	12904.33	11813.67	2015.171	1144.092	
38.88	13146	12266	1621.508	1129.454	
39.63	12499.67	11154.33	1390.782	594.9759	
40.38	13306	11709.67	1979.579	549.7575	
41.13	12146.33	12012.67	1653.354	1125.315	
41.88	11570.33	12099	2026.599	850.3652	
42.63	12602	12242.33	1944.12	854.3912	
43.38	11936	11996.67	877.0239	942.2157	
44.13	12603	12038.67	1996.548	1377.69	
44.88	12402.33	11428	2025.128	577.1265	
45.63	12288	11822.33	2080.26	519.2844	
46.38	11873	11917.67	1720.573	1146.866	
47.13	12323.33	10816.67	1470.084	1182.297	
47.88	11967.33	12018	1309.661	517.8571	
48.63	10769	11363.67	835.2227	1209.909	
49.38	11080.33	12426.33	1718.041	933.2279	
50.13	11660	11279.67	2171.92	432.6307	
50.88	12141.33	11367.67	1362.653	560.6642	
51.63	11478.33	10919.67	2072.042	613.5408	
52.38	10288.33	10921	1394.148	563.0124	
53.13	11350.67	11247.33	1420.186	1782.766	
53.88	10664.67	10767.67	1434.774	891.1747	
54.63	11927	10825.33	1108.078	940.5139	
55.38	10641.33	11215	1651.013	1101.417	
56.13	11059	10407.67	1187.973	698.5044	
56.88	10998.67	10721	1179.007	647.0093	
57.63	10504.33	10557	2612.112	1272.748	
58.38	11661.33	10104.67	1519.662	552.5951	
59.13	10906.67	10323	1533.919	640.8518	
59.88	11737.67	10763.67	3144.107	589.478	
60.63	10563	11045	1447.111	455.2373	
61.38	11055.33	10303	2080.679	535.3167	
62.13	11384.33	10768.67	2792.874	788.9286	
62.88	10598.33	10617.67	1466.497	495.1781	
63.63	9628	9946.667	1686.047	421.3198	
64.38	10483	10645.67	998.6636	671.3616	
65.13	9413	10206.67	346.5271	387.3039	
65.88	10286.67	9781	1779.5	1018.209	
66.63	9522	9255.667	372.0645	770.6194	
67.38	10328.67	10069	1310.604	650.4698	
68.13	9480.667	9870.667	889.8204	786.509	
68.88	10444	10824.33	1089.724	308.6265	
69.63	10192.67	9517	1421.704	1103.719	
70.38	10437	9725.333	375.3971	868.9076	
71.13	9995.333	9361.667	1746.266	1532.343	

Fig 5D. V5

(n=3)	Mean		S.D.		Statistical method used
Time(min)	SDF1α	Veh.	SDF1α	Veh.	unpaired Student's t-test
0	117696	110647	9352.039	4472.381	
0.75	119660.7	116428	6500.514	4527.002	
1.5	121805.3	116568.7	6185.682	3005.409	
2.25	110928.7	117956	6994.044	10202.22	
3	113661.7	118562.3	3872.121	12092.99	
3.75	120509.7	120725	3533.208	789.4865	
4.5	111183	116195.3	16029.98	4433.47	
5.25	116570	121989.7	5154.274	1380.406	
6	114237.3	123080.7	2472.904	11347.69	

6.75	111316.7	124028.7	3225.742	9382.327	
7.5	109675.3	120196.3	5188.472	11217.7	
8.25	110012.3	117156	4374.45	10963.32	
9	106836.3	117439.3	4649.609	4694.5	
9.75	110689.3	111490.7	9107.253	4090.217	
11.13	98947.67	97256.67	3898.45	6224	
11.88	92354.33	103396.3	878.3566	7134.193	
12.63	95225	97450.67	5488.164	7243.832	
13.38	93789.67	97772.67	736.1585	6995.342	
14.13	90146.67	94296.33	3253.374	3326.317	
14.88	93534	97015.33	5120.477	3096.262	
15.63	90340	99382.67	2522.542	3119.186	
16.38	90163	96296.33	814.0018	979.3581	
17.13	87335	92736.33	4426.055	5905.606	
17.88	87356.33	91021.67	1225.934	3932.475	
18.63	89325.33	93292.33	2808.905	1935.296	
19.38	87822.67	92634.67	4129.79	3901.572	
20.13	84716.67	93218.67	714.896	6983.467	
20.88	83910	90422.33	3430.843	3269.979	
21.63	83893	86161	3219.506	1915.535	
22.38	84809.67	90714	1823.107	1188.566	
23.13	82622.67	88923	2369.121	569.116	
23.88	80511.67	88871.33	1734.672	4080.063	
24.63	82584.67	86389.33	2909.871	1522.941	
25.38	81579.67	86453.67	4003.716	2148.887	
26.13	80617.33	86977.33	1539.045	1835.431	
26.88	78937	84170.67	1380.798	2106.267	
27.63	77514	85126	1118.079	3058.704	
28.38	80150.67	83064	1620.05	1260.365	
29.13	78212.33	86848	3698.868	1052.01	
29.88	78082.67	83365	2273.021	4683.853	
30.63	79758.67	83871.67	2280.151	8230.931	
31.38	77955.67	81066.67	1434.476	3111.604	
32.13	77153.33	82284.33	1769.165	4786.295	
32.88	74084.33	82829	2000.616	4262.074	
33.63	73917.67	82211.67	85.73409	2726.826	
34.38	75387.33	77949	4289.463	3739.932	
35.13	73685.67	79603.33	1597.569	3319.029	
35.88	73177.33	77565.67	1764.345	600.9329	
36.63	72179.33	78285.67	1846.087	2817.226	
37.38	71772.33	78082.67	1856.351	2929.871	
38.13	72712.33	78106	2907.8	4699.19	
38.88	71072	77445.67	2783.769	604.2933	
39.63	70627	77416	887.3173	2986.91	
40.38	71170.33	75296.33	1500.574	1540.822	
41.13	69533.67	77110	2832.445	4176.565	
41.88	70056	74781.33	1288.143	2415.64	
42.63	71592.33	73475.67	928.5264	3403.042	
43.38	68061.67	74293	3601.034	2343.483	
44.13	67687	73829.33	873.4552	1704.055	
44.88	66822.33	73214.67	2222.586	962.0989	
45.63	66511.67	72625.67	655.2872	612.2257	
46.38	66468.67	72596.67	757.8999	1730.08	
47.13	65368.67	71524.33	2687.319	1274.32	
47.88	64518	70317	870.6968	4928.514	
48.63	64549.33	71291.67	1889.724	1475.278	
49.38	64820	70957	2417.328	3284.628	
50.13	64411.67	70080.67	2287.08	2745.309	
50.88	63962	70682	978.6991	1492.101	
51.63	62175.67	68822.67	1462.415	2839.543	
52.38	61883	69878	2120.268	2622.345	
53.13	62030	67094.67	2814.682	1997.476	
53.88	61548.67	67802.33	2605.611	1508.31	
54.63	61248	66611	2773.019	1793.572	
55.38	61121	67276	2091.232	604.1912	

56.13	59701.33	66349.33	4326.449	2553.179	
56.88	59635.33	65738.33	3999.373	2242.007	
57.63	60372.67	67796.33	2587.201	2172.354	
58.38	58412.33	65575.67	1845.799	540.3557	
59.13	58000.67	64917.67	1011.289	3664.644	
59.88	59216.67	63296.33	2138.357	2518.566	
60.63	56076	65284.67	1190.115	3328.471	
61.38	57767	65764.33	2856.218	2348.414	
62.13	56853.33	62993.67	714.3734	3236.134	
62.88	55756	64975.67	643.7833	5964.047	
63.63	55166.67	64415	1638.287	518.2345	
64.38	53933.33	63794.67	1893.709	4832.562	
65.13	54552.67	59969.67	1878.906	3617.893	
65.88	56253.33	61809	1130.094	3822.214	
66.63	52535.33	61603.33	1505.963	2357.762	
67.38	55678	59836.67	615.8709	1040.19	
68.13	52237.67	61334.67	1137.299	1872.06	
68.88	53230.33	60475.67	878.9006	3207.312	
69.63	52134	59685.67	2691.177	1972.923	
70.38	52795.67	58478	2185.591	2624.252	
71.13	52948	58283.33	2008.525	1786.061	

**Fig 5E. V1**					
(n=3)	Mean		S.D.		Statistical method used
Time(min)	SDF1α	Veh.	SDF1α	Veh.	unpaired Student's t-test
0	8162.667	8150.333	940.4261	988.3979	
0.75	8471.667	8286.333	206.3234	1025.398	
1.5	8771.667	8548.333	302.3679	874.0379	
2.25	8884	9980.667	742.4345	1178.815	
3	9125.333	9296.333	624.809	503.2855	
3.75	9155	9163.667	509.5596	783.569	
4.5	9214.333	9399.667	476.4801	896.4543	
5.25	9606.333	8833	328.6721	961.7531	
6	9053.667	8972	289.5122	427.1054	
6.75	9799.333	9893.667	1000.193	1456.126	
7.5	8916.667	8696.667	111.5631	454.8344	
8.25	9080.667	8501	1060.045	1124.325	
9	9118.333	8961.667	768.664	434.4851	
9.75	8391.667	9182.667	932.7166	544.579	
11.83	6800	6144.333	710.6666	862.9741	
12.58	6023.333	5899.333	264.7156	632.2043	
13.33	7352.333	6199	709.9791	746.8139	
14.08	6454.333	5916	507.7424	264.456	
14.83	6646	6453	375.8085	1011.164	
15.58	7525.667	6123.667	263.504	1219.898	
16.33	7063	6678.667	597.9197	236.6904	
17.08	7025.333	6872.667	809.3296	1068.362	
17.83	7218	5914.667	39.68627	586.6049	
18.58	7327.333	6374.333	897.3875	1070.702	
19.33	6898	6001.333	1089.567	277.2189	
20.08	7311.667	6021	392.8872	619.9411	
20.83	6574.667	5428	673.0419	499.808	
21.58	6170.333	5912.667	483.3811	199.4049	
22.33	7197.333	6717.333	870.7137	611.0567	
23.08	6434.667	6522.667	637.293	742.6691	
23.83	6500.333	5929.667	490.7732	331.8197	
24.58	6270.667	5657.333	543.7686	538.2233	
25.33	6379.667	5667.333	230.9986	107.8208	
26.08	6778.667	5548.333	562.4147	361.1929	
26.83	5906	6095.667	513.3605	840.8307	
27.58	5791.667	5331.333	130.7147	1049.979	
28.33	5700.333	5378	301.4205	1171.546	
29.08	6236.333	5331.667	474.8603	501.4074	
29.83	6657	5721.667	1222.044	645.4815	
30.58	6235	5495.667	112.3032	728.4342	

31.33	6120.667	5493.667	1300.398	629.535	
32.08	6612.333	5173.667	360.335	455.2333	
32.83	6154.667	5176	426.7568	308.7863	
33.58	5349	5172.667	335.6963	265.583	
34.33	6230.667	5474.333	538.54	846.2307	
35.08	5588.667	5272.667	676.2435	194.0163	
35.83	5967.667	5424	1009.723	477.5385	
36.58	5258.333	5433.333	136.06	275.0715	
37.33	5881	5727	795.2968	703.7947	
38.08	5876	4980.667	535.9207	729.5439	
38.83	5389.667	4712	509.6767	432.5702	
39.58	5324.667	5061.333	428.1884	848.5413	
40.33	5731.333	4883.333	1121.089	349.1494	
41.08	5162	4730.333	413.3848	247.4214	
41.83	5158.667	4738	1129.737	486.735	
42.58	4915.667	4729.333	559.0012	348.6493	
43.33	4892	5559	723.1646	442.5732	
44.08	5499.333	4667.333	628.7268	584.8028	
44.83	4784.333	4848	451.6739	629.8222	
45.58	5171.667	4102.333	751.5593	688.609	
46.33	5738	4935	326.233	679.2886	
47.08	5293.667	4241.333	479.2018	288.5556	
47.83	4832.333	5128	640.422	329.2917	
48.58	4610	4404.667	556.903	785.2492	
49.33	5120.333	4285	573.9393	537.1369	
50.08	4810	4245.667	551.293	684.2254	
50.83	4681.333	4457	103.4714	762.5805	
51.58	4599.333	4514.333	526.6596	749.9402	
52.33	4428.333	4159	542.5075	397.6443	
53.08	4871.667	4052	328.5884	159.3393	
53.83	4663.667	3891.333	517.6237	1007.84	
54.58	4240.667	4119	147.8152	542.4011	
55.33	4150.333	3888.667	186.9501	299.2229	
56.08	4414	4475.333	560.7736	505.7631	
56.83	4171.333	3920	151.9616	440.8821	
57.58	4260.333	4102.333	400.5313	218.5963	
58.33	4232	3504.333	1415.03	440.8564	
59.08	3940.667	3645	208.4258	104.8475	
59.83	4137.667	3918.667	113.496	430.0167	
60.58	3737.667	3574	294.8327	204.0662	
61.33	4361	4182.667	808.1856	843.1099	
62.08	4403.333	3405.667	1017.783	505.86	
62.83	4185	3885	720.3895	467.4302	
63.58	3814.667	3318	188.6169	312.0401	
64.33	4101	3643	702.2272	567.1252	
65.08	3816.667	3484.333	806.4009	354.9216	
65.83	3862.667	3684.333	259.5503	410.7631	
66.58	3871.333	3480.667	345.1265	207.4134	
67.33	3859	3137.667	380.3314	415.4544	
68.08	3970	3602.667	336.4655	157.2938	
68.83	3536.333	3603.333	327.5093	598.7874	
69.58	4545	3706.667	289.4357	581.1509	
70.33	3671	3734	604.2011	281.3947	
71.08	3648.333	3369.333	87.52333	160.1135	
71.83	3426	3693.667	266.1353	653.0707	
**Fig 5E. V2**					
(n=3)	Mean		S.D.		Statistical method used
Time(min)	SDF1α	Veh.	SDF1α	Veh.	unpaired Student's t-test
0	22758.67	23612	3961.472	4276.047	
0.75	23085.33	24220	3396.564	3670.595	
1.5	25506.67	25201.33	3008.165	4135.543	
2.25	24527	27164.33	4252.199	3820.021	
3	24878.33	26661.67	2844.73	5136.858	
3.75	24568.33	26656	2508.963	5351.65	
4.5	23150.33	26791	3074.593	4110.689	

5.25	24006.67	25536.33	3188.091	3622.363	
6	24662	26981.33	3790.708	4176.239	
6.75	23457.67	26169.67	2482.442	5860.871	
7.5	22721.67	25652.33	3238.151	4743.489	
8.25	23943	24935.33	3695.609	3409.927	
9	22891.33	24210.67	3510.799	3220.375	
9.75	23181	23007.33	2790.059	4261	
11.83	18084	18889.33	1424.493	1867.922	
12.58	17707	17697.33	2890.929	4648.001	
13.33	17679	19345.67	2389.131	3944.596	
14.08	18246.33	18489.67	3541.481	3678.714	
14.83	17706	19130.67	594.4039	2710.115	
15.58	18058.67	17963.67	2988.87	1851.767	
16.33	20790.33	19117.33	3000.006	3736.826	
17.08	19171.33	18412	3170.982	3467.258	
17.83	20457	18954	2642.273	3079.083	
18.58	20236.33	17855.33	3025.647	1267.851	
19.33	20201.67	18204.33	2927.297	2980.777	
20.08	20307.67	17579.67	2480.909	2204.167	
20.83	19507.67	19373.67	1861.506	2686.718	
21.58	20824.33	18139.33	2292.001	1358.13	
22.33	20402	17909	4310.465	3133.98	
23.08	20806.33	17233.67	3967.433	2448.696	
23.83	20188	18594.67	4094.887	3504.509	
24.58	20854.33	17535.67	2569.669	1286.905	
25.33	20324.33	16661	3084.737	1773.302	
26.08	21249.33	17421.67	2794.836	3516.18	
26.83	21538	16964.67	3718.004	3377.522	
27.58	19852.67	16221.33	2530.613	2680.138	
28.33	20527.33	16938	2055.367	3468.447	
29.08	20732.67	16752.67	2906.366	3114.644	
29.83	20867.33	16221	1878.679	2637.729	
30.58	20231.33	17229	2170.069	2446.042	
31.33	20224	16305	4433.164	3164.863	
32.08	20107.33	15942.33	1635.644	3501.066	
32.83	20063.67	16426	2641.251	3294.241	
33.58	20200.67	14772.33	3119.362	2941.258	
34.33	19995	14891	2331.032	2149.867	
35.08	21061	16278.33	2836.746	2490.49	
35.83	21795.33	14629.67	2239.078	2697.834	
36.58	20227.67	15615.33	2674.788	2957.645	
37.33	18483	14509.67	2830.992	2880.95	
38.08	20364	14553.67	3361.582	2084.904	
38.83	21060.33	14966	3437.976	1438.187	
39.58	19116.67	15041	735.2322	2102.587	
40.33	20560.33	14767	2227.632	1721.76	
41.08	20817	14107	2618.182	2250.353	
41.83	18855	13667.33	3928.468	1322.254	
42.58	18333.67	13676.33	2379.131	2591.564	
43.33	20422.67	13281.33	2381.269	3393.918	
44.08	19465	14354.67	2790.01	3305.018	
44.83	17928.33	13504.33	2548.168	2275.568	
45.58	19150.67	13877.67	2585.907	1919.63	
46.33	19059.67	13070	2677.378	3018.463	
47.08	20222.67	13122.33	2260.518	2302.994	
47.83	18519.33	13057	2638.163	3105.474	
48.58	18215	12751.67	3481.189	2231.655	
49.33	18106	12880.67	2506.665	1802.489	
50.08	18279.67	12856.33	3647.573	1553.842	
50.83	18223.67	12207.33	2000.668	2500.914	
51.58	18870	12161	1501.049	1377.756	
52.33	18975.33	12517.67	2221.185	2166.127	
53.08	17865.33	13177.33	2120.171	1826.06	
53.83	17378	12253.33	3229.656	2411.907	
54.58	18923	12653.33	2332.882	1736.825	

55.33	18815	12716	3201.151	2006.478	
56.08	17244.67	11671	2520.108	2197.173	
56.83	17171.67	12606	1507.721	2206.111	
57.58	16097.33	11750.33	1484.234	1517.535	
58.33	16479.33	12740.33	1444.055	2471.385	
59.08	16734.33	11962.67	1465.608	1556.656	
59.83	17129	11656.67	2485.871	2392.967	
60.58	16323.67	10534.33	3047.099	1264.579	
61.33	17325	11581.33	2983.826	1415.735	
62.08	15862	11382	2775.181	2866.36	
62.83	16331.67	10982.67	1774.712	1679.341	
63.58	15358.67	10089.33	1679.563	1848.448	
64.33	14933.67	11012.33	2403.189	2287.775	
65.08	16181.67	10306.33	2332.969	2068.954	
65.83	15069.33	11134	2456.874	1992.642	
66.58	15798.67	11900	2180.814	1240.662	
67.33	15570.33	11485.33	2802.038	1753.684	
68.08	15451	10474.33	1547.931	2413.712	
68.83	16709.67	10272.33	2776.21	2501.124	
69.58	16150.33	9868	2729.051	1924.105	
70.33	15536.33	11013	3525.086	801.6315	
71.08	14977.67	10909.33	2369.91	1995.858	
71.83	15716.33	10673.33	1646.413	2529.303	

Fig 5E. V3

(n=3)	Mean		S.D.		Statistical method used
Time(min)	SDF1α	Veh.	SDF1α	Veh.	unpaired Student's t-test
0	2462	2240.667	156.9044	236.3902	
0.75	2492.667	2224.667	194.1039	154.9914	
1.5	2695.333	2866.333	660.1578	627.3614	
2.25	2882.667	2486	370.6431	116.0517	
3	2820.333	2797.333	625.8549	621.41	
3.75	2831.667	2941.667	255.2496	295.2039	
4.5	2600	3021.333	238.7614	339.5384	
5.25	2627.667	2525	398.8889	348.7363	
6	2371	2479.667	516.4301	599.4542	
6.75	2308.333	3003.667	576.4524	616.1025	
7.5	2447.667	2516.667	377.7437	469.7428	
8.25	2696.333	2255	379.6898	442.6906	
9	2196.333	2480.667	551.3586	396.3096	
9.75	1943	2432.333	124.8519	82.39741	
11.83	2142	2088.667	223.2398	356.3276	
12.58	1924.333	2122.667	378.093	131.2263	
13.33	2123.667	1613.667	430.8321	38.63073	
14.08	1892.667	1954	661.2309	396.0164	
14.83	2121	1736.667	265.5541	424.5354	
15.58	1599.333	1998.667	517.0206	436.8734	
16.33	1762.667	1736.333	318.4734	109.6555	
17.08	2137.667	2048.667	296.4664	343.1535	
17.83	1676	1569.667	299.0435	322.0813	
18.58	1873.333	1838	358.1429	246.2438	
19.33	1961.667	2004.667	118.7111	139.321	
20.08	1842	2048.667	130.2881	766.4818	
20.83	1728.333	2072.667	323.7381	765.9245	
21.58	2099.667	1920.667	264.6835	294.8175	
22.33	1961	1859.333	301.841	110.2104	
23.08	1743	1930.333	535.8908	363.4781	
23.83	2081	1747.333	80.07496	292.5104	
24.58	1680	1988.667	389.7781	557.6471	
25.33	1831.667	1903.333	413.8772	296.5035	
26.08	1966.667	1974	665.802	572.3775	
26.83	1953.667	1708	410.6779	248.5136	
27.58	1619.333	1804	441.2735	108.282	
28.33	2094	1886.667	356.8207	237.0998	
29.08	1655.333	1583.333	42.71222	524.3723	
29.83	1628.333	2030.667	144.4241	48.27353	

30.58	1894.333	1981	174.9209	417.5009	
31.33	1857.333	1494	434.5185	310.889	
32.08	2007.333	1704	82.58531	200.5293	
32.83	1660.667	1927.667	33.29164	255.1477	
33.58	1924	1799.667	375.461	357.0915	
34.33	1497	1650.333	183.1912	183.6146	
35.08	2033.333	1791.667	427.3246	430.6104	
35.83	1638.333	1975.333	378.5028	370.689	
36.58	1443.333	2030.333	367.579	450.7442	
37.33	1535	1697.333	84.25556	141.6204	
38.08	1552.667	1620	274.9861	119.0252	
38.83	1675	1640.333	149.2213	241.5209	
39.58	1454.667	1667.667	234.5684	429.5932	
40.33	2189.667	1585.333	102.5736	416.9464	
41.08	1603.667	1977	402.0278	106.972	
41.83	1507.333	1527.667	129.1601	465.1734	
42.58	1617	1651.333	197.4411	211.0695	
43.33	1481.667	1917	207.1867	716.0398	
44.08	1582	1604	101.484	223.5017	
44.83	1302.667	1335.333	130.0205	324.2042	
45.58	1488.667	1496.333	267.1073	136.4563	
46.33	1694	1552.667	312.5012	218.0283	
47.08	1707.333	1283.667	449.2108	397.6812	
47.83	1655.667	1325.667	143.3504	293.0774	
48.58	1370.333	1337.667	153.5719	164.1168	
49.33	1517	1588.667	581.1136	302.0138	
50.08	1353.667	1598.667	263.0406	137.5512	
50.83	1184	1413.667	180.6322	132.2283	
51.58	1511	1489.667	681.1923	127.3787	
52.33	1401	1340	195.7779	308.7539	
53.08	1576.333	1406.333	194.6724	326.0327	
53.83	1454.667	1313.333	269.7598	404.2689	
54.58	1350	1214.667	236.8882	280.304	
55.33	1712.333	1446.667	692.8725	199.623	
56.08	1526.667	1401.667	163.4452	123.8803	
56.83	1237.333	1314.667	78.39855	153.7216	
57.58	1628	1500.333	302.9472	110.6541	
58.33	1398	1284	304.8918	233.0064	
59.08	1292.667	1122	256.5937	209.2152	
59.83	1368	1311.667	175.6331	117.0997	
60.58	1490	1375.333	186.1532	245.1863	
61.33	970.6667	1316	150.4405	294.5318	
62.08	1191	1267.667	187	336.1805	
62.83	1349.333	941.6667	131.6713	202.1245	
63.58	1361.333	1226.667	197.0008	297.9418	
64.33	1319.667	1018.333	334.222	266.5358	
65.08	1222	1362.333	251.7558	563.7893	
65.83	1067	1115.667	223.723	474.3726	
66.58	1431.667	1226.333	217.1965	226.7804	
67.33	1352.333	1334.667	199.0888	92.65168	
68.08	1435.333	1471	15.27525	132.5028	
68.83	1281	1288.333	430.1081	461.76	
69.58	1172.333	1311.333	205.1349	352.4292	
70.33	1432.667	1356	335.7027	116.1551	
71.08	1100.333	1082.667	298.2991	327.3077	
71.83	1300.667	1357.333	330.4396	172.7841	
**Fig 5E. V4**					
(n=3)	Mean		S.D.		Statistical method used
Time(min)	SDF1α	Veh.	SDF1α	Veh.	unpaired Student's t-test
0	3230	3243.333	577.4781	125.8345	
0.75	2973.667	3230.333	666.5743	58.07179	
1.5	3475.667	3777.333	701.6048	701.7623	
2.25	3559	3612.667	612.3161	611.6652	
3	3955.667	2955.333	508.9915	515.5825	
3.75	3463	3438.333	221.2487	276.2197	

4.5	3293	3669.667	768.4354	377.2139	
5.25	3279	3401	184.0136	618.0388	
6	3742.333	3097.667	290.5931	642.0797	
6.75	2946	3376.333	156.6142	383.0096	
7.5	3343	3321	734.0715	1007.189	
8.25	3325	3077	531.2655	705.526	
9	3321	3422.333	540.3027	825.1135	
9.75	3419.667	3225.333	640.019	313.4922	
11.83	2700	2371.667	168	301.3011	
12.58	2254.333	2687	193.4537	327.6156	
13.33	2361.333	2624.333	690.5334	439.582	
14.08	2744	2356.667	551.1552	65.1639	
14.83	2305.333	2465.333	295.358	446.0688	
15.58	2470.333	2310	222.3428	693.2409	
16.33	2463.333	2825	637.9352	838.7157	
17.08	2519.333	2730.667	727.1275	513.4212	
17.83	2655.667	2911.333	169.8156	174.1848	
18.58	2214	2595.667	104.2161	276.3012	
19.33	2575.667	2496	64.5936	189.6128	
20.08	2308.667	2318.667	208.2699	269.3888	
20.83	2469.667	2223	119.8012	395.6602	
21.58	2853.667	2607.667	98.19029	602.1705	
22.33	1995.333	2809.667	316.9926	380.9913	
23.08	2744	2448.667	508.8192	383.0892	
23.83	2205.667	2633	273.4599	249.3271	
24.58	2181	3053	186.4189	243.4153	
25.33	2520.333	2536.667	567.0285	485.0055	
26.08	2340.333	2638.667	204.4146	340.2886	
26.83	2065.667	2478	473.1134	273.1447	
27.58	2352.667	2122	225.9875	150.09	
28.33	2140	2165.333	459.4736	509.3097	
29.08	2633.667	2232	402.1745	306.5338	
29.83	2165	2213.667	123.1584	373.168	
30.58	1988	2204.667	118.8823	216.9731	
31.33	1916.333	2147.333	584.6848	396.5732	
32.08	2758	1962.333	274.5779	398.3646	
32.83	2397.667	2119.667	432.5105	362.5553	
33.58	2148.333	2307.333	496.9168	311.5146	
34.33	1913	2328.667	272.7251	459.6709	
35.08	2285	2071	473.9188	304.8672	
35.83	2400	2431	334.6222	192.7589	
36.58	2317	2324.667	92.2605	304.6594	
37.33	1599.667	2438.667	156.3213	418.4619	
38.08	1956	1644.333	154.2044	222.8034	
38.83	1787	2181	181.8488	489.8377	
39.58	2162	1853	121.1487	99.41328	
40.33	1813.333	1941	232.6808	328.6533	
41.08	2159.667	2023	136.6102	283.1325	
41.83	2023.333	1580.667	268.3903	273.6518	
42.58	1909.333	2009.667	437.8691	380.7234	
43.33	1828	1979.333	392.0038	165.3612	
44.08	2188.333	1932.333	60.47589	85.17237	
44.83	1928.667	1869	437.5001	315.8433	
45.58	2018	1819	315.073	510.1882	
46.33	1798	1956.333	171.8226	160.4504	
47.08	2151.333	1896.667	218.77	391.3545	
47.83	1694.333	2107	497.4599	96.5971	
48.58	1665	2073.667	187.0936	177.207	
49.33	1846	1625	396.3067	169.8853	
50.08	1826	1793.333	233.0064	189.4659	
50.83	1970	1900.667	534.4614	253.2377	
51.58	1811.333	1907.333	419.1436	424.314	
52.33	1970.667	1849	312.3609	278.9319	
53.08	1938.333	1790.667	130.9364	264.7269	
53.83	1641	1909.333	299.3209	304.3063	

54.58	1700.667	1849	144.7976	295.4708	
55.33	1883.333	1789	158.0833	87.42997	
56.08	1704	1388	243.7847	178.1348	
56.83	2080.667	2051.333	456.8658	373.5322	
57.58	1579.667	1565	248.2425	309.3267	
58.33	1761.333	1827.667	181.3955	417.0783	
59.08	1827.333	1750.333	24.13158	367.6062	
59.83	1736.667	1713.667	394.8042	185.5811	
60.58	1554.667	1670	102.9093	198.1893	
61.33	1566.333	1827	333.5031	519.9394	
62.08	1743.333	1564	161.9763	363.3965	
62.83	2019.667	1623.333	109.0245	77.29381	
63.58	1439.667	1631.333	362.8462	398.4587	
64.33	1787.333	1669.667	276.5797	308.5212	
65.08	1629.667	1822.333	414.5363	264.6627	
65.83	1702.333	1405.667	564.3406	155.7733	
66.58	1930.333	1563	662.414	190.8743	
67.33	1648.667	1768.667	404.7534	117.7512	
68.08	1589	1520	141.5203	298.2264	
68.83	1614.333	1591.333	302.5795	325.2327	
69.58	1406.667	1777.333	227.0162	343.4666	
70.33	1325.333	1922	130.7682	604.6396	
71.08	1556	1847	379.7117	260.8294	
71.83	1326.667	1812	501.2039	224.7065	
**Fig 5E. V5**					
(n=3)	Mean		S.D.		Statistical method used
Time(min)	SDF1α	Veh.	SDF1α	Veh.	unpaired Student's t-test
0	13544.33	11690	1701.274	1213.702	
0.75	13218	12776.33	1173.274	1315.381	
1.5	13285.33	13405.67	1849.542	720.01	
2.25	13050.33	12731.67	86.40795	1205.559	
3	13847.67	12700.33	1465.432	940.4745	
3.75	13634.67	12678.33	1244.794	1441.549	
4.5	13290	12716.33	1023.947	1155.065	
5.25	13451.33	12729.67	210.3077	631.0343	
6	13130.33	12987	772.7188	1510.627	
6.75	12658.33	12714.33	1011.004	1162.999	
7.5	12002.67	12975.67	829.3035	1180.892	
8.25	12012.67	12023.67	837.5114	1178.192	
9	11971	12187.67	1082.104	1278.293	
9.75	12723.67	12236.67	657.4909	723.8966	
11.83	9660	7350	889.0827	1203.244	
12.58	9675	8654.667	496.8974	1024.765	
13.33	10540.33	7696.333	950.0928	133.1928	
14.08	9353	7679.667	916.7922	440.3571	
14.83	9991	7583.333	695.6529	892.6037	
15.58	8861	8425.333	243.9324	210.5239	
16.33	9239.333	8431	1367.152	223.1076	
17.08	9297.333	7847.333	690.537	489.2958	
17.83	8834.333	9290.667	466.9961	810.1113	
18.58	9299	9325.333	717.4671	297.653	
19.33	9510	8250.333	470.8418	488.3568	
20.08	8809	8675.667	1122.164	881.0564	
20.83	8428.667	8360.667	962.6839	642.2658	
21.58	8486	9160.667	987.1358	300.377	
22.33	8588.667	8048	313.5001	120.7974	
23.08	8376.667	8153	975.172	1320.467	
23.83	8124.333	8561	404.4828	408.7689	
24.58	8556.333	8009.333	557.4893	711.805	
25.33	8472.333	8670.333	130.2165	620.1325	
26.08	8007.333	8128	511.8304	371.7835	
26.83	7699	8231.333	932.6398	996.0002	
27.58	7786.333	7951.333	595.2389	722.2896	
28.33	8124.667	8717	910.0584	265.0057	
29.08	8358	7322.333	635.0283	505.7295	

29.83	7668	7601.333	504.2807	229.1666	
30.58	6907.667	7976	334.9587	1121.226	
31.33	7812.333	7181.667	583.4229	683.5381	
32.08	7140.333	7541.333	1166.899	516.8427	
32.83	6906.333	7558.333	560.7284	243.0665	
33.58	6934.667	8294	214.0475	642.2437	
34.33	7561.333	7412.667	78.85007	195.8579	
35.08	6401	6642	708.0388	297.6222	
35.83	6744.333	7674.333	647.0489	1169.018	
36.58	6395	7454.667	259.2084	410.0663	
37.33	6910	7372	474.9863	903.7013	
38.08	6540.667	7822	874.0494	551.633	
38.83	6796.333	7097	378.2252	668.6913	
39.58	6689.333	6706.333	180.9346	420.0099	
40.33	6678.667	7310.667	852.7469	659.5827	
41.08	7014.667	7415.667	403.1331	730.2262	
41.83	6744.667	6589.667	874.5252	527.1436	
42.58	5856.333	6094.333	666.8885	335.7832	
43.33	6262.333	6460	461.4069	1001.605	
44.08	5969	6678.667	510.4714	110.6451	
44.83	6690.333	6107.333	194.6518	402.5374	
45.58	6266	6078.333	482.1794	358.7343	
46.33	6053	7089	1394.707	817.413	
47.08	6229.667	6783.667	340.2739	78.0534	
47.83	5396	5566.667	674.0022	236.5298	
48.58	6080	6475.333	1287.78	383.5118	
49.33	6228.333	6002	430.105	406.9042	
50.08	5310	5682.667	1062.874	460.7519	
50.83	5785.333	6245.333	1231.818	289.1096	
51.58	5456.333	5799	302.3976	372.7895	
52.33	5335.667	5942.667	334.4343	241.5312	
53.08	5355.667	5543	1132.937	448.0134	
53.83	5459	5371.667	661.0832	455.4957	
54.58	5383	5871.667	274.1314	398.6682	
55.33	4970.333	5647.333	619.5259	243.8531	
56.08	5353.333	5292	534.4963	401.9341	
56.83	5769.333	5536.667	616.7952	775.3401	
57.58	5273.667	5704	725.1209	665.212	
58.33	5702.333	5774	781.157	573.396	
59.08	5188	5686	554.9658	30.51229	
59.83	4776	5667.333	603.9727	481.8738	
60.58	4899.333	5491.667	235.9181	626.7985	
61.33	4466	5270.667	404.5145	246.6279	
62.08	4701.333	4786	1096.427	616.2605	
62.83	4872	4786	345.6487	195.123	
63.58	4953	5019.667	178.6813	75.40778	
64.33	4349	5356.667	468.7814	231.4613	
65.08	4490.667	5054.333	95.03859	428.1195	
65.83	5032.667	4957.667	426.6337	247.0675	
66.58	4766.667	5134	338.5415	199.7298	
67.33	4542.667	5628.333	1066.543	597.4658	
68.08	4594.333	5097	128.8578	752.2952	
68.83	4583.667	4540	320.1442	511.0841	
69.58	4605.667	4859	305.6539	228.6307	
70.33	4515.333	5241.333	253.8352	387.4072	
71.08	4312	5188	404.1819	652.0828	
71.83	4619.667	5042.667	365.9649	248.0087	

(n=3)	Mean	S.D.	Statistical method used	P value
**Fig 6A**			unpaired Student's t-test	**p<0.01<br>*p<0.05
Vec.	860.92	188.0842683		
V1	100448.96	12262.72174		
V2	193123.4	29630.1938		
V3	4475.88	1290.263652		
V4	14980.28	3234.857461		

V5	123962.48	8017.999592		

Fig 6C. UbiC V2+Vector					
(n=3)	Mean		S.D.		Statistical method used
Time(min)	SDF1α	Veh.	SDF1α	Veh.	unpaired Student's t-test
0	1.030093	1.090548	0.075174	0.019553	
0.98	1.0196	1.102116	0.048559	0.019404	
1.96	1.034009	1.148287	0.044909	0.110318	
2.94	1.046856	1.104232	0.003286	0.045802	
3.92	1.051937	1.074345	0.016959	0.028339	
4.9	1.186816	1.073326	0.195863	0.011943	
5.88	1.051253	1.098014	0.049261	0.100891	
6.86	1.081199	1.030512	0.102814	0.014899	
7.84	1.036565	1.020217	0.01767	0.005806	
8.82	1	1	0	0	
10.65	3.997794	0.918877	0.716815	0.010694	
11.63	3.949045	0.889606	0.406392	0.005993	
12.61	3.369768	0.942401	0.400727	0.095748	
13.59	2.564087	0.911864	0.19745	0.010676	
14.57	2.467179	0.873395	0.197756	0.010127	
15.55	2.05158	0.872589	0.243529	0.031174	
16.53	2.043838	0.84126	0.102265	0.009153	
17.51	1.802709	0.832166	0.114555	0.016937	
18.49	1.509362	0.84826	0.043302	0.067171	
19.47	1.550627	0.824729	0.166568	0.036078	
20.45	1.549429	0.798034	0.064086	0.026286	
21.43	1.442834	0.778984	0.133524	0.017064	
22.41	1.381665	0.784342	0.079974	0.02583	
23.39	1.336754	0.75981	0.119981	0.023672	
24.37	1.292342	0.76166	0.113087	0.019523	
25.35	1.226905	0.792014	0.184883	0.088506	
26.33	1.255129	0.880262	0.163628	0.22804	
27.31	1.167807	0.811614	0.028035	0.091575	
28.29	1.0507	0.740818	0.089236	0.04593	
29.27	1.019751	0.724831	0.076728	0.026268	
30.25	0.957547	0.757531	0.036653	0.067327	
31.23	1.00883	0.720111	0.026229	0.034886	
32.21	0.994182	0.73941	0.04934	0.070532	
33.19	0.919751	0.702252	0.059992	0.029267	
34.17	0.979915	0.718825	0.172637	0.063002	
35.15	0.963534	0.676429	0.03022	0.031196	
36.13	0.850033	0.67418	0.036181	0.039832	
37.11	0.861334	0.666596	0.023889	0.043355	
38.09	0.818445	0.697357	0.024705	0.032537	
39.07	0.84496	0.674125	0.085898	0.02461	
40.05	0.800873	0.662195	0.020505	0.040563	
Fig 6C. V2+V1					
(n=3)	Mean		S.D.		Statistical method used
Time(min)	SDF1α	Veh.	SDF1α	Veh.	unpaired Student's t-test
0	0.968293	0.967069	0.05332	0.033414	
0.98	1.030932	1.012286	0.023207	0.032904	
1.96	1.041452	1.063816	0.020761	0.050797	
2.94	1.062534	1.055654	0.021495	0.032323	
3.92	1.063576	1.058811	0.023877	0.032061	
4.9	1.053886	1.036349	0.012048	0.02179	
5.88	1.046971	1.028664	0.019943	0.02621	
6.86	1.043774	1.022822	0.014221	0.006518	
7.84	1.022469	1.019153	0.017404	0.018051	
8.82	1	1	0	0	
10.65	2.746158	0.899947	0.452825	0.021645	
11.63	2.327446	0.898489	0.296322	0.004322	
12.61	1.828946	0.895792	0.157786	0.02259	
13.59	1.629643	0.871761	0.138832	0.0121	
14.57	1.362395	0.864726	0.063863	0.034841	
15.55	1.263634	0.85521	0.066758	0.0092	

16.53	1.138797	0.845511	0.096973	0.01878	
17.51	1.067498	0.843156	0.029635	0.007168	
18.49	1.014606	0.828597	0.012163	0.019187	
19.47	1.002487	0.823213	0.031252	0.006082	
20.45	0.961107	0.811516	0.005449	0.019017	
21.43	0.969088	0.793364	0.017498	0.019216	
22.41	0.929849	0.790722	0.020254	0.011199	
23.39	0.890637	0.788073	0.015394	0.014377	
24.37	0.913971	0.78372	0.034085	0.016752	
25.35	0.915498	0.768163	0.023124	0.023399	
26.33	0.877031	0.754982	0.012545	0.034016	
27.31	0.872501	0.759811	0.049671	0.025052	
28.29	0.857767	0.74523	0.008656	0.030388	
29.27	0.847154	0.741091	0.027807	0.012781	
30.25	0.810463	0.742974	0.022737	0.01386	
31.23	0.818498	0.739269	0.018192	0.019542	
32.21	0.833008	0.723439	0.010011	0.027376	
33.19	0.96258	0.718509	0.238676	0.026185	
34.17	0.813005	0.713234	0.033476	0.030348	
35.15	0.786543	0.696017	0.040151	0.018103	
36.13	0.798231	0.689526	0.036476	0.036593	
37.11	0.796213	0.679965	0.043199	0.017081	
38.09	0.767255	0.671968	0.045749	0.017953	
39.07	0.740614	0.670024	0.043103	0.026832	
40.05	0.746016	0.657043	0.054866	0.02399	
**Fig 6C. V2+V3**					
(n=3)	Mean		S.D.		Statistical method used
Time(min)	SDF1α	Veh.	SDF1α	Veh.	unpaired Student's t-test
0	0.942628	0.944891	0.054878	0.064598	
0.98	0.980891	1.011528	0.059116	0.054355	
1.96	1.00043	1.034334	0.03802	0.029246	
2.94	1.031605	1.033222	0.041882	0.006263	
3.92	1.023837	1.038017	0.041042	0.031227	
4.9	1.039644	1.044483	0.023615	0.027734	
5.88	1.04978	1.049475	0.061567	0.02738	
6.86	1.028516	1.074652	0.046352	0.10323	
7.84	1.021902	1.019115	0.015835	0.035649	
8.82	1	1	0	0	
10.65	4.366635	0.915886	0.609019	0.015066	
11.63	3.673783	0.911497	0.546885	0.018335	
12.61	2.918128	0.896845	0.369875	0.04751	
13.59	2.434502	0.886335	0.330831	0.027376	
14.57	1.924276	0.873505	0.207359	0.038591	
15.55	1.866474	0.859423	0.234354	0.030919	
16.53	1.70716	0.873243	0.205024	0.069563	
17.51	1.674996	0.869551	0.100008	0.05548	
18.49	1.489001	0.839602	0.241279	0.029835	
19.47	1.407908	0.829542	0.177193	0.045784	
20.45	1.398774	0.870561	0.094548	0.053354	
21.43	1.318838	0.835062	0.125985	0.022738	
22.41	1.225587	0.817917	0.08514	0.029926	
23.39	1.179243	0.793882	0.154085	0.042557	
24.37	1.086479	0.797353	0.045647	0.026527	
25.35	1.109341	0.792275	0.114666	0.034712	
26.33	1.049084	0.772184	0.090778	0.039419	
27.31	0.984945	0.787204	0.047006	0.033363	
28.29	1.042508	0.774878	0.077882	0.029133	
29.27	0.958211	0.770948	0.067279	0.02983	
30.25	0.960955	0.75699	0.070806	0.026943	
31.23	0.920594	0.763827	0.045503	0.035041	
32.21	0.958502	0.752041	0.154278	0.042878	
33.19	0.895359	0.747814	0.069449	0.032099	
34.17	0.92764	0.784724	0.086322	0.069824	
35.15	0.930392	0.736922	0.147183	0.057906	
36.13	0.862583	0.727818	0.046165	0.037645	

37.11	0.803258	0.713769	0.023587	0.045501	
38.09	0.806552	0.698126	0.026582	0.053606	
39.07	0.842433	0.699595	0.124233	0.044752	
40.05	0.861948	0.690284	0.046033	0.034355	
**Fig 6C. V2+V5**					
(n=3)	Mean		S.D.		Statistical method used
Time(min)	SDF1α	Veh.	SDF1α	Veh.	unpaired Student's t-test
0	0.999496	0.957033	0.018976	0.047378	
0.98	1.023563	1.004553	0.007354	0.041805	
1.96	1.048428	1.01925	0.049392	0.044063	
2.94	1.064044	1.16473	0.018038	0.160431	
3.92	1.0521	1.088418	0.027841	0.050799	
4.9	1.140197	1.083085	0.16485	0.044527	
5.88	1.086981	1.054444	0.095252	0.034186	
6.86	1.052508	1.023224	0.038619	0.026778	
7.84	1.024278	1.033864	0.011911	0.030199	
8.82	1	1	0	0	
10.65	3.389397	0.957414	0.608393	0.027325	
11.63	3.163475	0.945314	0.596964	0.037258	
12.61	2.466124	0.907037	0.258736	0.020513	
13.59	2.098758	0.921349	0.247733	0.00756	
14.57	1.79803	0.903903	0.174201	0.009307	
15.55	1.579716	0.898719	0.116921	0.020613	
16.53	1.459471	0.891124	0.032397	0.037195	
17.51	1.414249	0.879531	0.078451	0.022295	
18.49	1.295045	0.862281	0.035531	0.034104	
19.47	1.187685	0.864901	0.087504	0.012938	
20.45	1.262163	0.869871	0.141472	0.023763	
21.43	1.109354	0.853904	0.043218	0.006819	
22.41	1.092725	0.827321	0.080097	0.011216	
23.39	1.062273	0.888379	0.108534	0.074425	
24.37	0.963062	0.852641	0.045845	0.014774	
25.35	0.947835	0.82284	0.070599	0.019621	
26.33	0.931209	0.850095	0.072238	0.039089	
27.31	0.954521	0.875539	0.077334	0.05771	
28.29	0.900231	0.853998	0.029824	0.036939	
29.27	0.85499	0.846104	0.052395	0.071572	
30.25	0.943571	0.836135	0.025528	0.055571	
31.23	0.825094	0.827095	0.080847	0.046299	
32.21	0.815765	0.796301	0.071786	0.065308	
33.19	0.798176	0.795046	0.053307	0.075557	
34.17	0.80431	0.779063	0.098667	0.067164	
35.15	0.790152	0.781654	0.042587	0.065038	
36.13	0.796323	0.787854	0.076252	0.07714	
37.11	0.788508	0.797012	0.11016	0.08563	
38.09	0.78364	0.790808	0.084771	0.026596	
39.07	0.748713	0.852909	0.055301	0.085502	
40.05	0.706519	0.783226	0.087673	0.055669	

(n=3)	Mean	S.D.	Statistical method used	P value
**Fig 6C**			unpaired Student's t-test	**p<0.01<br>*p<0.05
vector	3.997794	0.221645		
V1	2.746158	0.155271		
V3	4.366635	0.154042		
V5	3.389397	0.264856		

(n=3)	Mean		S.D.		Statistical method used	P value
Days	SDF1α	Veh.	SDF1α	Veh.	unpaired Student's t-test	**p<0.01<br>*p<0.05
**Fig 7A. WT**						
1	1	1	0	0		
2	1.212842	1.120171	0.017819	0.017231		
3	1.531929	1.159446	0.075506	0.124595		
4	2.002829	1.382280	0.164187	0.245917		
**Fig 7A. KO/Vector**						

1	1	1	0	0		
2	0.960946	0.921364	0.038038	0.042019		
3	0.614413	0.53116	0.02248	0.062116		
4	0.421154	0.391944	0.033429	0.089434		
**Fig 7A. KO/V1**						
1	1	1	0	0		
2	1.000074	0.895451	0.030746	0.020563		
3	0.896439	0.570834	0.003904	0.023934		
4	0.929224	0.463704	0.047079	0.066002		
**Fig 7A. KO/V2**						
1	1	1	0	0		
2	1.140972	0.929244	0.058486	0.026644		
3	1.100348	0.670429	0.041594	0.030569		
4	1.265719	0.640323	0.032053	0.102444		
**Fig 7A. KO/V3**						
1	1	1	0	0		
2	1.247532	0.966194	0.052337	0.084074		
3	1.246003	0.715596	0.149452	0.021355		
4	1.477827	0.581338	0.075392	0.118926		
**Fig 7A. KO/V4**						
1	1	1	0	0		
2	0.983933	0.945584	0.021638	0.042154		
3	0.718031	0.674645	0.071228	0.052919		
4	0.528218	0.425378	0.030396	0.033213		
**Fig 7A. KO/V5**						
1	1	1	0	0		
2	1.119731	1.007941	0.079002	0.055032		
3	1.197022	0.729082	0.103501	0.018874		
4	1.246969	0.562408	0.086202	0.061248		

**Fig 7B**															
(n=3)	Mean							S.D.							Statistical method used
Time(h)	WT	KO	V1	V2	V3	V4	V5	WT	KO	V1	V2	V3	V4	V5	unpaired Student's t-test
0	100	100	100	100	100	100	100	100	100	100	100	100	100	100	
12	60.513	91.513	86.253	80.952	83.034	89.725	86.79	60.513	91.513	86.253	80.952	83.034	89.725	86.79	
24	38.205	81.505	72.322	64.603	61.023	78.982	72.346	38.205	81.505	72.322	64.603	61.023	78.982	72.346	
36	10.077	72.077	58.391	43.333	39.259	68.164	60.37	10.077	72.077	58.391	43.333	39.259	68.164	60.37	

**S1A. mGs**					
(n=3)	Mean		S.D.		Statistical method used
Time(min)	SDF1α	Veh.	SDF1α	Veh.	unpaired Student's t-test
0	1.099351	1.116452	0.019116	0.103074	
1.23	1.109826	1.121735	0.033366	0.048233	
2.46	1.088189	1.087471	0.029084	0.042844	
3.69	1.057129	0.95342	0.069626	0.038289	
4.92	1.006176	0.990246	0.049281	0.028813	
6.15	0.972266	0.989528	0.011483	0.036864	
7.38	0.900804	0.957238	0.036057	0.016058	
8.61	0.891274	0.929182	0.012677	0.042273	
9.84	0.874986	0.854729	0.048285	0.062762	
13.72	0.718484	0.705577	0.019365	0.028731	
14.95	0.69596	0.723158	0.035752	0.037196	
16.18	0.725211	0.760928	0.049375	0.030695	
17.41	0.701275	0.716209	0.019985	0.05621	
18.64	0.703652	0.679956	0.053048	0.013102	
19.87	0.701119	0.634326	0.05971	0.086801	
21.1	0.635523	0.689925	0.01541	0.070981	
22.33	0.729478	0.65926	0.102644	0.04132	
23.56	0.63244	0.654374	0.007295	0.093436	
24.79	0.590178	0.599767	0.049163	0.086271	
26.02	0.624078	0.578985	0.056591	0.035822	
27.25	0.626096	0.549191	0.06296	0.02189	

28.48	0.626035	0.591928	0.025533	0.052941	
29.71	0.590648	0.587738	0.048334	0.017305	
30.94	0.567208	0.598813	0.03811	0.028962	
32.17	0.58861	0.56148	0.057462	0.051768	
33.4	0.573734	0.584697	0.055502	0.045644	
34.63	0.555603	0.544818	0.062184	0.025276	
35.86	0.512831	0.522307	0.03965	0.119797	
37.09	0.484634	0.526054	0.0356	0.046869	
38.32	0.49852	0.54415	0.027884	0.051523	
39.55	0.507313	0.543221	0.010823	0.026941	
40.78	0.472102	0.514012	0.011461	0.041993	
42.01	0.502366	0.492835	0.029919	0.015912	
43.24	0.522375	0.481797	0.022254	0.051953	
44.47	0.483221	0.48891	0.059539	0.035268	
45.7	0.444549	0.497087	0.021005	0.012424	
46.93	0.454154	0.47094	0.042232	0.030881	
48.16	0.446369	0.458086	0.039808	0.034231	
49.39	0.454872	0.482091	0.043766	0.04652	
50.62	0.456372	0.422906	0.032993	0.021427	
51.85	0.448463	0.422002	0.040727	0.02351	
53.08	0.424031	0.422145	0.027305	0.055381	
54.31	0.448358	0.422731	0.019678	0.061778	
55.54	0.431062	0.427573	0.005237	0.03163	
56.77	0.444987	0.432487	0.013151	0.022789	
58	0.432788	0.430982	0.020042	0.061703	
59.23	0.40725	0.386646	0.044384	0.013785	
60.46	0.36512	0.420666	0.040782	0.033981	
61.69	0.332236	0.436886	0.008954	0.005547	
62.92	0.374261	0.426148	0.072759	0.005337	
64.15	0.398973	0.398737	0.056127	0.022523	
65.38	0.379388	0.394571	0.024689	0.06455	
66.61	0.331692	0.384508	0.024429	0.040283	
67.84	0.34467	0.356168	0.026561	0.020226	
69.07	0.393499	0.37894	0.025379	0.06036	
70.3	0.330387	0.364583	0.029195	0.021735	
71.53	0.367882	0.336864	0.030384	0.059703	
72.76	0.34982	0.338281	0.014442	0.001971	

**S1A. mGsq**					
(n=3)	Mean		S.D.		Statistical method used
Time(min)	SDF1α	Veh.	SDF1α	Veh.	unpaired Student's t-test
0	1.091035	1.108159	0.051922	0.044136	
1.23	1.069049	1.11807	0.072562	0.034568	
2.46	1.09095	1.071364	0.024818	0.033454	
3.69	1.047166	1.05021	0.017524	0.033822	
4.92	1.010001	0.962493	0.031392	0.038288	
6.15	0.99941	0.92911	0.047111	0.068874	
7.38	0.906422	0.975371	0.059535	0.037985	
8.61	0.930289	0.868468	0.033836	0.067054	
9.84	0.855677	0.916755	0.027959	0.067391	
13.72	0.78253	0.688478	0.084943	0.052545	
14.95	0.784853	0.761046	0.010356	0.085628	
16.18	0.768998	0.724885	0.045099	0.045669	
17.41	0.702151	0.687971	0.041745	0.05645	
18.64	0.674392	0.720736	0.032556	0.026279	
19.87	0.692602	0.672032	0.02041	0.03741	
21.1	0.682933	0.61564	0.044409	0.066951	
22.33	0.732438	0.673632	0.032838	0.009532	
23.56	0.667567	0.661999	0.032423	0.05897	
24.79	0.646859	0.641307	0.024154	0.083395	
26.02	0.599528	0.68689	0.042623	0.018282	
27.25	0.646895	0.637055	0.07346	0.026922	
28.48	0.673883	0.609229	0.019983	0.023059	
29.71	0.573112	0.587997	0.041172	0.044456	
30.94	0.606959	0.577928	0.051007	0.007193	
32.17	0.616449	0.59523	0.033258	0.031547	

33.4	0.570707	0.566771	0.03312	0.042063	
34.63	0.589203	0.597386	0.060034	0.079678	
35.86	0.543556	0.564077	0.026046	0.073157	
37.09	0.56819	0.555496	0.027965	0.047761	
38.32	0.535045	0.578966	0.041615	0.06692	
39.55	0.522376	0.524674	0.025973	0.017802	
40.78	0.525812	0.536551	0.059749	0.026505	
42.01	0.488072	0.498737	0.033205	0.028158	
43.24	0.519917	0.467711	0.027116	0.004475	
44.47	0.471251	0.526356	0.043381	0.034163	
45.7	0.482926	0.492624	0.029846	0.03849	
46.93	0.491328	0.504192	0.017928	0.024848	
48.16	0.474155	0.460398	0.025079	0.037065	
49.39	0.427331	0.460051	0.027242	0.036016	
50.62	0.474044	0.501231	0.032038	0.041065	
51.85	0.442812	0.432227	0.006865	0.005292	
53.08	0.448845	0.428061	0.013655	0.053537	
54.31	0.431076	0.417979	0.07073	0.043033	
55.54	0.466915	0.456264	0.036772	0.017188	
56.77	0.449957	0.464213	0.048218	0.028582	
58	0.443206	0.422655	0.007849	0.024416	
59.23	0.414121	0.465549	0.02546	0.018069	
60.46	0.378605	0.394867	0.036752	0.041572	
61.69	0.397669	0.433095	0.017498	0.010184	
62.92	0.413955	0.409478	0.004904	0.016814	
64.15	0.406067	0.39088	0.057368	0.027393	
65.38	0.372285	0.416665	0.018379	0.024154	
66.61	0.369598	0.416112	0.051348	0.04616	
67.84	0.355893	0.387068	0.05049	0.029756	
69.07	0.34015	0.41523	0.023959	0.073988	
70.3	0.364078	0.384307	0.027487	0.031358	
71.53	0.381111	0.392993	0.00851	0.018174	
72.76	0.349779	0.351706	0.037648	0.018172	
**S1A. mGsi**					
(n=3)	Mean		S.D.		Statistical method used
Time(min)	SDF1α	Veh.	SDF1α	Veh.	unpaired Student's t-test
0	1.134618	1.109393	0.090296	0.048268	
1.23	1.095957	1.098471	0.02237	0.054352	
2.46	1.020295	1.017494	0.054791	0.060583	
3.69	1.014485	1.044517	0.044316	0.014775	
4.92	0.965497	0.969028	0.062286	0.055293	
6.15	1.016818	0.975417	0.053683	0.034654	
7.38	0.914548	0.934134	0.086255	0.036222	
8.61	0.95054	0.972341	0.069548	0.04042	
9.84	0.887242	0.879205	0.061521	0.03624	
13.72	1.676688	0.708381	0.161432	0.055646	
14.95	1.800245	0.688746	0.100438	0.04709	
16.18	1.845916	0.726802	0.218328	0.069682	
17.41	1.797198	0.722973	0.243647	0.026738	
18.64	1.717907	0.712458	0.179186	0.048178	
19.87	1.610207	0.690395	0.207302	0.041846	
21.1	1.547581	0.66072	0.164734	0.039054	
22.33	1.38693	0.650329	0.180054	0.010778	
23.56	1.395691	0.655767	0.161603	0.064456	
24.79	1.265638	0.633177	0.159507	0.02449	
26.02	1.170789	0.655557	0.141819	0.037233	
27.25	1.11652	0.643965	0.089492	0.025445	
28.48	1.099983	0.59769	0.008132	0.017083	
29.71	1.050646	0.562327	0.090915	0.032931	
30.94	0.982684	0.53498	0.071272	0.012141	
32.17	0.988631	0.566246	0.091778	0.064675	
33.4	0.930486	0.607534	0.060048	0.047699	
34.63	0.926401	0.58255	0.026098	0.041186	
35.86	0.896906	0.545417	0.015609	0.042323	
37.09	0.803486	0.523101	0.034697	0.019205	

38.32	0.806015	0.546637	0.091072	0.026752	
39.55	0.813615	0.509611	0.025103	0.044588	
40.78	0.753639	0.528913	0.061212	0.092033	
42.01	0.698479	0.513929	0.076812	0.026361	
43.24	0.754248	0.525727	0.05472	0.037156	
44.47	0.731946	0.478866	0.057826	0.051337	
45.7	0.659138	0.443686	0.025223	0.026632	
46.93	0.654218	0.488638	0.007794	0.066596	
48.16	0.636275	0.478891	0.047286	0.041923	
49.39	0.62253	0.474201	0.071651	0.056232	
50.62	0.578173	0.44273	0.034281	0.029595	
51.85	0.632153	0.478904	0.060012	0.036542	
53.08	0.588795	0.445374	0.036057	0.019515	
54.31	0.531388	0.474132	0.082373	0.031672	
55.54	0.533717	0.457647	0.033183	0.059807	
56.77	0.536438	0.467461	0.020343	0.026907	
58	0.538891	0.443589	0.091088	0.016617	
59.23	0.527305	0.426256	0.064102	0.058166	
60.46	0.458898	0.420004	0.046007	0.052146	
61.69	0.511698	0.378973	0.029717	0.059416	
62.92	0.468978	0.378014	0.034375	0.052711	
64.15	0.50831	0.455034	0.093706	0.051846	
65.38	0.478631	0.379063	0.046597	0.018435	
66.61	0.429385	0.393546	0.02244	0.040736	
67.84	0.469192	0.382329	0.058746	0.035635	
69.07	0.450774	0.35051	0.031276	0.027102	
70.3	0.42065	0.374118	0.018518	0.030238	
71.53	0.425047	0.358896	0.034956	0.022187	
72.76	0.403298	0.378379	0.011607	0.030216	
**S1A. mG12**					
(n=3)	Mean		S.D.		Statistical method used
Time(min)	SDF1α	Veh.	SDF1α	Veh.	unpaired Student's t-test
0	1.139947	1.139356	0.059553	0.080926	
1.23	1.008602	1.089945	0.016433	0.022583	
2.46	1.049955	1.040525	0.062891	0.017054	
3.69	1.075228	1.02459	0.043755	0.019719	
4.92	1.002913	0.988153	0.044896	0.087153	
6.15	0.957071	1.004848	0.014522	0.05698	
7.38	0.928216	0.933069	0.037445	0.060168	
8.61	0.964499	0.930292	0.092131	0.060773	
9.84	0.87357	0.849222	0.055644	0.084151	
13.72	0.717423	0.717378	0.067285	0.012349	
14.95	0.707267	0.663943	0.016521	0.036052	
16.18	0.714179	0.687486	0.009103	0.037247	
17.41	0.681181	0.669405	0.02719	0.0938	
18.64	0.67174	0.670492	0.023559	0.046198	
19.87	0.659166	0.63358	0.031699	0.014114	
21.1	0.712697	0.63974	0.086314	0.061877	
22.33	0.693126	0.606688	0.059945	0.043206	
23.56	0.652377	0.623961	0.059854	0.043333	
24.79	0.66492	0.588265	0.083005	0.026488	
26.02	0.616245	0.581975	0.087418	0.050198	
27.25	0.586325	0.572618	0.038618	0.050365	
28.48	0.622209	0.592143	0.090557	0.050219	
29.71	0.569673	0.545646	0.044754	0.026631	
30.94	0.545419	0.584185	0.044157	0.012684	
32.17	0.595073	0.529719	0.042329	0.049602	
33.4	0.561	0.470831	0.024447	0.027819	
34.63	0.548995	0.516737	0.057295	0.030109	
35.86	0.607089	0.494321	0.065864	0.026969	
37.09	0.512386	0.512976	0.086241	0.032768	
38.32	0.53105	0.488827	0.043472	0.059594	
39.55	0.562536	0.504835	0.018591	0.041733	
40.78	0.516272	0.513367	0.064429	0.022172	
42.01	0.513422	0.43474	0.079823	0.050195	

43.24	0.518959	0.456887	0.043579	0.040959	
44.47	0.502454	0.475505	0.087487	0.064634	
45.7	0.528853	0.46941	0.05786	0.018615	
46.93	0.520828	0.467888	0.027363	0.034094	
48.16	0.483241	0.425556	0.061628	0.028221	
49.39	0.431594	0.433969	0.027453	0.035727	
50.62	0.490032	0.415326	0.03816	0.015505	
51.85	0.492953	0.391391	0.026647	0.036811	
53.08	0.454268	0.40437	0.01916	0.031675	
54.31	0.478022	0.395963	0.0277	0.039663	
55.54	0.462213	0.422517	0.02963	0.023044	
56.77	0.440014	0.39208	0.076665	0.014584	
58	0.437539	0.433435	0.019315	0.047025	
59.23	0.474493	0.396992	0.059283	0.018682	
60.46	0.426607	0.381819	0.051133	0.066581	
61.69	0.402447	0.368797	0.076828	0.039021	
62.92	0.40752	0.36123	0.076026	0.020635	
64.15	0.380231	0.363549	0.020343	0.063941	
65.38	0.407589	0.357027	0.078277	0.025273	
66.61	0.433156	0.345206	0.107173	0.052811	
67.84	0.39207	0.364648	0.024775	0.033847	
69.07	0.382039	0.336034	0.02087	0.034752	
70.3	0.392242	0.32818	0.062638	0.058139	
71.53	0.359799	0.353381	0.01672	0.032953	
72.76	0.391814	0.342083	0.055885	0.01337	
**S1A. mG16**					
(n=3)	Mean		S.D.		Statistical method used
Time(min)	SDF1α	Veh.	SDF1α	Veh.	unpaired Student's t-test
0	1.158143	1.139071	0.068471	0.04433	
1.23	1.107991	1.167255	0.087569	0.108398	
2.46	1.036677	1.071774	0.029362	0.080749	
3.69	0.997973	1.023588	0.077968	0.050306	
4.92	1.04787	0.988509	0.056589	0.056273	
6.15	0.963103	0.949364	0.106741	0.071327	
7.38	0.904295	0.931599	0.034718	0.018331	
8.61	0.91901	0.887872	0.042398	0.049814	
9.84	0.864938	0.840969	0.088687	0.078845	
13.72	0.706537	0.71991	0.035207	0.038022	
14.95	0.706398	0.65579	0.029479	0.054567	
16.18	0.699718	0.736099	0.091101	0.015712	
17.41	0.694725	0.644471	0.055507	0.035649	
18.64	0.641205	0.621455	0.076507	0.040506	
19.87	0.628262	0.670087	0.018743	0.072043	
21.1	0.667008	0.649096	0.057	0.059551	
22.33	0.625811	0.649522	0.073022	0.083208	
23.56	0.579314	0.627719	0.024273	0.037381	
24.79	0.612345	0.589503	0.022764	0.039035	
26.02	0.604041	0.6579	0.033955	0.071559	
27.25	0.596662	0.570458	0.04128	0.078218	
28.48	0.550884	0.569211	0.080509	0.042397	
29.71	0.56085	0.565395	0.066578	0.056026	
30.94	0.568993	0.553891	0.061288	0.028098	
32.17	0.528276	0.529225	0.016319	0.058507	
33.4	0.545088	0.520755	0.024524	0.035261	
34.63	0.507467	0.478632	0.048482	0.072417	
35.86	0.551914	0.532572	0.080312	0.031349	
37.09	0.54274	0.467758	0.037584	0.041957	
38.32	0.562758	0.4944	0.045637	0.049169	
39.55	0.480222	0.466559	0.035374	0.013921	
40.78	0.46334	0.506293	0.044375	0.036615	
42.01	0.460948	0.449869	0.025881	0.070918	
43.24	0.462973	0.485774	0.011983	0.039934	
44.47	0.459087	0.488357	0.069399	0.021746	
45.7	0.485653	0.44831	0.050378	0.007932	
46.93	0.465575	0.444923	0.111963	0.053993	

48.16	0.432798	0.392189	0.034715	0.061848	
49.39	0.422847	0.477131	0.050361	0.073655	
50.62	0.432531	0.415304	0.054854	0.026342	
51.85	0.402015	0.432581	0.073764	0.039806	
53.08	0.394556	0.350445	0.025469	0.06457	
54.31	0.350537	0.355536	0.047983	0.036768	
55.54	0.396573	0.405081	0.018595	0.021453	
56.77	0.448021	0.398072	0.070712	0.02976	
58	0.404439	0.424849	0.023154	0.029155	
59.23	0.375848	0.383959	0.05381	0.0152	
60.46	0.371005	0.374711	0.032862	0.04633	
61.69	0.347348	0.349503	0.029265	0.037131	
62.92	0.358379	0.357176	0.051195	0.046439	
64.15	0.407877	0.332599	0.043855	0.037448	
65.38	0.367258	0.375861	0.005894	0.070727	
66.61	0.350866	0.363834	0.043633	0.023548	
67.84	0.341189	0.38797	0.046163	0.020801	
69.07	0.348804	0.345706	0.058922	0.016186	
70.3	0.313961	0.330672	0.022377	0.024998	
71.53	0.37713	0.338363	0.082475	0.059098	
72.76	0.297298	0.318593	0.03211	0.011316	

**S1B. mGs**					
(n=3)	Mean		S.D.		Statistical method used
Time(min)	SDF1α	Veh.	SDF1α	Veh.	unpaired Student's t-test
0	0.956745	1.142369	0.306443	0.265243	
1.23	1.089499	1.024476	0.361496	0.070316	
2.46	1.149876	0.87824	0.08314	0.108419	
3.69	1.095315	1.005212	0.116772	0.296521	
4.92	1.115643	0.893126	0.092477	0.230069	
6.15	1.011081	0.95132	0.017554	0.111803	
7.38	0.870512	1.064899	0.168267	0.109635	
8.61	0.836063	1.029246	0.194008	0.259057	
9.84	0.875266	1.011113	0.37476	0.068301	
13.72	0.820729	0.683664	0.082309	0.038045	
14.95	0.859227	0.830838	0.21607	0.226128	
16.18	0.816322	0.750203	0.066036	0.145713	
17.41	0.680802	0.807425	0.064543	0.210018	
18.64	0.649377	0.717182	0.035924	0.1809	
19.87	0.676002	0.964898	0.039134	0.233567	
21.1	0.661375	0.808024	0.327555	0.199239	
22.33	0.707126	0.763885	0.189347	0.232585	
23.56	0.710019	0.80926	0.187171	0.018599	
24.79	0.682127	0.630167	0.177537	0.045312	
26.02	0.649019	0.627421	0.114565	0.009971	
27.25	0.644288	0.6094	0.123166	0.214832	
28.48	0.67286	0.702824	0.131218	0.171541	
29.71	0.500217	0.542225	0.063827	0.081966	
30.94	0.600967	0.53191	0.117838	0.196926	
32.17	0.534982	0.665825	0.15337	0.071052	
33.4	0.717985	0.65659	0.056656	0.255732	
34.63	0.440004	0.620452	0.081341	0.166922	
35.86	0.582516	0.583235	0.138273	0.099835	
37.09	0.599428	0.698813	0.115874	0.269074	
38.32	0.524495	0.46447	0.031134	0.162403	
39.55	0.556209	0.648123	0.222076	0.234079	
40.78	0.493608	0.488237	0.102174	0.131076	
42.01	0.52961	0.636647	0.030609	0.167535	
43.24	0.532019	0.469222	0.216803	0.076082	
44.47	0.582	0.615264	0.182735	0.09073	
45.7	0.542742	0.591482	0.240726	0.157775	
46.93	0.552724	0.504037	0.050845	0.094327	
48.16	0.577358	0.55931	0.191782	0.221687	
49.39	0.492114	0.555452	0.008255	0.117855	
50.62	0.52137	0.420264	0.15016	0.17291	

51.85	0.502179	0.589344	0.053155	0.022937	
53.08	0.454488	0.427942	0.092717	0.178105	
54.31	0.455403	0.502251	0.089991	0.123117	
55.54	0.355246	0.491602	0.055637	0.114133	
56.77	0.450128	0.468311	0.10333	0.014103	
58	0.482582	0.476581	0.086638	0.077303	
59.23	0.450497	0.541967	0.14937	0.225761	
60.46	0.413248	0.447592	0.100131	0.14218	
61.69	0.456252	0.579819	0.069149	0.290472	
62.92	0.562946	0.531611	0.089954	0.162752	
64.15	0.526025	0.524008	0.156816	0.223614	
65.38	0.373976	0.412747	0.044657	0.060811	
66.61	0.434417	0.463757	0.05612	0.090177	
67.84	0.40251	0.569896	0.095264	0.163862	
69.07	0.406786	0.453686	0.178942	0.192312	
70.3	0.42103	0.396221	0.106588	0.142675	
71.53	0.550516	0.460435	0.22293	0.371616	
72.76	0.438318	0.425403	0.307277	0.247586	
**S1B. mGsq**					
(n=3)	Mean		S.D.		Statistical method used
Time(min)	SDF1α	Veh.	SDF1α	Veh.	unpaired Student's t-test
0	1.057316	1.298645	0.3901	0.21655	
1.23	1.179913	1.055589	0.189641	0.237657	
2.46	1.14539	0.93469	0.117006	0.09087	
3.69	0.923266	1.058198	0.110347	0.30848	
4.92	0.987864	1.061356	0.107918	0.132504	
6.15	0.941881	0.980102	0.090723	0.109521	
7.38	0.831377	0.817939	0.183813	0.113415	
8.61	0.989908	0.91103	0.209102	0.12296	
9.84	0.943086	0.882452	0.182762	0.218776	
13.72	0.806125	0.713947	0.046176	0.078059	
14.95	0.777123	0.88502	0.236544	0.256444	
16.18	0.787079	0.653565	0.170182	0.147143	
17.41	0.596514	0.589619	0.297863	0.073891	
18.64	0.629638	0.649091	0.054611	0.111534	
19.87	0.672906	0.694309	0.053843	0.14793	
21.1	0.893479	0.575941	0.116495	0.268203	
22.33	0.581167	0.605916	0.2546	0.1031	
23.56	0.641052	0.647639	0.140421	0.23554	
24.79	0.523479	0.591903	0.088036	0.058338	
26.02	0.753395	0.475755	0.078898	0.091733	
27.25	0.634175	0.551412	0.184265	0.105926	
28.48	0.743827	0.496942	0.275212	0.063616	
29.71	0.699379	0.581813	0.184471	0.106798	
30.94	0.562882	0.576848	0.119454	0.041535	
32.17	0.817204	0.695821	0.371004	0.356891	
33.4	0.628779	0.469841	0.20846	0.01367	
34.63	0.461101	0.571232	0.097184	0.160169	
35.86	0.583992	0.46111	0.028076	0.16025	
37.09	0.540861	0.514457	0.124417	0.154125	
38.32	0.652769	0.518793	0.182524	0.097378	
39.55	0.599704	0.580774	0.125486	0.321387	
40.78	0.465464	0.443645	0.025412	0.030562	
42.01	0.684461	0.610478	0.054192	0.277132	
43.24	0.53734	0.532402	0.168476	0.068463	
44.47	0.576242	0.544843	0.107604	0.147373	
45.7	0.442702	0.568041	0.152798	0.040573	
46.93	0.586695	0.460663	0.196704	0.026702	
48.16	0.481066	0.497984	0.161549	0.119131	
49.39	0.419508	0.639085	0.109764	0.284642	
50.62	0.602507	0.516465	0.057984	0.008194	
51.85	0.383599	0.535824	0.120146	0.054615	
53.08	0.414212	0.53013	0.100041	0.033319	
54.31	0.533281	0.419146	0.172725	0.082548	
55.54	0.559861	0.392575	0.116731	0.08275	

56.77	0.342379	0.531611	0.02481	0.218463	
58	0.502796	0.388499	0.115193	0.03531	
59.23	0.502392	0.407538	0.096646	0.164241	
60.46	0.749072	0.39339	0.250354	0.027211	
61.69	0.552481	0.450394	0.081948	0.124346	
62.92	0.401012	0.380292	0.143353	0.050855	
64.15	0.605568	0.442663	0.145975	0.151517	
65.38	0.494051	0.447464	0.094911	0.210817	
66.61	0.474601	0.337396	0.119899	0.124267	
67.84	0.482672	0.380839	0.028604	0.004322	
69.07	0.431999	0.492361	0.054989	0.251846	
70.3	0.33674	0.288913	0.065079	0.052249	
71.53	0.512628	0.433161	0.165967	0.120201	
72.76	0.634455	0.41605	0.248265	0.101498	
**S1B. mGsi**					
(n=3)	Mean		S.D.		Statistical method used
Time(min)	SDF1α	Veh.	SDF1α	Veh.	unpaired Student's t-test
0	1.144669	0.98739	0.073184	0.149671	
1.23	1.136545	1.175401	0.228337	0.224376	
2.46	1.056795	1.074099	0.074349	0.078374	
3.69	1.060894	0.918557	0.080172	0.110534	
4.92	0.9444	1.071359	0.052969	0.249935	
6.15	1.014033	0.996311	0.042307	0.118203	
7.38	0.909059	0.883012	0.17934	0.114811	
8.61	0.912156	0.875101	0.070461	0.27964	
9.84	0.821448	1.018771	0.204599	0.323328	
13.72	0.798719	0.939792	0.400813	0.138612	
14.95	0.619109	0.843439	0.049175	0.149463	
16.18	0.761156	0.616512	0.088697	0.16571	
17.41	0.903259	0.917661	0.191092	0.118005	
18.64	0.679664	0.881351	0.154367	0.142582	
19.87	0.845558	0.73013	0.170745	0.129418	
21.1	0.631234	0.707541	0.051052	0.386296	
22.33	0.619857	0.823485	0.169936	0.148624	
23.56	0.64603	0.60658	0.11531	0.23451	
24.79	0.791725	0.548379	0.313501	0.097798	
26.02	0.594399	0.684185	0.103337	0.057121	
27.25	0.531994	0.723067	0.104489	0.168327	
28.48	0.616564	0.729683	0.182599	0.123074	
29.71	0.736339	0.609638	0.0926	0.022536	
30.94	0.642037	0.635569	0.035223	0.127936	
32.17	0.413613	0.677782	0.148061	0.091541	
33.4	0.558882	0.475687	0.061966	0.151072	
34.63	0.680269	0.644613	0.154073	0.122103	
35.86	0.548318	0.709284	0.118279	0.16643	
37.09	0.535669	0.559305	0.159492	0.019608	
38.32	0.465713	0.714418	0.046175	0.170238	
39.55	0.591363	0.667882	0.157104	0.111449	
40.78	0.52067	0.713525	0.185221	0.234314	
42.01	0.42367	0.539735	0.139339	0.107535	
43.24	0.698506	0.62332	0.126505	0.138082	
44.47	0.402603	0.580327	0.121204	0.077043	
45.7	0.442783	0.547439	0.078326	0.168554	
46.93	0.527153	0.639505	0.025201	0.186227	
48.16	0.425851	0.809294	0.113634	0.27022	
49.39	0.470307	0.424378	0.147769	0.121693	
50.62	0.553166	0.435209	0.018024	0.102997	
51.85	0.559674	0.542532	0.07512	0.184314	
53.08	0.389261	0.509218	0.107599	0.057096	
54.31	0.619107	0.524047	0.082975	0.115362	
55.54	0.560409	0.57594	0.14797	0.177349	
56.77	0.414455	0.471535	0.130063	0.092486	
58	0.35804	0.561324	0.098039	0.067602	
59.23	0.438746	0.515869	0.060922	0.029233	
60.46	0.510382	0.422228	0.128586	0.27077	

61.69	0.625042	0.47159	0.05872	0.152855	
62.92	0.569523	0.471512	0.192127	0.147819	
64.15	0.294004	0.403291	0.044371	0.085251	
65.38	0.426868	0.543141	0.242543	0.142791	
66.61	0.417559	0.497285	0.069785	0.08416	
67.84	0.536574	0.368405	0.175423	0.026673	
69.07	0.463886	0.482281	0.126976	0.081341	
70.3	0.36856	0.377618	0.028684	0.10347	
71.53	0.433925	0.419138	0.143689	0.101747	
72.76	0.314122	0.27339	0.100476	0.103703	
**S1B. mG12**					
(n=3)	Mean		S.D.		Statistical method used
Time(min)	SDF1α	Veh.	SDF1α	Veh.	unpaired Student's t-test
0	1.297749	1.171464	0.166863	0.144524	
1.23	0.954404	1.273653	0.203985	0.093436	
2.46	1.225091	1.02122	0.193415	0.017223	
3.69	1.02561	0.890317	0.079779	0.231569	
4.92	0.947067	1.069571	0.228922	0.171449	
6.15	0.978016	0.856535	0.328903	0.066808	
7.38	0.833326	0.920685	0.171664	0.30467	
8.61	0.827169	0.980943	0.038392	0.033215	
9.84	0.911568	0.815614	0.220008	0.378838	
13.72	0.911672	0.782807	0.177885	0.204926	
14.95	0.843044	0.925523	0.100453	0.078715	
16.18	0.825617	0.833633	0.334139	0.259707	
17.41	0.79083	0.94627	0.355933	0.317221	
18.64	0.772996	0.628294	0.141319	0.12129	
19.87	0.719704	0.831372	0.340638	0.150036	
21.1	0.809171	0.595507	0.095403	0.091852	
22.33	0.6858	0.74791	0.148966	0.070836	
23.56	0.734973	0.717144	0.133351	0.267464	
24.79	0.845218	0.741628	0.170789	0.15313	
26.02	0.690398	0.791188	0.09146	0.152259	
27.25	0.526852	0.70958	0.046508	0.081171	
28.48	0.809277	0.751218	0.328995	0.203989	
29.71	0.799952	0.767154	0.157017	0.197322	
30.94	0.64044	0.75143	0.217292	0.356788	
32.17	0.752955	0.640851	0.103817	0.045884	
33.4	0.744057	0.693734	0.216315	0.233746	
34.63	0.772274	0.637188	0.123242	0.362257	
35.86	0.665945	0.880919	0.125351	0.369381	
37.09	0.789893	0.62716	0.168834	0.047577	
38.32	0.571274	0.723399	0.167524	0.281068	
39.55	0.569126	0.56339	0.076036	0.041469	
40.78	0.740676	0.705051	0.116105	0.1036	
42.01	0.729448	0.691854	0.165246	0.133958	
43.24	0.507063	0.631228	0.173262	0.193705	
44.47	0.51064	0.686609	0.189682	0.170607	
45.7	0.708388	0.744723	0.187671	0.154492	
46.93	0.552964	0.569501	0.093916	0.044038	
48.16	0.617832	0.54467	0.171595	0.083397	
49.39	0.477816	0.660151	0.113472	0.19962	
50.62	0.520063	0.40682	0.07332	0.127117	
51.85	0.665293	0.751588	0.063119	0.161891	
53.08	0.543019	0.494337	0.102145	0.045039	
54.31	0.470752	0.647683	0.110004	0.043516	
55.54	0.434114	0.448992	0.093113	0.194152	
56.77	0.65656	0.611625	0.03864	0.12842	
58	0.583997	0.657216	0.061369	0.069757	
59.23	0.5705	0.546889	0.197159	0.120437	
60.46	0.526938	0.619981	0.105053	0.056516	
61.69	0.51509	0.458688	0.066732	0.170275	
62.92	0.449628	0.576778	0.046009	0.226436	
64.15	0.623617	0.492878	0.195568	0.165611	
65.38	0.457771	0.499463	0.071772	0.084734	

66.61	0.548082	0.64465	0.040845	0.07582	
67.84	0.568547	0.62327	0.140406	0.185371	
69.07	0.466541	0.557486	0.05188	0.442573	
70.3	0.437292	0.458826	0.206696	0.124719	
71.53	0.49485	0.471277	0.261623	0.149913	
72.76	0.429109	0.480693	0.112032	0.142394	
**S1B. mG16**					
(n=3)	Mean		S.D.		Statistical method used
Time(min)	SDF1α	Veh.	SDF1α	Veh.	unpaired Student's t-test
0	1.091594	0.996179	0.323953	0.219125	
1.23	1.098696	1.237531	0.133015	0.164874	
2.46	1.158885	1.155125	0.045855	0.135629	
3.69	1.069629	0.983141	0.125724	0.104497	
4.92	0.985793	1.079673	0.151382	0.023899	
6.15	0.786407	0.850269	0.06889	0.060219	
7.38	1.010934	0.974313	0.120368	0.078087	
8.61	0.825838	0.990399	0.319733	0.177728	
9.84	0.972223	0.733369	0.107875	0.089187	
13.72	0.773728	0.772187	0.140446	0.168641	
14.95	0.679072	0.631409	0.135511	0.11802	
16.18	0.651049	0.780015	0.260863	0.124191	
17.41	0.762141	0.606055	0.212065	0.221764	
18.64	0.706339	0.596172	0.037864	0.053732	
19.87	0.67748	0.796991	0.045514	0.194596	
21.1	0.678912	0.606652	0.025387	0.131473	
22.33	0.665686	0.776215	0.187786	0.300723	
23.56	0.715272	0.692731	0.099665	0.409232	
24.79	0.682096	0.655173	0.248689	0.041464	
26.02	0.700628	0.600387	0.011816	0.096497	
27.25	0.796088	0.644259	0.185212	0.140601	
28.48	0.698383	0.640718	0.120651	0.318058	
29.71	0.575162	0.588762	0.119434	0.106156	
30.94	0.586211	0.68686	0.211796	0.207629	
32.17	0.463034	0.475963	0.032543	0.014079	
33.4	0.618214	0.584579	0.173461	0.207855	
34.63	0.641755	0.630047	0.076265	0.128361	
35.86	0.652968	0.426022	0.181427	0.15273	
37.09	0.686358	0.505744	0.115895	0.095631	
38.32	0.618471	0.691791	0.01951	0.181775	
39.55	0.508353	0.564169	0.120274	0.142684	
40.78	0.45559	0.617157	0.106239	0.147796	
42.01	0.635247	0.564299	0.062696	0.099567	
43.24	0.664279	0.558466	0.109486	0.013091	
44.47	0.50224	0.544779	0.033196	0.134351	
45.7	0.609084	0.559602	0.138978	0.121201	
46.93	0.527054	0.524222	0.117009	0.083751	
48.16	0.42744	0.527616	0.096671	0.087863	
49.39	0.508132	0.524605	0.012961	0.130903	
50.62	0.644078	0.487562	0.178878	0.23189	
51.85	0.532596	0.384023	0.051063	0.040761	
53.08	0.55751	0.452141	0.162412	0.102579	
54.31	0.298052	0.437851	0.02782	0.111933	
55.54	0.502804	0.503077	0.115273	0.195472	
56.77	0.507873	0.518631	0.115277	0.115694	
58	0.406084	0.367904	0.054165	0.038759	
59.23	0.426873	0.50375	0.072932	0.018493	
60.46	0.491973	0.324221	0.162949	0.030421	
61.69	0.487333	0.379462	0.060378	0.208021	
62.92	0.406072	0.488693	0.156106	0.150942	
64.15	0.556126	0.429057	0.137153	0.096679	
65.38	0.436159	0.403082	0.106964	0.098895	
66.61	0.551462	0.418211	0.171433	0.192441	
67.84	0.389355	0.557349	0.111363	0.119714	
69.07	0.403137	0.460613	0.099972	0.145714	
70.3	0.381159	0.380638	0.115307	0.052304	

71.53	0.531162	0.415814	0.198741	0.2076	
72.76	0.360466	0.357723	0.021929	0.00912	

(n=3)	Mean		S.D.		Statistical method used
Time(h)	SDF1α	Veh.	SDF1α	Veh.	unpaired Student's t-test
**S2. WT**					
0	0.3036	0.3259	0.013553	0.016183	
12	0.433767	0.5	0.008424	0.064759	
24	0.7563	0.871467	0.033892	0.057247	
36	1.5142	1.696233	0.047964	0.046682	
**S2. KO**					
0	0.363367	0.391467	0.026591	0.008053	
12	0.6151	0.728767	0.028651	0.042805	
24	1.2916	1.4279	0.040708	0.089022	
36	2.4097	2.4899	0.044935	0.127935	
**S2. V1**					
0	0.413633	0.435733	0.010945	0.040763	
12	0.725567	0.812333	0.053844	0.016703	
24	1.476933	1.616967	0.061545	0.069456	
36	2.6513	2.704433	0.056273	0.049446	
**S2. V2**					
0	0.424167	0.447867	0.007506	0.02535	
12	0.664467	0.806033	0.05565	0.030373	
24	1.405233	1.489133	0.101195	0.073557	
36	2.3953	2.5846	0.181613	0.071566	
**S2. V3**					
0	0.481733	0.519067	0.009502	0.013155	
12	0.814633	0.939167	0.049933	0.035649	
24	1.7525	1.833567	0.095688	0.086185	
36	2.692433	2.807333	0.051247	0.076269	
**S2. V4**					
0	0.449867	0.470533	0.024141	0.011378	
12	0.737933	0.8147	0.015943	0.05228	
24	1.593167	1.666833	0.077344	0.024382	
36	2.608467	2.712167	0.043387	0.100041	
**S2. V5**					
0	0.378733	0.406133	0.018064	0.005907	
12	0.5839	0.675933	0.009708	0.004917	
24	1.1413	1.300467	0.014441	0.045613	
36	2.354433	2.4237	0.03503	0.118489	
