# Supplementary material for: The N-terminus of CXCR4 splice variants determines expression and functional properties
Source: PLoS One. 2023 May 4;18(5):e0283015. doi: 10.1371/journal.pone.0283015 (PMC10159351; doi:10.1371/journal.pone.0283015)

Fig 1C

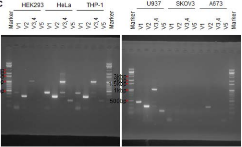

taken by Canon PowerShot G12.

V1: 445bp (V1-59F/V1-504R)
V2: 321bp (V2-43F/V1-585R)
V3: 437bp (V34-140F/V1-585R)
V4: 323bp (V34-140F/V1-585R)
V5: 220bp (V5-258F/V5-478R)

Fig 2D

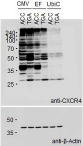

Fig 2E

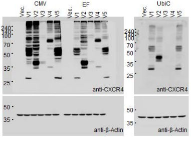

Fig 2G

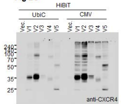

captured by eBLOT Touch Imager.

Fig 3D

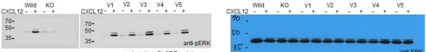

captured by eBLOT Touch Imager.

scanned by HP officejet pro 8710.

Fig 5A

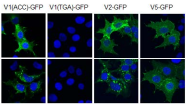

captured by confocal LSM800 microscope
(Carl Zeiss Microimaging Inc., Zena, Germany).

Fig 5B

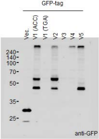

captured by eBLOT Touch Imager.

Fig 6B

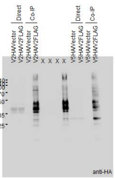

captured by eBLOT Touch Imager.

S3 Fig

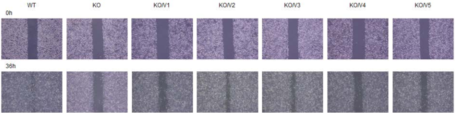

Supplement: S1 Raw images — (PDF) [file pone.0283015.s002.pdf]
