# Supplementary figures and images for: The N-terminus of CXCR4 splice variants determines expression and functional properties
Source: PLoS One. 2023 May 4;18(5):e0283015. doi: 10.1371/journal.pone.0283015 (PMC10159351; doi:10.1371/journal.pone.0283015)

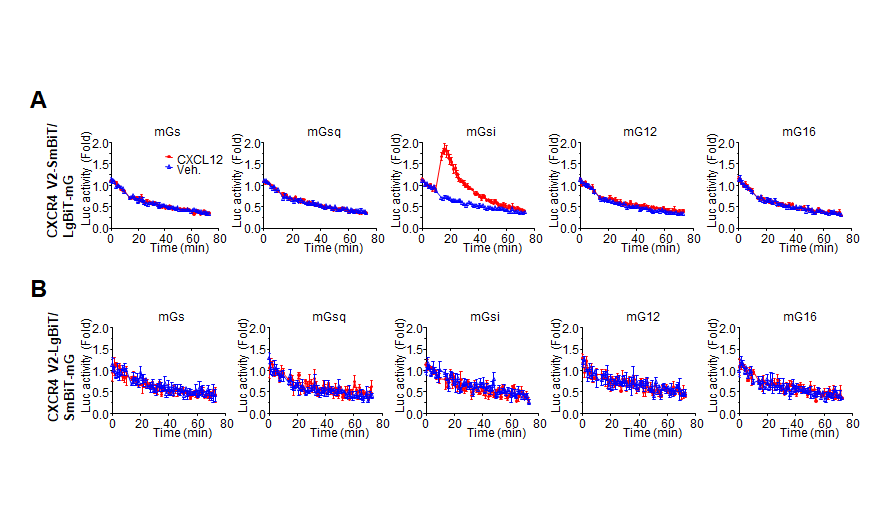

Supplement: S1 Fig — HEK293 cells expressing C-terminal SmBiT (A) or LgBiT (B) form of CXCR4 variant2 with each of mini G protein constructs tagged with LgBiT or SmBiT were treated with 100 ng. ml CXCL12. The luminescence changes were measured with luminometer. (TIF) [file pone.0283015.s003.tif]

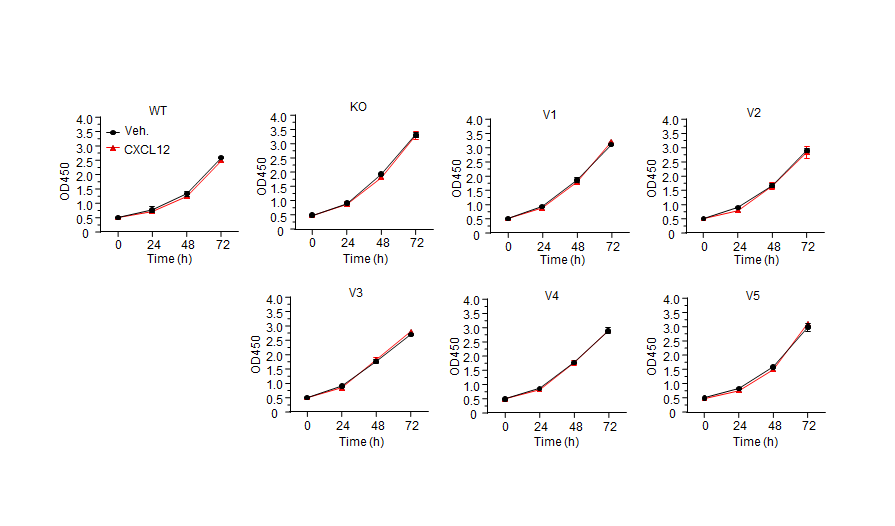

Supplement: S2 Fig — Different types of HeLa cells, such as parental cells, CXCR4 KO cells, and cells reconstituted with each splicing variant of CXCR4, were seeded in 96-well plates at a density of 4 × 10³ cells and cultured in complete media containing 10% FBS with or without 100 ng/ml CXCL12. Every 24 h, cells were subjected to CCK-8 assay. (TIF) [file pone.0283015.s004.tif]

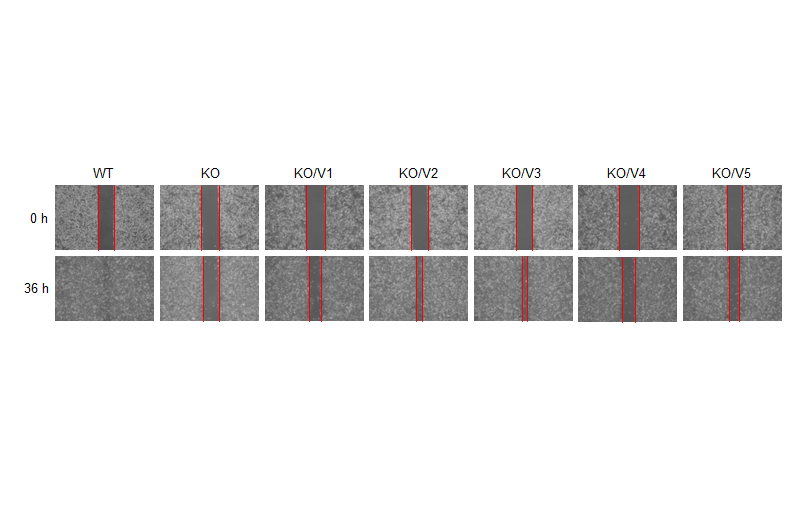

Supplement: S3 Fig — Confluent monolayers of parental HeLa cells and genetically modified HeLa cells were scratched with pipette tips. After washing with PBS to remove floating cells, media containing 10% FBS with 100 ng/ml CXCL12 was added to cell monolayers. The images were captured at 0 h and 36 h. (TIF) [file pone.0283015.s005.tif]
